# Future-proofing genomic data and consent management: a comprehensive review of technology innovations

**DOI:** 10.1093/gigascience/giae021

**Published:** 2024-06-05

**Authors:** Adrien Oliva, Anubhav Kaphle, Roc Reguant, Letitia M F Sng, Natalie A Twine, Yuwan Malakar, Anuradha Wickramarachchi, Marcel Keller, Thilina Ranbaduge, Eva K F Chan, James Breen, Sam Buckberry, Boris Guennewig, Matilda Haas, Alex Brown, Mark J Cowley, Natalie Thorne, Yatish Jain, Denis C Bauer

**Affiliations:** Australian e-Health Research Centre, Commonwealth Scientific and Industrial Research Organisation, Level 3/160 Hawkesbury Rd, Westmead NSW 2145, Australia; Australian e-Health Research Centre, Commonwealth Scientific and Industrial Research Organisation, Level 3/160 Hawkesbury Rd, Westmead NSW 2145, Australia; Australian e-Health Research Centre, Commonwealth Scientific and Industrial Research Organisation, Level 3/160 Hawkesbury Rd, Westmead NSW 2145, Australia; Australian e-Health Research Centre, Commonwealth Scientific and Industrial Research Organisation, Level 3/160 Hawkesbury Rd, Westmead NSW 2145, Australia; Australian e-Health Research Centre, Commonwealth Scientific and Industrial Research Organisation, Level 3/160 Hawkesbury Rd, Westmead NSW 2145, Australia; Responsible Innovation Future Science Platform, Commonwealth Scientific and Industrial Research Organisation, Brisbane, 41 Boggo Rd, Dutton Park QLD 4102, Australia; Australian e-Health Research Centre, Commonwealth Scientific and Industrial Research Organisation, Level 3/160 Hawkesbury Rd, Westmead NSW 2145, Australia; Data61, Commonwealth Scientific and Industrial Research Organisation, Level 5/13 Garden St, Eveleigh NSW 2015, Australia; Data61, Commonwealth Scientific and Industrial Research Organisation, Building 101, Clunies Ross St, Black Mountain, Canberra, ACT 2601, Australia; NSW Health Pathology, Sydney, 1 Reserve Road, St Leonards NSW 2065, Australia; Telethon Kids Institute, Perth, WA 6009, Australia; National Centre for Indigenous Genomics, The John Curtin School of Medical Research, Australian National University, Canberra, ACT 2601, Australia; Telethon Kids Institute, Perth, WA 6009, Australia; National Centre for Indigenous Genomics, The John Curtin School of Medical Research, Australian National University, Canberra, ACT 2601, Australia; Sydney Medical School, Brain and Mind Centre, The University of Sydney, Sydney, 94 Mallett St, Camperdown NSW 2050, Australia; Australian Genomics, Parkville, VIC 3052, Australia; Murdoch Children’s Research Institute, Parkville, Victoria 3052, Australia; Telethon Kids Institute, Perth, WA 6009, Australia; National Centre for Indigenous Genomics, The John Curtin School of Medical Research, Australian National University, Canberra, ACT 2601, Australia; Children’s Cancer Institute, Lowy Cancer Research Centre, Level 4, Lowy Cancer Research Centre Corner Botany & High Streets UNSW Kensington Campus UNSW Sydney, Kensington NSW 2052, Australia; School of Clinical Medicine, UNSW Medicine & Health, Wallace Wurth Building (C27), Cnr High St & Botany St, UNSW Sydney, Kensington NSW 2052, Australia; University of Melbourne, Melbourne, Parkville VIC 3052, Australia; Melbourne Genomics Health Alliance, Melbourne 1G, Walter and Eliza Hall Institute/1G Royal Parade, Parkville VIC 3052, Australia; Walter and Eliza Hall Institute, Melbourne, 1G, Walter and Eliza Hall Institute/1G Royal Parade, Parkville VIC 3052, Australia; Australian e-Health Research Centre, Commonwealth Scientific and Industrial Research Organisation, Level 3/160 Hawkesbury Rd, Westmead NSW 2145, Australia; Applied BioSciences, Faculty of Science and Engineering, Macquarie University, Applied BioSciences 205B Culloden Rd Macquarie University, NSW 2109, Australia; Applied BioSciences, Faculty of Science and Engineering, Macquarie University, Applied BioSciences 205B Culloden Rd Macquarie University, NSW 2109, Australia; Department of Biomedical Sciences, MQ Health General Practice - Macquarie University, Suite 305, Level 3/2 Technology Pl, Macquarie Park NSW 2109, Australia; Australian e-Health Research Centre, Commonwealth Scientific and Industrial Research Organisation, Gate 13, Kintore Avenue University of Adelaide, Adelaide SA 5000, Australia

**Keywords:** genome data privacy, trust model, decentralized systems, self-sovereign identity, dynamic consent

## Abstract

Genomic information is increasingly used to inform medical treatments and manage future disease risks. However, any personal and societal gains must be carefully balanced against the risk to individuals contributing their genomic data. Expanding our understanding of actionable genomic insights requires researchers to access large global datasets to capture the complexity of genomic contribution to diseases. Similarly, clinicians need efficient access to a patient’s genome as well as population-representative historical records for evidence-based decisions. Both researchers and clinicians hence rely on participants to consent to the use of their genomic data, which in turn requires trust in the professional and ethical handling of this information.

Here, we review existing and emerging solutions for secure and effective genomic information management, including storage, encryption, consent, and authorization that are needed to build participant trust. We discuss recent innovations in cloud computing, quantum-computing-proof encryption, and self-sovereign identity. These innovations can augment key developments from within the genomics community, notably GA4GH Passports and the Crypt4GH file container standard. We also explore how decentralized storage as well as the digital consenting process can offer culturally acceptable processes to encourage data contributions from ethnic minorities.

We conclude that the individual and their right for self-determination needs to be put at the center of any genomics framework, because only on an individual level can the received benefits be accurately balanced against the risk of exposing private information.

## Introduction

Over 60 million individuals are estimated to have their genomes sequenced in a health care context by 2025 [1]. This increase can be attributed to the decreasing cost of genome sequencing [2–5], the rise of direct-to-consumer genetic testing companies [6], the integration of genome testing into public health care systems, and the launch of large-scale population genomics initiatives in numerous countries [7–11]. However, digital infrastructure, software solutions, data security measures, and legal frameworks for managing big genomic data have not kept pace with these rapid advancements. Notably lacking are advancements in ethical data management, efficient data sharing, and data sovereignty [12]. Addressing these aspects is essential to ensure the continued participation of a privacy-aware public, especially from marginalized communities, in contributing their private information to research [13, 14].

The challenges become even more pronounced when applying genomics in a clinical context. Here, the generated data directly impact patient care while also having value in broadening scientific knowledge. Clinicians typically rely on pathology providers to generate reports summarizing the genomic information to inform patient care. These reports are generated by multidisciplinary teams [15], but in the remaining article, we will refer to them as “pathology providers” for simplicity. While established clinical governance and security standards provide guidance for the management, storage, and analysis of genomic data for clinical care, it often conflicts with the need for broader access and sharing of this data for research [15]. Platforms able to serve both clinical and research applications need to resolve the tension between the protective regulations in clinical settings and the exploratory objectives of research, as well as enable interoperability across the 2 domains.

Key concerns in genomic data management are privacy attacks that exploit an individual’s sensitive health and ancestry information, particularly when clinically generated genomic data are reused for research purposes. For instance, *identification attacks* can link an individual’s genomic data with publicly available information, such as demographic data or family history, to triangulate on target individuals [16, 17]. Current research practices of masking personally identifying variants, such as rare single nucleotide polymorphisms or germline variants, are not sufficiently protective [18]. Even if the genomic sequence is not ascertained directly, in a *membership inference attack*, the adversary can infer the membership status of individuals in genomic research studies, such as rare disease genome-wide association studies (GWAS), by leveraging allele frequencies from public databases [19, 20]. This risk is exacerbated with the increasing number of large-scale national or regional studies that recruit all participants who meet broad eligibility criteria [21]. This also extends to an individual’s physical traits, demographic information, and disease susceptibility, which can be obtained through *phenotype inference attacks* using genomic data [22, 23]. In these studies, whole genomic sequencing and detailed phenotyping were used to predict biometric traits, including voice, biological age, and 3-dimensional facial structure [23].

To generate scientific outcomes that are robust, clinically meaningful, ethical, and equitable, genomic data need to have ethnically diverse representation [24]. However, to achieve this diversity, it is essential to acknowledge that the sociocultural context of genomic data management extends beyond individual perspectives and involves collective experiences and histories that can shape attitudes toward genomic data sharing. This is especially pertinent for historical instances of discrimination, trauma, racism, stigma, and marginalization [25]. These collective experiences and cultural connotations significantly influence an individual’s or population’s perception of the risks associated with genomic data management, creating an intricate landscape that navigates the potential misuse of these data against the interests of certain groups or populations.

Enabling a safe way forward, legislation will have to provide active governance and enforce ethical genomic data usage as started by the Health Insurance Portability and Accountability Act (HIPAA) [26] and others [27, 28]. This is especially difficult around participants’ right to be forgotten, so far only required by General Data Protection Regulation (GDPR) [26, 29], which can be at odds with commercial incentives. For example, while the Australian Financial Services Council specified in their Life Code in July 2023 that genetic results cannot be used in underwriting certain life insurance policies [30], this is not the case in other countries or sectors, leaving individuals vulnerable to disadvantages from intended or incidental findings of genetic testing.

Given these complexities, a strong and trusted technological foundation for genomic data governance and management is crucial. We explore both proven and emerging solutions and concepts in this review and suggest a framework based on decentralized identity concepts for genomic data and consent management.

## Genomic Data Storage Solutions

Secure data storage is essential, for genomic and health care data [31–33], specifically on cybersecurity issues such as data breaches, unauthorized access, or malicious attacks [34]. The key advantages and disadvantages of the 4 approaches reviewed are outlined in Table 1.

**Table 1:** Different storage solutions’ key advantages and disadvantages for effective genomic data management

Criteria	On-premises	Cloud	Hybrid	Decentralized
Security	Low risk of attacks when operating offline.	Physical control over servers through contractual agreements, but state-of-the-art audited cybersecurity.	Flexibility to adapt to sensitivity levels.	Technology theoretically renders attacks on stored data impossible.
Scalability	Slow adaption to changes and sudden spikes in workload.	Excels in adapting to dynamic changes and handling sudden increases in workload efficiently.	Balanced solution to manage varying workload.	Scalability is native to the solution.
Data backups	Need to be implemented by the organization, requiring skills, and scaling up the infrastructural capabilities.	Automated data backups immediately upon the upload of data.	Depends on the architecture deployed.	Built-in data back-up feature.
Skills required	A singular set of specific skills is requisite.	A singular set of specific skills is requisite.	Deploying an optimal system necessitates twice the skillset.	Still experimental requires nonstandard skill set
Data control	Complete control and access to servers, data, and associated rights by the data custodian.	Comprehensive control and access to data, servers, and related privileges within a virtualized environment.	Depends on the architecture deployed.	No control over where data are stored but full control of access by participants.
Price	Involves an initial and ongoing capital investment, maintenance, and considerations for expenses related to operational costs, potential equipment failures, and associated bills.	With various storage options (cold/hot), the cost is economical and free for idle time.	Depends on the architecture deployed.	Can be the most economical choice, but for certain options, both pricing and reliability are contingent on the value of the associated token.

### On-premises storage

On-premises data management refers to storing and managing data within the physical premises of an organization, providing complete control over data infrastructure, customizing storage environment, and meeting its unique needs. Storing on-premises can be highly secure when there is no external network as data access can be physically limited to only authorized personnel. This hence represents an easy option for organizations to comply with data privacy and security regulations [26, 27, 29]. However, it also creates undesirable information silos, especially for the health care setting. Data integration and global research collaborations need carefully managed exposure to the Internet [35], which requires significant expertise, constant monitoring, and substantial time and resource investment to establish and oversee security protocols. In addition, on-premises data management requires an upfront investment and replacement at relatively frequent 3- to 5-year intervals for the necessary infrastructure, disadvantaging smaller organizations. Ongoing costs such as energy expenses, broadband access, software licenses, certifications, IT services, and physical space to accommodate the hardware must also be considered. Furthermore, on-premises storage is not suited for short-term spikes in workload [36] as the infrastructure is static and expansion or update is expensive and time-consuming.

### Cloud storage

Data owners can store and manage their data with a public cloud provider [37]. As organizations do not build and manage their infrastructure, capital investments are shifted toward resource consumption and recorded as operational expenses. This allows organizations to pay and scale infrastructure to their changing needs but can clash with CapEx-based funding cycles. While legislation to keep medical data inside the countries’ jurisdiction has had limited cloud usage in the past, cloud providers have responded by opening more in-country data centers and enabling policy configurations that ensure data and back-up remain compliant [38, 39]. For example, governments use such policy-optimized clouds for their operations (e.g., AWS GovCloud [40] and AZURE Government [41]). Furthermore, managing data and analysis in the cloud enables seamless global collaborations and ensures reproducible results.

However, this scalability and convenience comes with a higher security risk for the data as the uniformity of cloud account structures makes them attractive targets for hackers [42–45]. To mitigate security risks, cloud providers implement automatic countermeasures and equip users with world-class security measures, including access controls through IP address restriction, continuous threat monitoring, encryption for data in transit and at rest, network and application security, data redundancy, and multifactor authentication [46–49]. While the economy of scale stems most of the costs for security, scalability, and global connectedness, cloud usage can become expensive, especially for egress-heavy applications and for users who do not implement auto-archiving retention policies to take advantage of low-cost cloud storage options (like AWS Glacier or Azure Archive). Alternatively, egress cost can be avoided altogether by using federated systems, where compute is brought to the data, for example, recently employed by UK Biobank Research Access Platform [50].

Finally, building health care critical infrastructure on—for many users—foreign national cloud providers raises concerns around sovereignty and the limits to oversight, which is exacerbated when data are collected in the clinical setting and reused in research. Public cloud use hence needs to be carefully balanced against the benefits that a globally connected economic health system can provide.

For example, Melbourne Genomics Health Alliance, a collaboration of leading Victorian hospitals, research institutions, and academic institutions working with the Victorian government to embed genomics into the health system, developed a cloud solution that benefits from international developments in security, scalability, and health system interoperability, yet seeks to minimize third-party dependence by implementing federated data governance controls. Specifically, the genomic information management system, Genomical (previously known as GenoVic [51]), offers a robust clinical data governance framework and implemented capability for controlled access and reuse of data between authorized entities for the purpose of clinical care, therefore providing foundations for data reuse within the health care system where it is adopted.

### Hybrid storage

Hybrid storage solutions can provide the best of both worlds—combining the benefits of on-premises and cloud data management solutions. To adopt such an approach, organizations require a workforce skilled in both domains. By leveraging hybrid solutions, organizations can maintain a local infrastructure for sensitive data and analytics, while easily connecting storage to additional resources in the public cloud when needed, such as processing spiky analysis workloads, or genomic data processing pipelines that require different computing types. However, it comes at the expense of egress costs, potential duplication of effort for system maintenance, and limited access to clinically generated data for health care or research purposes.

It allows a staged transition to the cloud where scalability and global connectivity can be realized, without the need to move all data to the cloud at once. This also ensures sovereignty by maintaining some compute and data storage capabilities.

On-premises and cloud computing infrastructure are hence complementary pieces of the puzzle that can help research organizations achieve their goals.

For instance, the Australian Zero Childhood Cancer Program [52] houses genomic data on a cloud-connected NetApp StorageGRID within a dedicated partition in an Equinix data center (see www.equinix.com). This configuration enables standardized data sharing via object store protocols, allowing integration with cloud providers, genomic analysis platforms like CAVATICA (see www.cavatica.org), and national high performance computing resources. Through automated archiving, processed data are retained on-site, reducing long-term cloud storage costs, and data are shared with researchers through unique and secure s3 links. The program mitigates the system’s egress costs by routing network traffic through academic networks like AARNet [53] where possible and leveraging Equinix Fabric for global collaborations.

### Decentralized storage

Decentralized storage is a Web3 concept [54] where files are fragmented, encrypted, and stored over separate nodes in a decentralized or peer-to-peer (P2P) network [55]. By distributing data across different nodes, it is more secure than being stored in a single “honey pot.” It also improves scalability and availability over on-premises and cloud solutions, as the P2P network can be continuously expanded with commodity hardware that is easy to onboard.

The InterPlanetary File System (IPFS) [56] was one of the first decentralized file storage systems developed and originally used as the storage layer for blockchains. IPFS uses cryptographic hashes that are based on the content of the file, thereby eliminating duplicates and ensuring data integrity. IPFS can store and share massive amounts of data in a decentralized and economical manner, which is crucial for genomic projects [57–59]. However, unlike traditional data centers, decentralized file systems are not funded through a single entity. While more traditional blockchain approaches have a built-in incentive structure [60], IPFS relies on goodwill from the P2P network notes and is, therefore, not suitable for operating critical infrastructure, such as health care.

FileCoin is a separate and independent decentralized protocol built on IPFS that is incentivized to offer their storage space by receiving digital currencies as a reward. Various protocols ensure the integrity, security, availability, and accessibility of the data stored on the network [61].

While these incentives are aimed to ensure quality and make storage sustainable, it is crucial to acknowledge the potential risks associated with this approach. Fraudulent projects and initial coin offerings have exploited users in the past by marketing themselves as investment options [62, 63]. The risk of being used as a speculative commodity poses a significant risk to the stability of decentralized services, as the digital currencies market value can undermine the platform’s incentive structure and functioning. Specifically, if the value of a coin declines, the motivation for nodes to continue storing data ceases, resulting in the loss of irreplaceable medical information. Given these observations, it is crucial to have a careful technical, economic, and ethical evaluation of those systems, especially in the health space.

## Genomic Data Privacy and Security

Irrespective of where genomic data are stored, the individual needs to be protected from unauthorized access to their data (privacy) and the data need to be kept safe from threats, breaches, and unauthorized tampering (security). We explore this topic under the criteria of data availability, integrity, and confidentiality (AIC) [64], sometimes also referred to as the “CIA triad.”

### Availability

Availability ensures timely and uninterrupted access to the genomic data system by authorized users only. Human genomic data are generally protected, and access is only approved if relevant requirements are met. While clinical data can be managed by clinical governance principles, the viability of data usage in research requires data access committees (DACs) to review access requests and ensure that the intended use of the data is permitted by the provided consent [65]. All public genomic data repositories work on this premise, including the database of genotypes and phenotypes, the European Genome-Phenome Archive, and the UK Biobank [66, 67].

While this protects data and participants, it is a manual process that is not easily scalable, making datasets hard to discover and limiting their use for clinical or research benefits. The Global Alliance for Genomics and Health (GA4GH [68]) has introduced the third access tier, apart from existing open access and controlled access, called “registered access” to automate consent mechanisms and address some of the problems. The registered access [69] tier is intended to allow access to low-risk data for research use, and it requires the user to be a “bona fide researcher,” in addition to agreeing to the terms of use for the data.

To support automation and interoperability across multiple data systems, the GA4GH has developed the GA4GH Passports and Authentication & Authorization Infrastructure (AAI) specifications [70]. Passports provide the format for data and resource access permissions, built on top of the openID Connect standards [71], which in turn are based on the OAuth 2.0 framework [72]. They serve as AAI tokens (using JSON Web Tokens) to carry a researcher’s digital identity and access rights across organizations, tools, and environments, encoding each permission/claim as “Visas” and allowing access to specific registered-access datasets. Visas are issued if the intended use of the data complies with restrictions set out by the DAC. Uses and restrictions are based on defined terms in the GA4GH Data Use Ontology [73], fully automating the whole process and potentially saving months between an access request and approval.

Availability can be compromised by both nonmalicious factors (such as hardware failures, software downtime, resource limits, unmanaged concurrency, network congestion, and natural disaster) and malicious attacks (such as denial of service, also known as DoS attacks) that aim to disrupt the system’s functionality. Therefore, technical and operational security measures such as redundancy, backup, load balancing, and encryption are also essential to protect the system from threats to availability.

### Integrity and privacy-preserving techniques

The second pillar of AIC [64], data integrity practices, aims to store and handle the data to prevent accidental or unauthorized modification throughout its entire life cycle. Genomic data can be efficiently verified and compared by matching hash values using deterministic, collision-resistant, and noninvertible [74] cryptographic hash [75] functions such as Message Digest and the Secure Hash Algorithm [76]. This allows for the identification of any unauthorized changes or alterations to genetic sequences without the need to compare the entire sequence. Furthermore, data hash values can be cryptographically signed [77] using a private key. This digital signature can be verified using the corresponding public key, ensuring the data’s authenticity, integrity, and trustworthiness.

The privacy of the genomic data can be protected through 4 approaches summarized in Table 2. It should be noted that this section focuses on the research setting as in health care, patient information cannot be obfuscated.

**Table 2:** Advantages and disadvantages of different privacy preservation approaches

Criteria	*k* -anonymity	Differential privacy	Federated learning	Synthetic data methods
Implementation complexity	Simple and intuitive. Data are grouped in sets to obfuscate individual entries.	Challenging implementation due to the intricate calibration of noise.	Challenges in coordinating local model and with network latency, trust, and incentive among peers.	Challenging to conservatively maintain the statistical properties of real genomic data but works as real data statistically.
Privacy assurance	Cannot fully prevent attribute disclosure or homogeneity attack.	Robust privacy is achieved through the addition of statistical noise to the data, necessitating the quantification of risks through careful control of the trade-off between utility and privacy.	The potential for data leakage risk arises from intermediate model updates, which may expose sensitive information. Additionally, there remains a possibility of backdoor attacks.	May not guarantee perfect privacy and vulnerability to membership inference even when generating distributions close to real data.
Accuracy/information loss	Homogeneity attacks pose a risk when individuals within a group may disclose sensitive information due to indistinguishability based on certain attributes.	Deliberate introduction of noise can reduce the quality or utility of the resultant data.	The original data remain unaltered and can only be removed by the owners or those with granted permissions. Can be subject to data poisoning attack due to involvement of dishonest peers.	Despite being synthetic and closely resembling real data, it can introduce bias and errors.

#### k-anonymity

The *k-*anonymity approach works by ensuring that the quasi-identifier for each person, such as their gender, birth date, postal code, race, ethnicity, or occupation, is indistinguishable from at least *k* − 1 individuals in the same dataset [78, 79]. This is done by using 2 approaches: (i) generalization, which groups individuals together with similar attributes [80], and (ii) suppression, which removes certain information to prevent reidentification. For example, one way to suppress genomic data is to remove germline variants, which are inherited from parents and can be used to link individuals across databases. However, this may not completely eliminate the risk of reidentification, as other types of variants or genomic features may still be informative [81, 82]. Despite their widespread use, these *k*-anonymity approaches are vulnerable to attackers who have background information on the dataset [83] and are limited for high-dimensional genomic data [84].

#### Differential privacy (DP)

Differential privacy (DP) is a mathematical framework that provides formal and provable privacy protection by introducing calibrated noise to raw data or intermediate results, making it difficult for attackers to trace data records to specific individuals [85]. The amount of noise added depends on various factors, including the query type, privacy budget determining the level of privacy required, and the sensitivity of the mathematical function being computed or the query output.

In genomics, DP techniques have been proposed to counteract membership inference attacks, for example, by adding noise to a genomic Beacon query response, the genome data discovery tool by GA4GH [86]. The amount of noise is carefully calibrated to balance 2 goals: to preserve the accuracy and hence the utility of the application and to make it harder for attackers to extract the original genomic data from the query response [87]. Additionally, DP methods for GWAS have provided maximum privacy to participants while still finding meaningful disease associations [88–90]. Although promising, the added noise in DP schemes limits its application to datasets with strong, well understood signals (e.g., disease loci with strong effect size).

#### Federated learning (FL)

Federated learning (FL) is a machine learning technique that enables multiple parties to jointly train an algorithm without sharing their data, thereby avoiding risks to data integrity or having to negotiate data access [91]. In this approach, computations are performed locally on the data that remain within the owner’s ecosystem (e.g., server nodes, jurisdictions). These locally trained parameters are then sent to a central server that aggregates the local models from all participating peers to generate a global model shared by all [92, 93]. Federated learning has been used on health data [94, 95] and was shown on genomics data to achieve comparable performance compared to a centralized approach for phenotype prediction on genomic data using the UK Biobank [96].

While promising, coordinating local model aggregates can be challenging, especially when training complex FL models. Issues such as network latency, maintaining trust and incentive among participating peers, and ensuring data quality and diversity remain unresolved. Further, FL is specifically vulnerable to data poisoning attacks, where attackers deliberately manipulate or corrupt the data, and backdoor attacks by poisoning models to output biased results [97].

#### Privacy-preserving synthetic genomic data

Creating synthetic genomic data can sidestep many data privacy issues. These data have the same statistical properties as the original dataset, but without passing on the real genomes. Several methods have been developed to generate synthetic genomes leveraging various sources of knowledge, including haplotype information [98, 99], demographic information, and recombination inferences [100]. More advanced methods like deep neural network–based methods, such as generative adversarial networks and restricted Boltzmann machines (RBMs), have also successfully generated synthetic genomic data where population structure and variant frequency–based features were preserved [101]. Generative methods can be used to create datasets that act as proxies for underrepresented populations, going some way to address the known Eurocentric bias in genomic studies [102]. However, the utility of synthetic genomes is limited to evaluating algorithms, rather than for discovery projects, because they do not have more information than the original data, which is further limited by the fidelity of the generative model used.

A recent study by Oprisanu et al. [103] compared the above synthetic data methods for utility and privacy. They showed that recombination-based methods have high utility but low privacy, while RBMs offer a trade-off. It is worth noting that generating distributions close to the real data often generates target data points that are vulnerable to membership inference [104]. Therefore, some data integrity and security practices must be enforced even for synthetic data generated by current approaches.

### Confidentiality and cryptography methods

Confidentiality represents the third pillar of AIC, which prevents unauthorized access or disclosure of data. We review the 5 most relevant and noteworthy techniques as summarized in Table 3.

**Table 3:** Advantages and disadvantages of different data security approaches

Criteria	Symmetric encryption	Asymmetric encryption	Multiparty computation	Homomorphic encryption	Post quantum
Scalable performance	Fast and efficient for large genomic datasets.	Slower for encrypting large-scale genomic data, potentially delaying data transfer.	Computationally expensive, making it less practical for real-time analysis of genomic data.	Well suited for cloud-based genomic data processing, but computationally intensive with prolonged processing times.	Difficult to scale currently for large-scale datasets.
Collaboration suitability	Less suitable if multiple parties are involved due to single key reliance.	The public key can be shared with collaborators, ensuring secure data transfers between institutions without compromising integrity.	Enables collaborative work among institutions without disclosing each other’s data.	Facilitates collaboration easily through cloud capabilities yet challenging to set up for all collaborations.	Potentially suitable but depends on the development of practical and efficient protocols that are easily adoptable by multiple parties.
Complexity	Straightforward cryptographic key management due to only 1 key involved.	Key management can be complicated for multiple parties.	Challenging setup complexity and dependence on collaborating parties.	Initial setup and implementation can be challenging.	Complex in terms of development and implementation; often requires significant expertise and resources to correctly deploy.
Robustness	Risky if key compromised in collaborative settings; secure with robust key management.	Risky if key compromised in collaborative settings; secure with robust key management.	Maximal collaboration security: genomic data consistently encrypted, demands participant trust to prevent data poisoning.	Great as it allows computations on encrypted data, without decrypting it first.	Robust defense against quantum attacks for long-term genomic data security, currently in developmental stages with limited adoption.

#### Encryption

Encryption is a cryptographic method that aims to secure genomic files by converting *plain text* to *cipher text* using different algebraic operations.

Symmetric encryption methods encrypt data using stream ciphers such as Salsa20, CHACHA20, and AES-CTR [105] or block ciphers such as the Advanced Encryption Standard (AES) [106] and are a popular method for securing genomic data as they are fast and efficient [107]. For large genomic data, CHACHA20 is the fastest and most efficient algorithm [108] and is often used in combination with POLY1305, a message authentication code, to ensure message integrity and authentication [109]. For example, CHACHA20-POLY1305 is used in Crypt4GH [110], a file container standard proposed by GA4GH. Its user-specific envelop encryption scheme enables random byte-level access to encrypted file content without decrypting the whole file. Block ciphers have also been used in genomic data encryption [111].

Asymmetric or public cryptography schemes, such as Rivest–Shamir–Adleman (RSA) and elliptic curve cryptography (ECC), use 2 keys for encryption: a public key for encryption and a private key for decryption. The RSA algorithm has been used for genomic data to mask individuals’ alleles and secure cloud-based genetic paternity test results [83, 112], but it can be slow and impractical for large Whole Genome Sequencing (WGS) files [112] and is therefore limited to smaller genomic files and sensitive metadata. ECC is often preferred over RSA for smaller genomic files due to its smaller key sizes and lower computational cost [113, 114].

#### Multiparty computation (MPC)

Multiparty computation (MPC) improves upon the traditional route of encrypting data solely for storage and transport purposes, which subsequently requires decryption and handling in an unencrypted manner during analysis. MPC frameworks allow researchers to collaborate on data analysis while maintaining privacy using cryptographic methods. While still an emerging technology, it has made rapid progress over the past few years with open-source frameworks such as MP-SPDZ [115], which combines several MPC variants with an accessible Python user geared toward analytics. Here, we review (i) secret sharing, (ii) garbled circuit method, (iii) homomorphic encryption, and (iv) zero-knowledge proof.

##### Secret sharing

A core technology to most MPC protocols is secret sharing, which denotes the distributed storage of information such that the parties together can reconstruct the information, but an individual party (or a small set) cannot. In some settings, secret-sharing techniques alone can be used for privacy-preserving analytics without the more expensive techniques below. These protocols have been found relatively efficient, enabling even the training of smaller deep learning models.

##### Garbled circuit method

A popular method of developing MPC is the garbled circuit method, where the function is transformed into a Boolean circuit of logic gates and encrypted to produce garbled output values [116]. Collaborators can then use garbled values and their input to generate an output, ensuring privacy and correctness. In the genomic space, the garbled circuit solution has been used for secure genomic data analysis [117, 118] and diagnosis [119], but this approach can be expensive for large genomic data.

##### Homomorphic encryption

Homomorphic encryption (HE) is a cryptographic technique that allows computations to be performed on encrypted data without decrypting them first. This means that the data can be processed securely without revealing any sensitive information to the parties involved. This can be useful for genome queries and statistical analyses such as GWAS, which aim to find genetic variants associated with certain traits or diseases. Several methods have been developed for HE-based genome queries and statistical analyses [120–124]. However, HE requires more computational resources and time than regular encryption methods, but new algorithms and techniques have been proposed to improve performance [125]. Combining HE with MPC is argued to further reduce overhead compared to traditional encryption methods. It has shown potential for encrypted control over genomic data to enhance trust within genomics research programs. Additionally, techniques that combine HE with differential privacy can be employed to ensure the security of genomic data and enable privacy while sharing summary results [126].

##### Zero-knowledge proof

Zero-knowledge proof (ZKP) is a cryptographic method allowing one party to prove a claim’s correctness to another party without revealing additional information [127]. In genomics, ZKP has been used to enable secure genomic query [128] and sequence similarity search [129]. ZKP methods can be hard to set up, requiring subject matter expertise, and they are not scalable due to high compute power on large datasets.

#### Postquantum cryptography

There is growing concern among information security experts that most of the currently available cryptographic methods, such as RSA and ECC, are vulnerable to attacks by quantum computers (e.g., Shor’s algorithm [130]). Although quantum computers are expensive and still developing, it is feared that attackers are already downloading sensitive data to decrypt in the future when quantum computers become more readily available [131]. This has significant implications for genomic data encrypted by today’s cryptographic methods as genomic data retain their relevancy over an individual’s life span and even that of their direct descendants.

In response, the National Institute of Standards and Technology in the United States has announced encryption algorithms that were designed to resist attacks from classical and quantum computers, thereby enabling postquantum migration of cryptosystems. These include lattice-based algorithms CRYSTALS-Kyber [132] for general-purpose encryption, as well as CRYSTALS-Dilithium [133] and FALCON [134], both for digital signatures. Using such postquantum cryptography methods for genomic data now is a proactive step to ensuring that genomic data are protected against possible future attacks while saving time and money rather than re-encrypting when quantum computers become more accessible.

## Informed Consent Management

Another key pillar of good data management is informed consent, which in the clinical space is well defined by following established clinical governance practices. However, when genomic data generated for clinical purposes are reused for research, its original consent may not be enough and reconsenting is required [135–138].

Different countries and regions can also have specific legal frameworks and guidelines pertaining to informed consent. Ideally, existing health care systems of record (e.g., My Health Record) can obtain and hold consent information so clinically generated data can be reused for research.

We will focus on 3 consent models: (i) broad, (ii) tiered, and (iii) dynamic consent, all of which aim to balance the participant’s control over their data with efficient data sharing [139].

### Consent models

#### Broad consent

The *broad* consent model is used for studies where the genomic data collected may additionally be used in other research unrelated to the original study for which the consent was given [140, 141]. This model is commonly used in large longitudinal biobanks and date from a time when it was complicated to keep contact with patients after the initial meeting. However, it poses ethical and legal challenges as individuals may not fully understand what all they are consenting to [142], in part due to the broad language used to cover future studies that have not yet been defined [140].

#### Tiered consent

Unlike the broad consent model, the tiered consent model at the outset provides participants with highly specific consent options [143]. For example, participants can choose to share specific genomic information or consent to participate in specific research studies only. However, the tiered consent model creates administrative and logistical challenges for researchers to comply with the different levels of consents and for participants to inform themselves upfront about the different options [139]. Contrasting with the one-off approach of broad consent, the tiered consent model may call for renewed consent for each new study or operate under a set of predefined conditions, dependent on the initial choices made by the participants.

#### Dynamic consent

The dynamic consent model focuses on enhancing continuous engagement of participants through personalized online consent processes (e.g., *dynamic specific consent* [139]) and digital communication platforms [144–147]. It is believed that dynamic consent positively influences both the recruitment and retention of participants as well as their trust toward research [144, 146, 148, 149], while also contributing to the proficient management of the informed consent procedure [144].

Building on this, *dynamic meta consent* enables participants to define rules to approve or reject studies without needing to decide on each study manually. For example, participants can define their preferences for data use (e.g., academic vs. commercial), data type (e.g., genomic data, medical records, imaging), research institution (e.g., universities, research labs), or funding sources (e.g., public or private) [150]. This approach provides participants with a fine-grained control over how their data are used yet eliminates the need to manage requests for each individual study. Akin to *tiered* consent, participants are also required to make upfront decisions at high levels of abstraction without the context for future research studies [139], but they have the flexibility to revise their decision dynamically as new information becomes available or as their preferences change.

### Digital systems for consent management

Traditionally, consent was obtained and recorded as a paper-based documentation. However, tiered and dynamic consent drive the adoption of digital systems where electronic consent forms enable participants to enter, manage, and withdraw their consent (e.g., through web portals or mobile applications) [151]. It can also allow authorized researchers/pathology providers to request access to the genomic and other health data located in the storage system for various purposes such as research or clinical decisions. However, whether data are stored in a research setting or under the custodianship of an accredited pathology company influences the approaches and policies of the digital system, which are managed by 3 components: (i) identity and access management (IAM), (ii) personalized consent elements, and (iii) information storage in the context of genomics research programs.

#### IAM component

The IAM component manages registration and authentication, allowing authorized users (participants/patients or researchers/pathology providers) access to the system and its resources. Users are assigned an “ID” for recognition, linking their genomic data and consent along with health records. GA4GH registered access and Passport standard can be repurposed for researchers’ interaction with the digital system.

#### Personalized consent materials

Especially useful for genomic research programs are technologies like interactive webpages and virtual or augmented reality to *personalize consent materials* for clear and engaging explanations of complex scientific concepts and of the program’s research aims to participants [152]. Language aids such as chatbots [153] and translation systems are also powerful tools to supplement this component for non-English-speaking participants. An ontology system can be integrated so that consent language can be transformed into machine-readable codes that tag datasets and manage data permissions [73, 154]. These elements are also helpful during consent process in a health care setting where the data might be used for secondary analysis.

#### Information storage component

As genomic research programs often handle health information as well, the *information storage component* can be integrated with data capture and management systems that comply with regulatory standards prescribed by sovereign privacy legislation such as GDPR, Australian Privacy Principles (APPs), and HIPAA to ensure that participants’ data and consent are securely stored. For instance, CTRL [155], an Australian Genomics dynamic consent platform, integrates with the REDCap [156] data capture system, a popular free regulatory-compliant data capture system, to collect and combine consent and research data. It should be noted that the level of security for the REDCap system resides with the provider and may hence vary in quality. Other alternatives such as Castor EDC, Qualtrics, and ClinCapture are available with more user-friendly interfaces and customer support.

Digital consent management is currently delivered predominantly through centralized systems, which facilitate access control, data stewardship, and policy governance. Such centralization brings the benefits of streamlined management and efficient consent workflows, reducing complexity for organizations. However, it imposes significant burdens on IT systems in synchronizing consent changes at all levels of data usage and demands intensive manual processes to demonstrate adherence to compliance standards [60, 157]. This added governance layer may inadvertently create procedural bottlenecks. These bottlenecks can lead to delays and inefficiencies that might not be directly visible to participants but could diminish their overall experience. As a result, there is risk of reduction in participant engagement and participation rate [145, 158].

#### Decentralized approaches

Decentralized dynamic consent management systems aim to overcome the limitations of centrally managed structures by delivering both the IAM and information storage components in a programmatically insured process. This allows real-time monitoring of data use, participant-executed revocation of data, and a tamper-proof record of consent changes. It also can cater for the remote or culturally appropriate collection of consent, such as the immutable collection of consent offline, or unfettered voting through a committee. Removing the dependency on a central authority for authorization reduces the risk of misconduct and misuse as auditing and strong data governance policies are baked into the approach [60, 157].

##### Decentralized identity

Self-sovereign identity (SSI) [159] is a conceptual model that emphasizes individuals or entity control over their digital identities, advocating for sole ownership and management. SSI facilitates a decentralized *IAM* system, where users authenticate and assert their access rights using verifiable credentials (VCs). VCs are digital credentials that are tamper-evident and can be verified cryptographically. This enables user identity verification while only sharing relevant information for a given context, which can enhance long-term privacy [160].

#### Immutable ledger technology

Distributed ledger technology (DLT), such as blockchain, can be used to deliver the *information management component* of consent management system [161, 162]. DLT systems grant access to the genomic data if the data request matches the consent and complies with GDPR’s right to be forgotten, by detaching the ledger-based consent object from the genomic data that are stored elsewhere. It should be noted that while no identifiable information is publicly accessible, the activity of granting and revoking consent is recorded and might still reveal compromising information. The need for anonymity hence needs to be carefully balanced against the benefits from provenance of the process. Two DLT solutions for dynamic consent have been proposed, DWARNA [163] and ConsentChain [164].

DWARNA stores participants’ consent in a permissioned blockchain network implemented using the stand-alone instance of Hyperledger Fabric implementation [165]. However, DWARNA is limited in treating consent state as a binary variable (broad yes/no) and, therefore, does not allow granular control over data use based on ontology-based encoding of genomic data.

ConsentChain is another proof-of-concept blockchain-based solution for managing informed consent in clinical trials. It offers more granularity compared to DWARNA by converting consent preferences into machine-readable codes using ontologies. However, ConsentChain relies on the Ethereum platform, which suffers from scalability and performance issues due to high transaction costs and low throughput. In contrast, DWARNA is built on a private blockchain and does not incur any fees for adding consent data.

##### Current barriers for DLT

DLT offers a secure, immutable, auditable, and transparent record of activities [166] where any modifications, such as changes to consent, are applied through a preagreed programmatic process rather than the approval by a central authority. However, current user-friendliness and low awareness among research and practitioners [167] hamper proof-of-principle applications. DLT systems are often more complex and less intuitive than centralized or federated systems and require a higher level of technical expertise and communication. The lack of compatibility between different DLT systems [168] adds to the fragmentation of the space. The Hyperledger project seeks to overcome these challenges by providing several DLT frameworks that can be customized and integrated for various organizational needs, including health care and genomics research programs [59, 169, 167].

Current proof-of-concept dynamic consent platforms lack interoperability with both the health care and research systems. Embedding interoperability based on data standards such as the HL7 Fast Healthcare Interoperability Resources (FHIR) and other interchangeable standards [170, 171] in a dynamic consent platform (i) helps standardize data to increase connectivity with accredited laboratory, health records, and research systems; (ii) makes data accessible for participants who want to access their own data or the results of research they are involved in; and (iii) ensures compliance with the rigorous regulatory standards (like those set by the US Food and Drug Administration or the European Medicines Agency) for submitting data or results, especially for clinical trials.

#### Personal data server

Offering a completely autarkic data management approach, personal data servers [172], such as SOLID PODS (Social Linked Data Personal Online Data Stores) [173, 174], offer a decentralized and secure way for individuals to manage their own data, including genomic data, with control over access permissions. Individuals can choose to either establish their own server or opt to use a PODS provider like Inrupt PODS [175], providing advantages such as enhanced privacy, consent management, and better interoperability across different applications and services. However, the benefits of absolute data control in personal servers can be overshadowed by potential shortcomings, including availability issues, data corruption challenges, and the lack of guaranteed provenance, which can pose risks, especially in clinical decision-making.

## Suggested Framework

**Figure 1:**
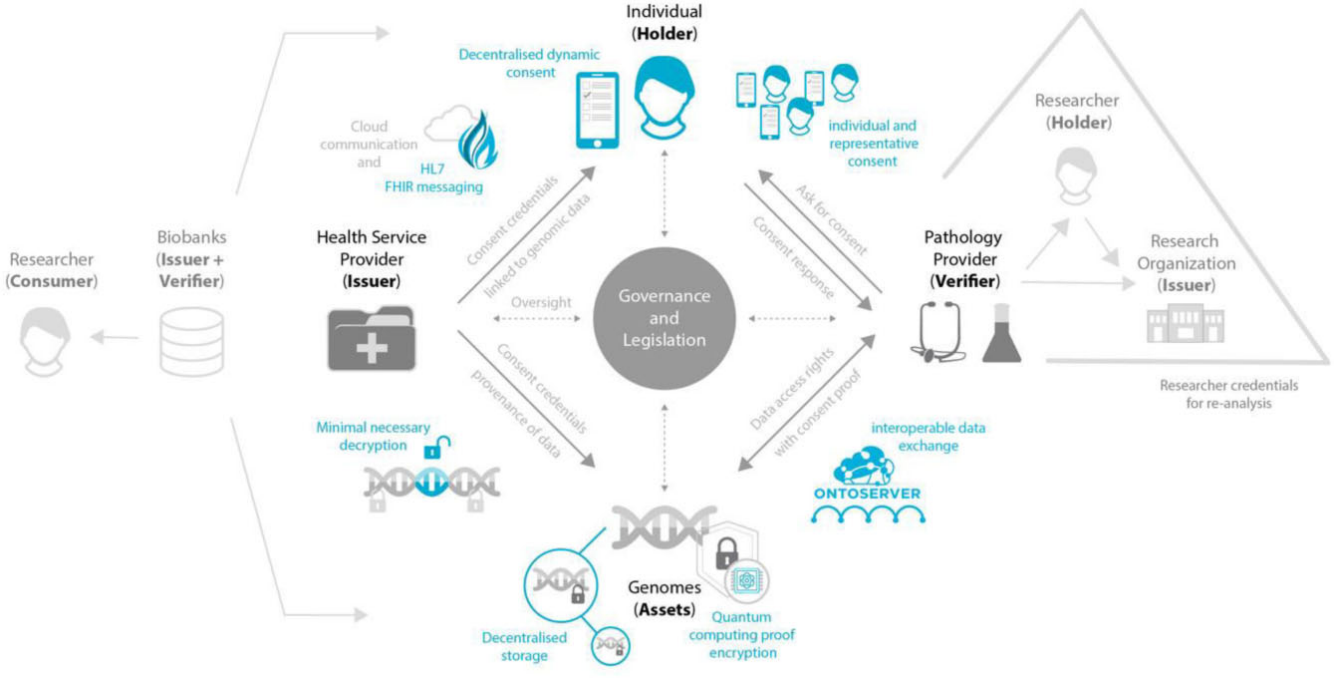
A trust model using the self-sovereign identity framework to enable participant-controlled consent management in genomics.

We envision a system that, while subject to governance and law, puts the individual at the top of the decision-making process (Fig. 1). We discuss 3 scenarios of genomic data handling: (i) health care, (ii) reanalysis of health care created data, and (iii) analysis of data created for research purposes (e.g., biobanks). We also suggest where various technologies could be used in the systems such FHIR, Ontoserver, future-proof data encryption algorithms, and decentralized data storage.

**In the health care context**, the clinician tasks the health service provider (the issuer) to initiate a genomic test after obtaining the patient’s consent, using an FHIR-based system that ensures traceability of the order within the health system. The system generates a VC of the patient (the holder). This *patient* VC contains crucial patient information, including the patient’s identity, consent signature, and usage scope. This VC is then verified by the pathology provider (the verifier) to create the genomic data (the asset), whose lab-signed provenance is then attached to the VC.

As data custodians, the pathology provider encrypts the data with future-proof encryption algorithms, such as postquantum cryptographic methods that allows only the needed genomic loci be decrypted and only when requested by the patient. The data are then stored across decentralized nodes, further increasing obfuscation and reducing the reliance on a single data provider.

The health care provider subsequently verifies the information within the received VC along with the pathology report. After verification, they send the VC to the patient, who stores it securely in their personal digital wallet as proof of their genome asset and pathology report, as well as facilitating sharing and verification with other parties.

**In the genetic reanalysis scenario**, either the health care provider or the researcher requests patient consent, which is verified by the pathology provider to enable data access. Researchers first need to prove their bona fide status as a researcher through a *researcher* VC (Fig. 1, e.g., with research organization as the Issuer). The same holds true for pathology labs requesting access to data generated by a different lab for reanalysis; they need a VC from professional bodies that govern and attest to their validity. The decentralized dynamic consent platform enables patients to track the consents they have given for reanalysis and monitor how and where their genomic data are being used.

**In the context of biobanks**, they hold the role of both issuer and verifier as they initiate the data creation and coordinate the data dissemination. Researcher and other data consumers gain access after their credentials are verified (e.g., through a GA4GH Passport system) and data use terms match the participants’ consent. Again, the dynamic consent platform allows the participants to stay up to date with results and use.

Irrespective of the technology used, the sociocultural angle of genomic data management must be considered as it encompasses the public’s attitudes and perspective toward genomic health care and research [176–178]. This social license impacts individuals’ decision to receive a genomic test and participate in research. This impacts the representation of diverse populations, including Indigenous populations, in genomic datasets, which in turn define the quality of care we can deliver to distinct populations [179].

For example, Indigenous communities often have unique cultural relationships with their genetic heritage, which can differ from the Western understanding of genetic information. As dynamic consent emphasizes ongoing and flexible participation in decision-making processes, processes must account for Indigenous people’s cultural values, beliefs, and protocols. Respect for Indigenous sovereignty, self-determination, and the right to control their own genetic data is crucial when developing dynamic consent frameworks. For these reasons, genetic research with Indigenous people must involve genuine consultation, inclusive participation, and informed consent processes that are culturally appropriate and respectful of Indigenous knowledge systems. Moreover, ongoing dialogue and reciprocal partnerships are essential to ensure that the benefits of genetic research are shared equitably and that potential harms, such as the unauthorized use of genetic data or exploitation, are prevented. Only by addressing these issues can dynamic consent contribute to empowering Indigenous communities to manage and protect their genetic information, foster trust, and promote ethical genomics research.

Similarly, values, beliefs, and protocols need to be respected when it comes to professional communities. For example, while clinicians support patients controlling their data, they are concerned about patients “owning” data [15], likely due to these creating difficulties around data provenance and the ramifications of using compromised data for clinical decisions. Thus, any developed framework must keep all involved stakeholders in mind and allocate resources for appropriate communication. Specifically, the risk and benefits of genomics research, the positive impacts of data sharing, and their strong commitment and capability in protecting genomic data must be communicated to the public through educational events, online platforms, and media/social media engagement [180–184].

Finally, the legislative system needs to protect individuals against genetic discrimination and current regulatory frameworks need to evaluate decentralized and self-sovereign identity solutions. Transition toward such systems requires infrastructure remodeling, training, and education, as well as updating existing regulations. This is a resource-intensive (human and financial) process, which demands a great level of political commitment.

## Conclusion

As genomic sequencing becomes cheaper and more ubiquitous, health and research organizations need to be empowered to access global data assets that are interoperable and scale easily with the application opportunities. This needs to be underpinned by the ethical and trustworthy management of genomic data [185] as the security and privacy must be balanced with the need for clinical efficiency, as well as ethical and safe research into population-specific care improvements.

However, this balance between protection and utility varies from circumstance to circumstance. It is hence crucial to enable individuals whose genomic data are handled to engage with the process through appropriate consent models and data governance systems. Current centralized data management strategies might get overburdened by scaling up to the level of audit trails or proof of “good processes” required to build trust with participants. Emerging decentralized data and dynamic consent management approaches have sovereignty and self-determination natively enshrined into their approaches. This enables the right to control their own data and use culturally appropriate decision-making models that future participants of genomic data exchanges require.

## Abbreviations

AAI: Authentication & Authorization Infrastructure; AES: Advanced Encryption Standard; AIC: availability, integrity, and confidentiality; DAC: data access committee; DLT: distributed ledger technology; DP: differential privacy; ECC: elliptic curve cryptography; FHIR: Fast Healthcare Interoperability Resources; FL: federated learning; GA4GH: Global Alliance for Genomics and Health; GWAS: genome-wide association studies; HE: homomorphic encryption; HIPAA: Health Insurance Portability and Accountability Act; IAM: identity and access management; IPFS: InterPlanetary File System; MPC: multiparty computation; P2P: peer-to-peer; RBM: restricted Boltzmann machine; RSA: Rivest–Shamir–Adleman; SSI: self-sovereign identity; VC: verifiable credential; ZKP: zero-knowledge proof.

## Supplementary Material

giae021_GIGA_D_23_00243_Original_Submission

giae021_GIGA_D_23_00243_Revision_1

giae021_GIGA_D_23_00243_Revision_2

giae021_Response_to_Reviewer_Comments_Original_Submission

giae021_Response_to_Reviewer_Comments_Revision_1

giae021_Reviewer_1_Report_Original_Submission Bastian Greshake Tzovaras, Ph.D -- 9/28/2023

giae021_Reviewer_1_Report_Revision_1 Bastian Greshake Tzovaras, Ph.D -- 1/17/2024

giae021_Reviewer_2_Report_Original_Submission Arianna Schuler Scott --10/20/2023

giae021_Reviewer_2_Report_Revision_1 Arianna Schuler Scott -- 2/5/2024

giae021_Reviewer_2_Report_Revision_2 Arianna Schuler Scott -- 4/9/2024
