# Supplementary material for: Future-proofing genomic data and consent management: a comprehensive review of technology innovations
Source: Gigascience. 2024 Jun 5;13:giae021. doi: 10.1093/gigascience/giae021 (PMC11152178; doi:10.1093/gigascience/giae021)
Supplement: giae021_GIGA_D_23_00243_Original_Submission [file giae021_giga_d_23_00243_original_submission.pdf]

# GigaScience

## Future-proofing genomic data and consent management: a comprehensive review of technology innovations

--Manuscript Draft--

| | |
|---|---|
| **Manuscript Number:** | GIGA-D-23-00243 |
| **Full Title:** | Future-proofing genomic data and consent management: a comprehensive review of technology innovations |
| **Article Type:** | Review |
| **Funding Information:** | |
| **Abstract:** | Genomic information is increasingly used to inform medical treatments and manage future disease risks. However, any personal and societal gains must be carefully balanced against the risk to individuals contributing their genomic data. Expanding our understanding of actionable genomic insights requires researchers to access global datasets of appropriate size to capture the complexity of human diseases and population variability. Similarly, clinicians need efficient access to a patient's genome as well as representative historical records for evidence-based decisions. To improve the standard of care and reduce current health disparities, both researchers and clinicians depend on increased participation to genomic studies, especially from underrepresented populations. This requires genomic information management approaches to increase trust and ensuring ethical and culturally appropriate use of an individual's data. |
| **Corresponding Author:** | Denis Bauer<br><br>AUSTRALIA |
| **Corresponding Author Secondary Information:** | |
| **Corresponding Author's Institution:** | |
| **Corresponding Author's Secondary Institution:** | |
| **First Author:** | Adrien Oliva |
| **First Author Secondary Information:** | |
| **Order of Authors:** | Adrien Oliva<br>Anubhav Kaphle<br>Roc Reguant<br>Letitia M.F. Sng<br>Yuwan Malakar<br>Marcel Keller<br>Thilina Ranbaduge<br>Eva K F Chan<br>James Breen<br>Sam Buckberry<br>Boris Guennewig<br>Alexander Senf<br>Matilda Haas<br>Alex Brown<br>Mark J Cowley |

Powered by Editorial Manager® and ProduXion Manager® from Aries Systems Corporation

| | |
|---|---|
| | Natalie Thorne |
| | Yatish Jain |
| | Denis Bauer |
| Order of Authors Secondary Information: | |
| Additional Information: | |
| Question | Response |
| Are you submitting this manuscript to a special series or article collection? | No |
| **Experimental design and statistics**<br><br>Full details of the experimental design and statistical methods used should be given in the Methods section, as detailed in our Minimum Standards Reporting Checklist. Information essential to interpreting the data presented should be made available in the figure legends.<br><br>Have you included all the information requested in your manuscript? | Yes |
| **Resources**<br><br>A description of all resources used, including antibodies, cell lines, animals and software tools, with enough information to allow them to be uniquely identified, should be included in the Methods section. Authors are strongly encouraged to cite Research Resource Identifiers (RRIDs) for antibodies, model organisms and tools, where possible.<br><br>Have you included the information requested as detailed in our Minimum Standards Reporting Checklist? | Yes |
| **Availability of data and materials**<br><br>All datasets and code on which the conclusions of the paper rely must be either included in your submission or deposited in publicly available repositories (where available and ethically | Yes |

| | |
|---|---|
| appropriate), referencing such data using a unique identifier in the references and in the “Availability of Data and Materials” section of your manuscript.<br><br>Have you have met the above requirement as detailed in our Minimum Standards Reporting Checklist? | |

Manuscript

Click here to access/download;Manuscript;Dynamic consent review paper.docx

# Future-proofing genomic data and consent management: a comprehensive review of technology innovations

Adrien Oliva[1*], Anubhav Kaphle[1*], Roc Reguant [1], Letitia M.F. Sng[1], Yuwan Malakar[2], Marcel Keller[3], Thilina Ranbaduge[4], Eva K F Chan[5], James Breen[6,7], Sam Buckberry[6,7], Boris Guennewig[8], Alexander Senf[9], Matilda Haas[10,11], Alex Brown[6,7], Mark J Cowley[10,11], Natalie Thorne[12,13,14,15], Yatish Jain[1,116], Denis C. Bauer[1,16,17]

[1] Australian e-Health Research Centre, Commonwealth Scientific and Industrial Research Organisation, Westmead, Australia

[2] Responsible Innovation Future Science Platform, Commonwealth Scientific and Industrial Research Organisation, Brisbane, Australia

[3] Data61, Commonwealth Scientific and Industrial Research Organisation, Black Mountain, Canberra, Australia

[4] Data61, Commonwealth Scientific and Industrial Research Organisation, Eveleigh, Australia

[5] NSW Health Pathology, Sydney, New South Wales, Australia.

[6] Telethon Kids Institute, Perth, WA 6009, Australia.

[7] National Centre for Indigenous Genomics, The John Curtin School of Medical Research, Australian National University, Canberra, ACT 2601, Australia.

[8] Sydney Medical School, Brain and Mind Centre, The University of Sydney, Sydney, NSW, Australia.

[9] European Bioinformatics Institute, Wellcome Genome Campus, Hinxton CB10 1SD, UK.

[10]Children's Cancer Institute, Lowy Cancer Research Centre, UNSW Sydney, Sydney, NSW, Australia.

[11]School of Clinical Medicine, UNSW Medicine & Health, UNSW Sydney, Sydney, NSW, Australia

[12] Murdoch Children's Research Institute, Parkville, Australia 3052

[13] University of Melbourne, Melbourne, VIC, Australia

[14] Melbourne Genomics Health Alliance, Melbourne, VIC, Australia

[15] Walter and Eliza Hall Institute, Melbourne, VIC, Australia

[16] Macquarie University, Applied BioSciences, Faculty of Science and Engineering, Macquarie Park, Australia

[17] Macquarie University, Department of Biomedical Sciences, Macquarie Park, Australia

*Co-first authors

Genomic information is increasingly used to inform medical treatments and manage future disease risks. However, any personal and societal gains must be carefully balanced against the risk to individuals contributing their genomic data. Expanding our understanding of actionable genomic insights requires researchers to access global datasets of appropriate size to capture the complexity of human diseases and population variability. Similarly, clinicians need efficient access to a patient's genome as well as representative historical records for evidence-based decisions. To improve the standard of care and reduce current health disparities, both researchers and clinicians depend on increased participation to genomic studies, especially from underrepresented populations. This requires genomic information management approaches to increase trust and ensuring ethical and culturally appropriate use of an individual's data.

This review explores technical solutions for effective genomic information management, including storage, encryption, consent, and user management that go

some way to address these concerns. Recent innovations in cloud computing, quantum-computing-proof encryption, and self-sovereign identity can complement genome specific data security approaches like GA4GH Passports and the Crypt4GH file container standard in further reducing risks. However, improvements are needed in the resilience of decentralised storage and data exchange platforms as well as how the digital consenting process can adhere to culturally accepted processes.
When addressing these shortcomings, the individual and their right for self-determination needs to be put at the centre of the framework, as only on an individual level can the risk of exposing private information be accurately balanced against the received benefits.

**Keywords** Genome data privacy, Trust model, Decentralised systems, Self-sovereign identity, Dynamic Consent

## Table of Contents

**Future-proofing genomic data and consent management: a comprehensive review of technology innovations** ........ **1**

**Introduction** ........ **3**

**Genomic Data Storage Solutions** ........ **5**

*On-Premises Storage* ........ *6*

*Cloud Storage* ........ *6*

*Hybrid Storage* ........ *7*

*Decentralised Storage* ........ *8*

**Genomic Data Privacy and Security** ........ **9**

*Availability* ........ *9*

*Integrity and Privacy-preserving Techniques* ........ *10*

Federated learning (FL) ........ 11

Privacy-preserving synthetic genomic data ........ 12

*Confidentiality and cryptography methods* ........ *12*

Encryption ........ 13

Multi-party computation (MPC) ........ 13

Post-quantum cryptography (PQC) ........ 15

**Informed Consent Management** ........ **15**

*Consent Models* ........ *16*

Broad Consent ........ 16

Tiered Consent ........ 16

Dynamic Consent ........ 16

*Digital Systems for Consent Management* ........ *17*

*Decentralised approaches* ........ *18*
Decentralised identity ........ 18
Immutable ledger technology ........ 18
Personal data server ........ 20
**Conclusion ........ 20**
*Acknowledgement* ........ *23*
*Conflict of Interest* ........ *23*
*References* ........ *23*

# Introduction

Over 60 million individuals are estimated to have their genomes sequenced in a healthcare context by 2025[1]. This increase can be attributed to the decreasing cost of genome sequencing [2–5], the rise of direct-to-consumer (DTC) genetic testing companies[6], the integration of genome testing into public healthcare systems, and the launch of large-scale population genomics initiatives in numerous countries [7–11]. However, digital infrastructure, software solutions, data security measures, and legal frameworks for managing big genomic data have not kept pace with these rapid advancements. Notably lacking are advancements in ethical data management, efficient data sharing, and data sovereignty [12]. Addressing these aspects is crucial to ensure the continued participation of a privacy-aware public, especially from marginalised groups, in contributing their private information to research [13,14].

The challenges become even more pronounced when applying genomics in a clinical context, where the generated data directly impacts patient care while aiming to broaden scientific knowledge in parallel. With rapidly evolving knowledge, secure and authorised access to historical data is required to enable re-analysis. In the healthcare context, established clinical governance and security standards are applicable for managing storage and re-analysis of genomic data for clinical care. However, accessing clinically generated data for research requires systems controls and data governance that respects patient's privacy and consent rights.

One of the key concerns in genomic data management is the potential for privacy attacks that exploit an individual's sensitive health and ancestry information, particularly when clinically generated genomic data is reused for research purposes. For instance, *identification attacks,* can link an individual's genomic data with publicly available information, such as demographic data or family history to triangulate on target individuals[15,16] and current research practices of obfuscating personally identifying variants, such as rare single nucleotide polymorphism (SNPs) or germline

variants, are not sufficiently protective[17]. Even if the genomic sequence is not ascertained directly, in a *membership inference attack* the adversary can infer the membership status of individuals in genomic research studies, such as rare disease genome-wide association studies (GWAS), by leveraging allele frequencies from public databases [18,19]. This risk is exacerbated with the increasing number of large-scale national or regional studies that recruit all participants that meet broad eligibility criteria. This also extends to an individual's physical traits, demographic information, and disease susceptibility, which can be obtained through *phenotype inference attacks* using genomic data[20,21]. In these studies, whole genomic sequencing and detailed phenotyping were used to predict biometric traits including voice, biological age, and 3D facial structure[22].

Other than safeguarding the sensitive data, there are more dimensions to effective genomic data management (Figure 1). Furthermore, to generate scientific outcomes that are robust, clinically meaningful, ethical, and equitable, genomic data needs to have ethnically diverse representation[23].

However, to achieve this diversity, it is essential to acknowledge that the socio-cultural context of genomic data management extends beyond individual perspectives and involves collective experiences and histories that can shape attitudes towards genomic data sharing. This is especially pertinent for historical instances of discrimination, trauma, racism, stigma, and marginalisation[24]. These collective experiences and cultural connotations significantly influence an individual's or population's perception of the risks associated with genomic data management, creating an intricate landscape that navigates the potential misuse of this data against the interests of certain groups or populations.

Enabling a safe way forward, legislation will have to provide active governance and enforce ethical genomic data usage. For example, participants' right-to-be-forgotten, as required by data regulations such as the GDPR [23], HIPAA [25], and several others [26], can be at odds with commercial incentives. While the Australian Financial Services Council specified in their Life Code in July 2023 that genetic results cannot be used in underwriting certain life insurance policies [27], this is not the case in other countries or sectors, leaving individuals vulnerable to disadvantages from intended or incidental findings of genetic testing.

Given these additional complexities, a strong and trusted technological foundation for genomic data management is crucial. We review both proven and emerging solutions and concepts in this review.

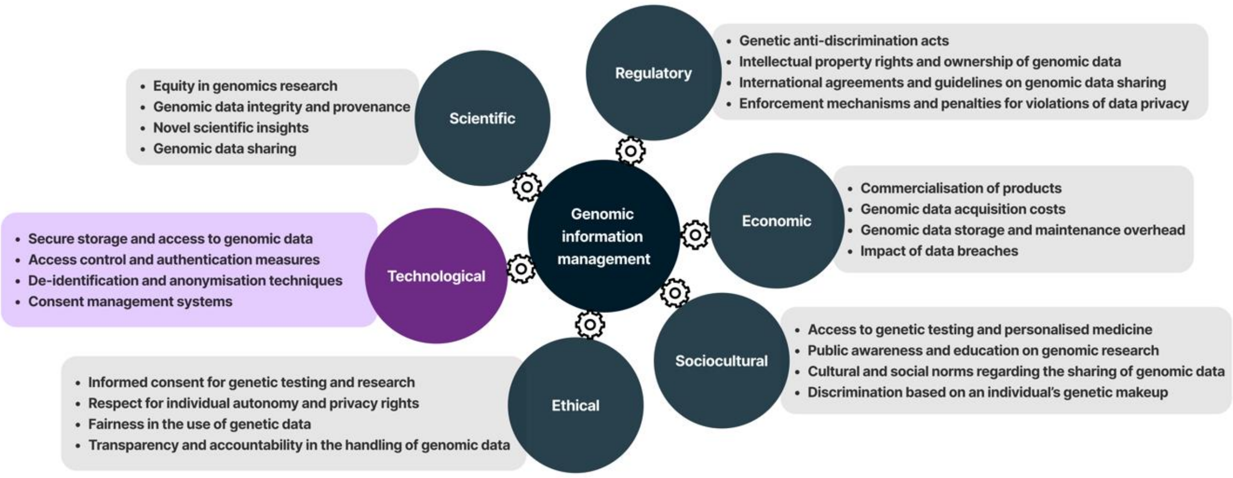


*Figure 1. The multiple dimensions of genomic information management.*

## Genomic Data Storage Solutions

Secure data storage is essential for genomic and healthcare data [28–30]. The key advantages and disadvantages of the four approaches reviewed are outlined in Table 1.

*Table 1. Different storage solutions' key advantages and disadvantages for effective genomic data management.*

| **Storage Solution** | **Advantages** | **Disadvantages** |
|---|---|---|
| On-premises | Secure (without internet) | High setup and maintenance cost including high-speed network access |
| | Full control | Low scalability |
| | Offline usage | Risk of data loss |
| | Fast data transfer | Expertise requirement |
| Cloud | High scalability | Online usage only |
| | Global connectivity | More complex cybersecurity, with higher protection if implemented correctly |
| | Expert support (including security) | Transfer cost |
| | Automatic data back ups | Trust |
| Hybrid | Staged approach | Requires more specialised expertise |

| | | |
|---|---|---|
| | On-premises and cloud benefits | Time-consuming to find best approach |
| | | Barrier to accessing clinically generated data |
| Decentralised system | No single point of failure or attack | Incentive-based approach |
| | High scalability | Incentives dependent on the market price |
| | Automatic data redundancy | Can be hampered by network congestion |
| | No third-party involvement | |

## On-Premises Storage

On-premises data management refers to storing and managing data within the physical premises of an organisation, providing complete control over data infrastructure, customising storage environment and meeting its unique needs. Storing on-premises can be highly secure when there is no external network as data access can be physically limited to only authorised personnel. This hence represents an easy option for organisations to comply with data privacy and security regulations [25,26,31]. However, this creates undesirable information silos, especially for the health care setting. Data integration and global research collaborations need carefully managed exposure to the Internet, which requires significant expertise, constant monitoring, and substantial time and resource investment to establish and oversee security protocols. In addition, on-premises data management requires an upfront investment, and replacement at relatively frequent 3–5-year intervals for the necessary infrastructure, disadvantaging smaller organisations. Ongoing costs such as energy expenses, broadband access, software licenses, certifications, IT services, broadband connectivity, and physical space to accommodate the hardware must also be considered. Furthermore, on-premises storage is not suited for short-term spikes in workload [32] as the infrastructure is static and expansion or update is expensive and time-consuming.

## Cloud Storage

Data owners can store and manage their data with a public cloud provider [33]. As organisations do not manage their infrastructure, the capital investment of on-premises management is avoided, and the ongoing cost is limited to what is consumed, shifting costs towards operational expenses. This allows organisations to pay and scale infrastructure to their changing needs, eliminating manual resource monitoring and scaling. While legislation to keep medical data inside the countries' jurisdiction has limited cloud usage in the past, cloud providers have responded by opening more in-country data centres and enabling policy configurations that ensure

data and back-up remain compliant. For example, governments use such policy-optimised clouds for their operations (e.g. AWS GovCloud and AZURE Government). Furthermore, managing data and analysis in the cloud enables seamless global collaborations and ensures reproducible results.

However, this scalability and convenience, comes with a higher security risk for the data as the uniformity of cloud account structures makes them attractive targets for hackers [34–37]. To mitigate security risks, cloud providers implement automatic counter measures and equip users with world-class security measures, including access controls through IP address restriction, continuous threat monitoring, encryption for data in transit and at rest, network and application security, data redundancy, and multi-factor authentication. While the economy of scale stems most of the costs for security, scalability and global connectedness, cloud usage can be expensive especially for egress-heavy applications, and for users that do not implement auto-archiving retention policies which take advantage of low-cost cloud storage options (like AWS Glacier).

Finally, building health care critical infrastructure on – for most – foreign national cloud provider raises concerns around sovereignty and the limits to oversight, and needs to be carefully balanced against the benefits a globally connected economic health system would provide.

A proven example*, Genomical* [38] is a cloud solution specifically designed for clinical genomic data management and, as such, is one of the first Genomic Information Management Systems (GIMS). This system takes advantage of the noted benefits of cloud storage and sharing, including security, scalability, and health system interoperability, and is underpinned by relevant federated data governance controls catering for multi-tenanted use.

## Hybrid Storage

Hybrid storage solutions can provide the best of both worlds – combining the benefits of on-premises and cloud data management solutions. To adopt such an approach, organisations require skilled workforce in both domains. By leveraging hybrid solutions, organisations can maintain a local infrastructure for sensitive data and analytics, while easily connecting storage to additional resources in the public cloud when needed, such as processing spiky analysis workloads, and genomic data processing pipelines that require different computing types. However, it comes at the expense of egress costs, potential duplication of effort for system maintenance, and limited access to clinically generated data for healthcare or research purposes. Nevertheless, it allows a staged transition to the cloud where the benefits of scalability and global connectivity can begin to be realised, without the need to move all data to the cloud at once. This enables organisations to realise economic benefits of public clouds, while maintaining sovereignty over compute and data storage capabilities.

This way, the on-premises and cloud computing infrastructure are complementary pieces of the puzzle that can help research organisations achieve their goals.

For instance, the Australian *Zero Childhood Cancer Program* [39] exemplifies this strategy. The program employs a hybrid storage setup by housing genomic data on a cloud-connected NetApp StorageGRID within a dedicated partition in an Equinix data centre. This configuration enables standardised data sharing via object store protocols, allowing integration with clouds providers, genomic analysis platforms like CAVATICA and national high performance computing resources. Through automated archiving, processed data is retained on-site, reducing long-term cloud storage costs, and data is shared with researchers through unique and secure s3 links. The program mitigates system's egress costs by routing network traffic through academic networks like AARNet where possible and leveraging Equinix Fabric for global collaborations.

## Decentralised Storage

Decentralised storage is a Web3 concept [40] where files are fragmented, encrypted, and stored over separate nodes in a decentralised or peer-to-peer (P2P) network [41]. By distributing data across different nodes, it is more secure than being stored in a single "honey pot". It also improves scalability and availability over on-premises and cloud solutions, as the P2P network can be continuously expanded with commodity hardware that is easy to onboard.

The InterPlanetary File System (IPFS) was one of the first decentralised file storage systems developed and originally used as the storage layer for blockchains [42]. IPFS uses cryptographic hashes that are based on the content of the file, thereby eliminating duplicates, and ensuring data integrity. IPFS can store and share massive amounts of data in a decentralised and economical manner which is crucial for genomic projects [43–45]. However, unlike traditional data centres, decentralised file systems are not funded through a single entity. While more traditional blockchain approaches have built-in incentive structure [46] , IPFS relies on good-will from the P2P Network notes and is, therefore, not suitable for operating critical infrastructure, such as health care. FileCoin [47] is a separate and independent decentralised protocol built on IPFS that is incentivised to offer their storage space by receiving digital currencies as a reward. Various protocols ensure the integrity, security, availability and accessibility of the data stored on the network.

While these incentives are aimed to ensure quality and make storage sustainable, it is crucial to acknowledge the potential risks associated with this approach. Fraudulent projects and initial coin offerings (ICOs) have exploited users in the past by marketing themselves as investment options. The risk of being used as a speculative commodity poses a significant risk to the stability of decentralised services, as the digital currencies market value can undermine the platform's incentive structure and functioning. Specifically, if the value of a coin declines, the motivation for nodes to continue storing data ceases, resulting in the loss of irreplaceable medical information. Given these observations, it is crucial to have a careful technical, economic, and ethical evaluation of those systems, especially in the health space.

# Genomic Data Privacy and Security

Irrespective of whether data is stored locally, in the cloud, or on decentralised systems, there is the need for robust privacy-enhancing techniques to effectively manage data security. These techniques are generally based around the three principles of information security[48]: (1) Availability, (2) Integrity, and (3) Confidentiality (AIC), which we discuss below.

## Availability

Availability ensures timely and uninterrupted access to the genomic data system by authorised users only. Human genomic data is generally protected, and access is only approved if relevant requirements are met. While clinical data can be managed by clinical governance principles, the vaiability of data usage in research requires Data Access Committees (DACs) to review access requests and ensure that the intended use of the data is permitted by the provided consent. All public genomic data repositories work on this premise, including the database of genotypes and phenotypes (dbGaP), the European Genome-Phenome Archive (EGA), and the UK Biobank [49,50] .

While this protects data and participants, it is a manual process that is not easily scalable, making datasets hard to discover and limiting their use for clinical or research benefits. The Global Alliance for Genomics and Health (GA4GH) has introduced the third access tier, apart from existing open access and controlled access called "registered access" to automate consent mechanisms and address some of the problems. The registered access [51] tier, is intended to allow access to low-risk data for research use, and it requires the user to be a 'bona-fide researcher', in addition to agreeing to the terms of use for the data.

To support automation, the GA4GH has developed "Passports", which contain a user's identity and can verify researcher status[52]. Passports can also include "Visas", allowing access to specific registered-access datasets. Visas are issued if the intended use of the data complies with restrictions set out by the DAC. Uses and restrictions are based on defined terms in the GA4GH Data Use Ontology (DUO) [53], fully automating the whole process, potentially saving months between an access request and approval.

Availability can be compromised by both non-malicious factors (such as hardware failures, software downtime, and network congestion, natural disaster) and malicious attacks (such as denial of service, also known as DoS attacks) that aim to disrupt the system's functionality. Therefore, technical, and operational security measures such as redundancy, backup, load balancing, and encryption are also essential to protect the system from threats to availability.

## Integrity and Privacy-preserving Techniques

The second pillar of AIC, data integrity practices, aims to store, and handle the data to prevent accidental or unauthorised modification throughout its entire lifecycle. Genomic data can be efficiently verified and compared by matching hash values using deterministic, collision-resistant, and non-invertible [54] cryptographic hash [55] functions such as Message Digest (MD) and the Secure Hash Algorithm (SHA) [56]. This allows for the identification of any unauthorised changes or alterations to genetic sequences without the need to compare the entire sequence. Furthermore, data hash values can be cryptographically signed [57] using a private key. This digital signature can be verified using the corresponding public key, ensuring the data's authenticity, integrity, and trustworthiness.

The privacy of the genomic data can be protected through four approaches summarised in Table 2. It should be noted that this section focuses on the research setting as in healthcare patient information cannot be obfuscated.

*Table 2: Advantages and disadvantages of different privacy preservation approaches*

| **Privacy preservation methods** | **Advantages** | **Disadvantages** |
|---|---|---|
| *k*-anonymity | Simple and intuitive | Accuracy/ Information loss |
| | numerical or categorical data | Cannot fully prevent attribute disclosure or homogeneity attack. |
| Differential privacy | Quantifies privacy risks | Trade-off between utility and privacy |
| | Strong privacy guarantees | Challenging implementation |
| Federated learning | Allows collaborative analytics | Communication overhead, statistical heterogeneity, system heterogeneity |
| | Allows large-scale computing | Data leakage still possible |
| Synthetic data methods | Works just as well as real data statistically | May introduce bias/errors |
| | Provides high quality data utility and diversity | May not guarantee perfect privacy |
| | Allows cost effective and efficient algorithm evaluation | Limiting for corner cases such as rare diseases |

### *k-anonymity*

The *k*-anonymity approach works by ensuring that the quasi-identifier for each person, such as their gender, birth date, postal code, race, ethnicity, or occupation, is indistinguishable from at least k-1 individuals in the same dataset [58,59]*.* This is done by using two approaches: (1) generalisation**,** which groups individuals together with similar attributes [60] and (2) suppression**,** which removes certain information to prevent re-identification. For example, one way to suppress genomic data is to remove germline variants, which are inherited from parents and can be used to link individuals across databases. However, this may not completely eliminate the risk of re-identification, as other types of variants or genomic features may still be informative[61,62]. Despite their widespread use, these *k*-anonymity approaches are vulnerable to attackers who have background information on the dataset [63] and are limited for high dimensional genomic data [64].

### *Differential Privacy (DP)*

Differential privacy is a mathematical framework that provides formal and provable privacy protection by introducing calibrated noise to raw data or intermediate results, making it difficult for attackers to trace data records to specific individuals [65]. The amount of noise added depends on various factors including the query type, privacy budget determining the level of privacy required, and the sensitivity of the mathematical function being computed or the query output.

In genomics, DP techniques have been proposed to counteract membership inference attacks, for example by adding noise to a genomic Beacon query response, the genome data discovery tool by GA4GH [66]. The amount of noise is carefully calibrated to balance two goals: to preserve the accuracy and hence the utility of the application and to make it harder for attackers to extract the original genomic data from the query response[67]. Additionally, DP methods for GWAS have provided maximum privacy to participants while still finding meaningful disease associations [68–70]. Although promising, the added noise in DP schemes limits its application to datasets with strong, well understood signals, e.g., disease loci with strong effect size.

### Federated learning (FL)

Federated learning (FL) is a machine learning technique that enables multiple parties to jointly train an algorithm without sharing their data thereby avoiding risks to data integrity or having to negotiate data access [71] . In this approach, computations are performed locally on the data that remains within the owner's ecosystem (e.g., server nodes, jurisdictions). These locally trained parameters are then sent to a central server that aggregates the local models from all participating peers to generate a global model shared by all [72,73]. Federated learning has been used on health data [74,75] and was shown on genomics data to achieve comparable performance compared to a centralised approach for phenotype prediction on genomic data using the UK Biobank [76].

While promising, coordinating local model aggregates can be challenging, especially when training complex FL models. Issues such as, network latency, maintaining trust and incentive among participating peers, and ensuring data quality and diversity, remains unresolved. Further, FL is specifically vulnerable to data poisoning attacks, where attackers deliberately manipulate or corrupts the data, and backdoor attacks by poisoning models to output biased results [77].

### Privacy-preserving synthetic genomic data

Creating synthetic genomic data can sidestep many data privacy issues. This data has the same statistical properties as the original dataset, but without passing on the real genomes. Several methods have been developed to generate synthetic genomes leveraging various sources of knowledge including haplotype information [78,79], demographic information and recombination inferences [80]. More advanced methods like deep neural network-based methods, such as generative adversarial networks (GANs) and restricted Boltzmann machines (RBMs) have also successfully generated synthetic genomic data where population structure and variant frequency-based features were preserved [81]. Generative methods can be utilised to create datasets that act as proxies for under-represented populations, going some way to address the known Eurocentric bias in genomic studies [82]. However, the utility of synthetic genomes is limited to evaluating algorithms, rather than for discovery projects, because they do not have more information than the original data, which is further limited by the fidelity of the generative model used.

A recent study by Oprisanu *et al.* [83] compared the above synthetic data methods for utility and privacy. They showed that recombination-based methods have high utility but low privacy, while RBMs offer a trade-off. It is worth noting that generating distributions close to the real data often generates target data points that are vulnerable to membership inference[84]. Therefore, some data integrity and security practices must be enforced even for synthetic data.

## Confidentiality and cryptography methods

Confidentiality represents the third pillar of AIC, which prevents unauthorised access or disclosure of data. We review the five most relevant and noteworthy techniques as summarised in Table 3.

*Table 3 Advantages and disadvantages of different encryption approaches*

| **Encryption solutions** | **Advantages** | **Disadvantages** |
|---|---|---|

| | | |
|---|---|---|
| Symmetric encryption | Fast and efficient | Less versatile |
| Asymmetric encryption | Public key can be shared with collaborators without compromising data integrity | Slow for large data |
| Multi-party computation | Highest security as data always stays encrypted | Computationally expensive |
| Homomorphic Encryption | Allows computations on encrypted data, without decrypting it | Computationally intensive and slow |
| Post-Quantum | Resistant to Quantum computer attacks | Difficult to scale |

## Encryption

Encryption is a cryptographic method that aims to secure genomic files by converting *plain text* to *cipher text* using different algebraic operations.

Symmetric encryption methods encrypt data using either stream ciphers such as Salsa20, CHACHA20, and AES-CTR [85] or block ciphers such as the Advanced Encryption Standard (AES) [86] and are a popular method for securing genomic data as they are fast and efficient [87]. For large genomic data, CHACHA20 is the fastest and most efficient algorithm [88], and is often used in combination with POLY1305, a message authentication code, to ensure message integrity and authentication [89]. For example, CHACHA20-POLY1305 is used in Crypt4GH [90] , a file container standard proposed by GA4GH. Its user-specific envelop encryption scheme enables random byte-level access to encrypted file content without decrypting the whole file. Block ciphers have also been used in genomic data encryption [91] .

Asymmetric or public cryptography schemes, such as Rivest-Shamir-Adleman (RSA) and elliptic curve cryptography (ECC), use two keys for encryption: a public key for encryption and a private key for decryption. The RSA algorithm has been used for genomic data to mask individual's alleles and secure cloud-based genetic paternity test results [63,92], however, it can be slow and impractical for large WGS files [92] and is therefore limited to smaller genomic files and sensitive metadata. ECC is often preferred over RSA for smaller genomic files due to its smaller key sizes and lower computational cost [93,94].

## Multi-party computation (MPC)

Multiparty computation improves upon the traditional route of encrypting data solely for storage and transport purposes, which subsequently requires decryption and

handling in an unencrypted manner during analysis. MPC frameworks allow researchers to collaborate on data analysis while maintaining privacy using cryptographic methods. While still an emerging technology, it has made rapid progress over the last few years with open-source frameworks such as MP-SPDZ [95] , which combines several MPC variants with an accessible Python user geared toward analytics. Here, we review (1) secret sharing, (2) garbled circuit method, (3) homomorphic encryption and (4) zero-knowledge proof.

### *Secret sharing*

A core technology to most MPC protocols is secret sharing, which denotes the distributed storage of information such that the parties together can reconstruct the information, but an individual party (or a small set) cannot. In some settings, secret-sharing techniques alone can be used for privacy-preserving analytics without the more expensive techniques below. These protocols have been found relatively efficient, enabling even the training of smaller deep learning models.

### *Garbled circuit method*

A popular method of developing MPC is the garbled circuit method where the function is transformed into a Boolean circuit of logic gates and encrypted to produce garbled output values [96]. Collaborators can then use garbled values and their input to generate an output, ensuring privacy and correctness. In the genomic space, the garbled circuit solution has been used for secure genomic data analysis [97,98] and diagnosis [99], but this approach can be expensive for large genomic data.

### *Homomorphic encryption*

Homomorphic encryption (HE) is a cryptographic technique that allows computations to be performed on encrypted data without decrypting them first. This means that the data can be processed securely without revealing any sensitive information to the parties involved. This can be useful for genome queries and statistical analyses such as GWAS, which aim to find genetic variants associated with certain traits or diseases. Several methods have been developed for HE-based genome queries and statistical analyses [100–103]. However, HE requires more computational resources and time than regular encryption methods, however, new algorithms and techniques have been proposed to improve performance [104]. Combining HE with MPC is argued to further reduce overhead compared to traditional encryption methods. It has shown potential for encrypted control over genomic data to enhance trust within genomics research programs. Additionally, techniques that combine HE with differential privacy can be employed to ensure the security of genomic data and enable privacy while sharing summary results[105] .

### *Zero-knowledge proof*

Zero-knowledge proof (ZKP) is a cryptographic method allowing one party to prove a claim's correctness to another party without revealing additional information [106]. In genomics, ZKP has been used to enable secure genomic query [107], and sequence similarity search [108]. ZKP methods can be hard to set-up, requiring subject matter expertise and they are not scalable due to high compute power on large dataset.

### Post-quantum cryptography (PQC)

There is growing concern within the community of information security experts that most of the currently available cryptographic methods, such as RSA and ECC, are vulnerable to attacks by quantum computers (e.g., Shor's algorithm[109]). Although quantum computers are expensive and still developing, it is feared that attackers are already downloading sensitive data to decrypt in the future when quantum computers become more readily available[110]. This has significant implications for genomic data encrypted by today's cryptographic methods as genomic data retains its relevancy over an individual's lifespan and even that of their direct descendants.
In response, the National Institute of Standards and Technology (NIST) in the United States have announced encryption algorithms that were designed to resist attacks from classical and quantum computers thereby enabling post-quantum migration of cryptosystems. These include lattice-based algorithms CRYSTALS-Kyber[111] for general purpose encryption, CRYSTALS-Dilithium[112], and FALCON[113], both for digital signatures. Using such PQC methods for genomic data now is a proactive step to ensuring that genomic data is protected against possible future attacks while saving time and money rather than re-encrypting when quantum computers become more accessible.

## Informed Consent Management

The purpose of ensuring genome data security is to enable responsible usage of the data for clinical and research purposes. However, data can only be collected, stored, and shared with informed consent from patients/participants, ensuring confidentiality and agreed-upon use. Robust security measures and ethical informed consent are essential for fostering trust and protecting individuals' privacy.

Informed and ongoing consent management is a required ethical process of obtaining and managing consent from individuals for collecting, storing, and using their genomic information[114–117] both within healthcare and research settings. Different regions and countries may have specific legal frameworks and guidelines pertaining to informed consent for genomic research. These varying regulations and requirements must be considered and adhered to when designing and conducting studies involving genomic data. Another important consideration is ensuring that existing healthcare systems of record (e.g. My Health Record) can hold consent information so clinically generated data can be re-used for research.

Genomic research studies employ various consent models based on the study size, application area, and research direction. We will focus on three consent models: (1) broad, (2) tiered, and (3) dynamic consent, all of which aim to balance the participant's control over their data with efficient data sharing[118].

## Consent Models

### Broad Consent

The *broad* consent model is used for studies where the genomic data collected may additionally be used in other research unrelated to the original study for which the consent was given[119,120]. This model is commonly used in large longitudinal biobanks due to its ease. However, it poses ethical and legal challenges as individuals may not fully understand what all they are consenting to. This is partly due to the broad language used during consent process to cover future studies that have not yet been defined.

### Tiered Consent

Unlike the broad consent model, the tiered consent model provides participants with granular control over their data by offering consent options based on the nature of the research[121] . For example, participants can choose to share specific genomic information or participate in specific research studies only. However, the tiered consent model creates administrative and logistical challenges for researchers to complying to the different levels of consents, and for participants to inform themselves up-front about the different options[118].

### Dynamic Consent

The dynamic consent model is a broader concept that aims to facilitate continuous engagement of participants using personalised online consent processes and digital communication platforms[122–125]. The dynamic consent concept can also facilitate other consent models, such as Dynamic Specific consent and Meta consent. *Dynamic specific consent* tailors information specifically to participants' preferences and needs, allowing them to choose the level of detail and complexity they want to receive, and ensuring that they are not overwhelmed or confused by information that may not be relevant or comprehensible to them[118]. In *Meta consent*, instead on deciding their contribution to individual studies, participants define the consent parameters that then automatically approve or reject future studies. For example, tiered, broad consent may be further stratified by purpose of use (academic, commercial), data type (genomic data, medical records, imaging), research institution (universities, research labs), or funding sources (public or private)[126]. This offers fine-grained control over the use of their data without the overhead of managing individual study requests. However, it is difficult to get right: Meta consent models, often require participants to make an upfront decision for future research at a high level of abstraction, without the concrete research context or awareness of what the specific benefits or risks might be [118].

## Digital Systems for Consent Management

Traditionally, consent was obtained and recorded as a paper-based documentation. However, with tiered and dynamic consent, digital systems with electronic consent forms have become more common[127]. A digital consent management system allows patients/participants to enter, manage, and withdraw their consent for data use through a digital interface, such as a web portal or a mobile application. It can also allow authorised researchers/clinicians to request access to the genomic and other health data located in the storage system for various purposes such as research or clinical decisions. However, depending on whether the data is stored within a health system of record or research system, different approaches and policies may apply to ensure regulatory and ethical compliance. Of the multiple components of a digital consent management system, we discuss three below: (1) identity and access management (IAM), (2) personalised consent elements, and (3) information storage in the context of genomics research programs.

### *IAM component*

The IAM component manages registration and authentication, allowing authorised users (participants/patients or researchers/clinicians) access to the system and its resources. Users are assigned an 'ID' for recognition, linking their genomic data and consent along with health records. GA4GH registered access and Passport standard can be repurposed for researchers' interaction with the digital system.

### *Personalise consent materials*

Especially useful for genomic research programs are technologies like interactive webpages and virtual or augmented reality to *personalise consent materials* for clear and engaging explanations of complex scientific concepts and of the program's research aims to participants[128]. Language aids such as chatbots[129] and translation systems are also powerful tools to supplement this component for non-English speaking participants. An ontology system can be integrated so that consent language can be transformed into machine-readable codes that tag datasets and manage data permissions[53,130]. These elements are also helpful during consent process in a healthcare setting where the data might be used for secondary analysis.

### *Information storage component*

As genomic research programs often handle health information as well, the *information storage component* can be integrated with data capture and management systems that comply with regulatory standards prescribed by sovereign privacy legislation such as GDPR, APP's and HIPAA to ensure that participants' data and consent are securely stored. For instance, CTRL[131], an Australian Genomics dynamic consent platform, integrates with REDCap[132] data capture system, a popular free regulatory-compliant data capture system, to collect and combine consent and research data. Other alternatives such as Castor EDC, Qualtrics, and ClinCapture are

available with more user-friendly interfaces and customer support.  Dynamic consent solutions for consumers are needed within the existing health care system infrastructure. Systems to enable and capture health interoperable patient consent are now available such as Personify Care and GeneticAdvisor.  Ongoing consent management in the broader context of reuse of genomic information within healthcare as well as for research is needed.

Dynamic consent in its current form has gone some way in building community trust, however there are challenges arising from relying on one central organisation to enact consent changes, such as impose a substantial burden on the resulting IT infrastructure and personnel to manually demonstrate compliance[123]. Similarly, for participants where currently engagement levels are bounded by the resource levels of the organisation. This might lead to participants missing deadlines to participate in a study they would have consented to. Additionally, participants uncomfortable with digital technologies or residing in remote areas with limited internet connectivity may face difficulties engaging with the current centrally managed implementations of dynamic consent[133].

## Decentralised approaches

Decentralised dynamic consent systems aim to overcome the limitations of centrally managed structures by delivering both the IAM and information storage components in a programmatically insured process. This allows real-time monitoring of data use, participant-executed revocation of data, and a tamper-proof record of consent changes. It also can cater for the remote or culturally appropriate collection of consent, such as the immutable collection of consent offline, or unfettered voting through a committee. Removing the dependency on a central authority for authorisation, reduces the risk of misconduct and misuse as auditing and strong data governance policies are baked into the approach.

### Decentralised identity

Self-Sovereign Identity (SSI)[134] is a framework that allows individuals to control their own digital identities. SSI can be used for decentralised *IAM* setup, where users can prove their identity and access rights using verifiable credentials (VCs). VCs are digital credentials that are tamper-evident and can be verified cryptographically. This enables user identity verification while only sharing relevant information for a given context, which can enhance long-term privacy[135].

### Immutable ledger technology

Distributed ledger technology (DLT), such as blockchain, can be used to deliver the *information management component* of consent management system[136,137]. DLT

systems grants access to the genomic data if the data request matches the consent and complies with GDPR's right-to-be-forgotten regulation, by detaching the genomic data from the blockchain. However, it should be noted that while no identifiable information is shared, the activity of granting and revoking consent is recorded and might reveal compromising information. The need for anonymity needs to be balanced against the benefits from provenance of the process. Two DLT solutions for dynamic consent have been proposed, DWARNA[138] and ConsentChain[139].

DWARNA stores participants' consent in a permissioned blockchain network implemented using the stand-alone instance of Hyperledger Fabric implementation [140]. However, DWARNA is limited in treating consent state as a binary variable (broad yes/no) and, therefore, does not allow granular control over data use based on ontology-based encoding of genomic data.

ConsentChain is another proof-of-concept blockchain-based solution for managing informed consent in clinical trials. It offers more granularity compared than DWARNA for data sharing by converting consent preferences into machine-readable codes using ontologies. However, ConsentChain has a drawback in that it relies on the Ethereum platform, which suffers from scalability and performance issues due to high transaction costs and low throughput. In contrast, DWARNA is built on a private blockchain and does not incur any fees for adding consent data.

### *Current barriers for DLT*

DLT offer a secure, immutable, auditable, and transparent record of activities[141] where any modifications made to the blockchain require consensus between peers, without the involvement of any central authority. However, there are barriers to its adoption, such as the lack of user-friendliness and low awareness among research and practitioners in the field[142]. DLT systems are often more complex and less intuitive than centralised or federated systems and require a higher level of technical expertise and trust. Another challenge is the diversity and lack of compatibility of different DLT systems[143]. A possible way to overcome this challenge is to use tools within the Hyperledger project, a collaborative effort that provides a variety of DLT frameworks that can be customised and integrated for various organisational needs, including healthcare and genomics research programs [45,144,145].

Current proof-of-concept dynamic consent solutions also lack an essential layer of interoperability. Incorporating HL7 Fast Healthcare Interoperability Resources (FHIR) standards into these de-centralised systems can greatly enhance the interconnectedness between different systems. For example, *Genomical* uses healthcare interoperability standards, including Ontoserver [146], the HL7 FHIR-based terminology service adopted by digital health agencies around the world. Embedding interoperability in a dynamic consent framework helps standardise data to increase

connectivity with different clinicians’ and researchers' systems and enables participants to engage with the outcomes at their chosen level of detail.

### Personal data server

Offering a completely autarkic data management approach, personal data servers [147], such as SOLID PODS (Social Linked Data Personal Online Data Stores) [148,149], offer a decentralised and secure way for individuals to manage their own data, including genomic data, with control over access permissions. Individuals can choose to either establish their own server or opt to use a PODS provider like Inrupt PODS), providing advantages such as enhanced privacy, consent management, and better interoperability across different applications and services. However, the benefits of absolute data control in personal servers can be overshadowed by potential shortcomings, including availability issues, data corruption challenges, and the lack of guaranteed provenance, which can pose risks, especially in clinical decision-making.

## Conclusion

As genomic sequencing becomes cheaper and more ubiquitous, it is crucial to establish an ethical and trustworthy management model for genomic data[150]. The need for security must be balanced with the requirements of clinical applications and ongoing research into new clinical interventions. Both require the efficient access to global cohorts and sophisticated implementation of clinical systems for genomics. As this balance between security and utility may vary from circumstance to circumstance, it is crucial to enable individuals whose genomic data is handled to engage with the process through appropriate research consent models and data governance systems that do not rely on a central administrative body.

We envision a system that, while subject to governance and law, has the individual at the centre of the decision-making process (Figure 2). Here, research consortia or clinicians can order a genomic test from a pathology lab (Issuer), e.g., with an HL7 or FHIR pathology order that is passed to a FHIR-enabled genomic data management digital health system such as Genomical. This creates the genome (Asset), which gets attached to the verifiable credential (VC) held by the research participant (Holder) in a digital wallet. The VC contains the participant's digital identity and consent information, detailing the use of their genomic data. The Issuer encrypts the data with a quantum computing-proof encryption algorithm that allows only the needed genomic loci be decrypted and only upon request from the participant. The data is then stored across decentralised nodes, further increasing obfuscation and reducing the reliance on a single data provider. The researcher or clinician (Verifier) requests the participant's consent to use their data through a decentralised dynamic consent system that first test the legitimacy of the Verifier’s digital identity. Either the participant itself or their

appointed representative or committee responds to the data use request. If the Verifier is granted permission, they can access the participant's standardised data, e.g., with Ontoserver, for research or clinical application.

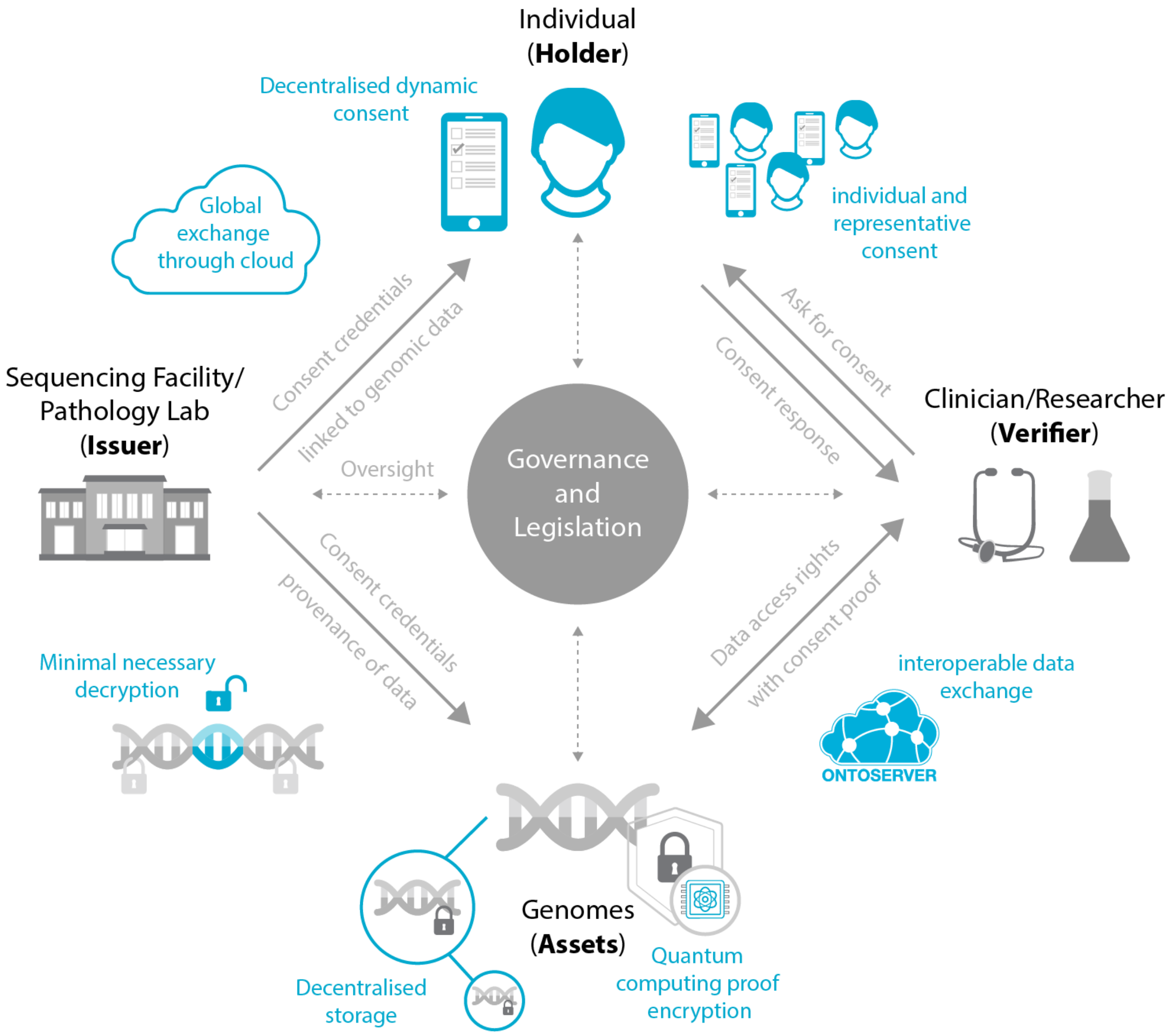


*Figure 2:* ***A trust model using the self-sovereign identity framework to enable participant-controlled consent management in genomics****.*

Irrespective of the technology used, the sociocultural angle of genomic data management must be considered as it encompasses the public's attitudes and perspective towards genomic healthcare and research[151–153]. This social license impacts individual‘s decision to receive a genomic test and also participation in research; which ultimately impacts the representation of diverse populations, including Indigenous populations, in genomic datasets that define the quality of care we can deliver to distinct populations[154].

For example, Indigenous communities often have unique cultural relationships with their genetic heritage, which can differ from the Western understanding of genetic

information. As dynamic consent emphasises ongoing and flexible participation in decision-making processes, processes must account for Indigenous people's cultural values, beliefs, and protocols. Respect for Indigenous sovereignty, self-determination, and the right to control their own genetic data is crucial when developing dynamic consent frameworks. For these reasons, genetic research with Indigenous people must involve genuine consultation, inclusive participation, and informed consent processes that are culturally appropriate and respectful of Indigenous knowledge systems. Moreover, ongoing dialogue and reciprocal partnerships are essential to ensure that the benefits of genetic research are shared equitably and that potential harms, such as the unauthorised use of genetic data or exploitation, are prevented. Only by addressing these issues can dynamic consent contribute to empowering Indigenous communities to manage and protect their genetic information, foster trust, and promote ethical genomics research.

Similarly, values, beliefs, and protocols need to be respected when it comes to professional communities. For example, while clinicians support patient controlling their data, they are concerned about patients ‘owning’ data[155], likely due to these creating difficulties around data provenance and the ramifications of using compromised data for clinical decisions. Thus, any developed framework must keep all involved stakeholders in mind and allocate resources for appropriate communication. Specifically, the risk and benefits of genomics research, the positive impacts of data sharing, and their strong commitment and capability in protecting genomic data must be communicated to the public through educational events, online platforms, and media/social media engagement [156–160].

Finally, the legislative system needs to protect individuals against genetic discrimination and current regulatory frameworks need to evaluate decentralised and self-sovereign identity solutions. Transition towards such systems requires infrastructure remodelling, training, and education, as well as updating existing regulations. This is a resource-intensive (human and financial) process, which demands a great level of political commitment.

In summary, the engagement and retention of consumers and participants are crucial for clinical genomic applications and research going forward. This can be achieved by putting people at the centre of the data management strategy. For example, by enshrining sovereignty, self-determination, and the right to control their own genetic data into the system through decentralised dynamic consent that caters for various decision-making models. Healthcare and research organisations need to be empowered with tamper-proof readily available yet protected data, by health-interoperable and distributed storage solutions, future-proof encryption and facilitating the efficient exchange of global genomic data.

## Acknowledgement

We thank Megan Prictor, John Phillips, and Jo Spencer for their critical reading of the paper. We extend our gratitude to Megan for her thought leadership that has inspired our focus areas.

## Conflict of Interest

B.G. is a director of Pacific Analytics PTY LTD & SMRTR PTY LTD, Australia; his research is supported through the MRFF (Establishing epigenetic biomarkers in Indigenous Australians for precision health) and a BISA Accelerator grant.

A.S. is a director of Pacific Analytics PTY LTD & SMRTR PTY LTD, Australia.

N.T. is the Scientific Director, Genomical, Australia.

## References

1. Birney, E., Vamathevan, J. & Goodhand, P. Genomics in healthcare: GA4GH looks to 2022. *BioRxiv* 203554 (2017).
2. Saunders, G. *et al.* Leveraging European infrastructures to access 1 million human genomes by 2022. *Nat Rev Genet* **20**, 693–701 (2019).
3. Goodwin, S., McPherson, J. D. & McCombie, W. R. Coming of age: ten years of next-generation sequencing technologies. *Nat Rev Genet* **17**, 333–351 (2016).
4. Schwarze, K., Buchanan, J., Taylor, J. C. & Wordsworth, S. Are whole-exome and whole-genome sequencing approaches cost-effective? A systematic review of the literature. *Genetics in Medicine* **20**, 1122–1130 (2018).
5. Li, H. *et al.* Cost-reduction strategies in massive genomics experiments. *Mar Life Sci Technol* **1**, 15–21 (2019).
6. Stoeklé, H.-C., Mamzer-Bruneel, M.-F., Vogt, G. & Hervé, C. 23andMe: a new two-sided data-banking market model. *BMC Med Ethics* **17**, 1–11 (2016).
7. Wong, E. *et al.* The Singapore National Precision Medicine Strategy. *Nat Genet* 1–9 (2023).
8. Kowal, E., Easteal, S. & Gooda, M. Indigenous genomics. *Australasian Science* **37**, 18–20 (2016).
9. Forsberg, J. S. & Soini, S. A big step for Finnish biobanking. *Nat Rev Genet* **15**, 6 (2014).
10. Leitsalu, L. *et al.* Cohort profile: Estonian biobank of the Estonian genome center, university of Tartu. *Int J Epidemiol* **44**, 1137–1147 (2015).
11. Bycroft, C. *et al.* The UK Biobank resource with deep phenotyping and genomic data. *Nature* **562**, 203–209 (2018).
12. Malakar, Y., Lacey, J., Twine, N. A. & Bauer, D. C. Applying a risk governance approach to examine how professionals perceive the benefits and risks of clinical genomics in Australian healthcare. *New Genet Soc* **42**, e2192472 (2023).

13. Cheung, R., Jolly, S., Vimal, M., Kim, H. L. & McGonigle, I. Who's afraid of genetic tests?: An assessment of Singapore's public attitudes and changes in attitudes after taking a genetic test. *BMC Med Ethics* **23**, 5 (2022).
14. Grishin, D. *et al.* Citizen-centered, auditable and privacy-preserving population genomics. *Nat Comput Sci* **1**, 192–198 (2021).
15. Gymrek, M., McGuire, A. L., Golan, D., Halperin, E. & Erlich, Y. Identifying personal genomes by surname inference. *Science (1979)* **339**, 321–324 (2013).
16. Erlich, Y. *et al.* Redefining genomic privacy: trust and empowerment. *PLoS Biol* **12**, e1001983 (2014).
17. Von Thenen, N., Ayday, E. & Cicek, A. E. Re-identification of individuals in genomic data-sharing beacons via allele inference. *Bioinformatics* **35**, 365–371 (2019).
18. Raisaro, J. L. *et al.* Addressing Beacon re-identification attacks: quantification and mitigation of privacy risks. *Journal of the American Medical Informatics Association* **24**, 799–805 (2017).
19. Shringarpure, S. S. & Bustamante, C. D. Privacy risks from genomic data-sharing beacons. *The American Journal of Human Genetics* **97**, 631–646 (2015).
20. Venkatesaramani, R., Malin, B. A. & Vorobeychik, Y. Re-identification of individuals in genomic datasets using public face images. *Sci Adv* **7**, eabg3296 (2021).
21. Lippert, C. *et al.* Identification of individuals by trait prediction using whole-genome sequencing data. *Proceedings of the National Academy of Sciences* **114**, 10166–10171 (2017).
22. Lippert, C. *et al.* Identification of individuals by trait prediction using whole-genome sequencing data. *Proceedings of the National Academy of Sciences* **114**, 10166–10171 (2017).
23. Lee, S. S. Excavating the personal genome: The good biocitizen in the age of precision health. *Hastings Center Report* **50**, S54–S61 (2020).
24. Bullen, J. *et al.* From Deficit to Strength-Based Aboriginal Health Research—Moving toward Flourishing. *Int J Environ Res Public Health* **20**, 5395 (2023).
25. U.S. Dept. of Health and Human Services. Standards for privacy of individually identifiable health information. *Federal Registrar2002; 45 CFR, Parts 160–4.* (2002).
26. The California Consumer Privacy Act of 2018.
27. Tiller, J. *et al.* Community concerns about genetic discrimination in life insurance persist in Australia: A survey of consumers offered genetic testing. *European Journal of Human Genetics* 1–9 (2023).
28. Seh, A. H. *et al.* Healthcare Data Breaches: Insights and Implications. *Healthcare* **8**, 133 (2020).
29. Pandey, A. K. *et al.* Key Issues in Healthcare Data Integrity: Analysis and Recommendations. *IEEE Access* **8**, 40612–40628 (2020).
30. Abinaya B. & Santhi S. A survey on genomic data by privacy-preserving techniques perspective. *Comput Biol Chem* **93**, 107538 (2021).
31. Regulation (EU) 2016/679 , General Data Protection Regulation (GDPR). *OJ L 119, 4.5.2016, p. 1–88* (2016).
32. Golec, D., Strugar, I. & Belak, D. The Benefits of Enterprise Data Warehouse Implementation in Cloud vs. On-premises. *ENTRENOVA - ENTerprise REsearch InNOVAtion* **7**, 67–76 (2021).
33. Wu, J., Ping, L., Ge, X., Wang, Y. & Fu, J. Cloud Storage as the Infrastructure of Cloud Computing. in *2010 International Conference on Intelligent Computing and Cognitive Informatics* 380–383 (IEEE, 2010). doi:10.1109/ICICCI.2010.119.
34. B. K. Reddy, B. T. Rao, Dr.L.S.S. Reddy & P. Kiran. Issues in Cloud Computing. *Global Journal of Computer Science and Technology* **11**, (2011).

35. Ghani, A., Badshah, A., Jan, S., Alshdadi, A. A. & Daud, A. Issues and challenges in Cloud Storage Architecture: A Survey. (2020).
36. Prajapati, P. & Shah, P. A Review on Secure Data Deduplication: Cloud Storage Security Issue. *Journal of King Saud University - Computer and Information Sciences* **34**, 3996–4007 (2022).
37. Odun-Ayo, I., Ajayi, O., Akanle, B. & Ahuja, R. An Overview of Data Storage in Cloud Computing. in *2017 International Conference on Next Generation Computing and Information Systems (ICNGCIS)* 29–34 (IEEE, 2017). doi:10.1109/ICNGCIS.2017.9.
38. Genomical. *https://genomical.com.au/*.
39. Wong, M. *et al.* Whole genome, transcriptome and methylome profiling enhances actionable target discovery in high-risk pediatric cancer. *Nat Med* **26**, 1742–1753 (2020).
40. Murray, A., Kim, D. & Combs, J. The promise of a decentralized internet: What is Web3 and how can firms prepare? *Bus Horiz* **66**, 191–202 (2023).
41. Sharma, P., Jindal, R. & Borah, M. D. Blockchain-based decentralized architecture for cloud storage system. *Journal of Information Security and Applications* **62**, 102970 (2021).
42. Benet, J. IPFS - Content Addressed, Versioned, P2P File System. (2014).
43. Mani, V., Manickam, P., Alotaibi, Y., Alghamdi, S. & Khalaf, O. I. Hyperledger Healthchain: Patient-Centric IPFS-Based Storage of Health Records. *Electronics (Basel)* **10**, 3003 (2021).
44. Azbeg, K., Ouchetto, O. & Jai Andaloussi, S. BlockMedCare: A healthcare system based on IoT, Blockchain and IPFS for data management security. *Egyptian Informatics Journal* **23**, 329–343 (2022).
45. Kumar, R., Marchang, N. & Tripathi, R. Distributed Off-Chain Storage of Patient Diagnostic Reports in Healthcare System Using IPFS and Blockchain. in *2020 International Conference on COMmunication Systems & NETworkS (COMSNETS)* 1–5 (IEEE, 2020). doi:10.1109/COMSNETS48256.2020.9027313.
46. Mackey, T. K. *et al.* Establishing a blockchain-enabled Indigenous data sovereignty framework for genomic data. *Cell* **185**, 2626–2631 (2022).
47. Filecoin: A Decentralized Market for Storage. *[online] Available: https://filecoin.io.*
48. Aminzade, M. Confidentiality, integrity and availability–finding a balanced IT framework. *Network Security* **2018**, 9–11 (2018).
49. Dyke, S. O. M. Genomic data access policy models. in *Responsible Genomic Data Sharing* 19–32 (Elsevier, 2020).
50. Dyke, S. O. M. *et al.* Registered access: authorizing data access. *European Journal of Human Genetics* **26**, 1721–1731 (2018).
51. Dyke, S. O. M. *et al.* Registered access: a 'Triple-A'approach. *European Journal of Human Genetics* **24**, 1676–1680 (2016).
52. Voisin, C. *et al.* GA4GH Passport standard for digital identity and access permissions. *Cell Genomics* **1**, 100030 (2021).
53. Lawson, J. *et al.* The Data Use Ontology to streamline responsible access to human biomedical datasets. *Cell Genomics* **1**, 100028 (2021).
54. Debnath, S., Chattopadhyay, A. & Dutta, S. Brief review on journey of secured hash algorithms. in *2017 4th International Conference on Opto-Electronics and Applied Optics (Optronix)* 1–5 (IEEE, 2017).
55. Preneel, B., Govaerts, R. & Vandewalle, J. Cryptographic hash functions: an overview. in *Proceedings of the 6th international computer security and virus conference (ICSVC 1993)* vol. 19 (1993).

56. Stinson, D. R. Some observations on the theory of cryptographic hash functions. *Des Codes Cryptogr* **38**, 259–277 (2006).
57. Ayday, E., Tang, Q. & Yilmaz, A. Cryptographic solutions for credibility and liability issues of genomic data. *IEEE Trans Dependable Secure Comput* **16**, 33–43 (2017).
58. Sweeney, L. k-anonymity: A model for protecting privacy. *International journal of uncertainty, fuzziness and knowledge-based systems* **10**, 557–570 (2002).
59. Rajendran, K., Jayabalan, M. & Rana, M. E. A study on k-anonymity, l-diversity, and t-closeness techniques. *IJCSNS* **17**, 172 (2017).
60. Malin, B. A. Protecting genomic sequence anonymity with generalization lattices. *Methods Inf Med* **44**, 687–692 (2005).
61. Humbert, M., Ayday, E., Hubaux, J.-P. & Telenti, A. Reconciling utility with privacy in genomics. in *Proceedings of the 13th Workshop on Privacy in the Electronic Society* 11–20 (2014).
62. Bonomi, L., Huang, Y. & Ohno-Machado, L. Privacy challenges and research opportunities for genomic data sharing. *Nat Genet* **52**, 646–654 (2020).
63. Hekel, R. *et al.* Privacy-preserving storage of sequenced genomic data. *BMC Genomics* **22**, 1–13 (2021).
64. Aggarwal, C. C. On k-anonymity and the curse of dimensionality. in *VLDB* vol. 5 901–909 (2005).
65. Dwork, C. & Roth, A. The algorithmic foundations of differential privacy. *Foundations and Trends® in Theoretical Computer Science* **9**, 211–407 (2014).
66. Rambla, J. *et al.* Beacon v2 and beacon networks: a "lingua franca" for federated data discovery in biomedical genomics, and beyond. *Hum Mutat* **43**, 791–799 (2022).
67. Aziz, M. M. Al, Ghasemi, R., Waliullah, M. & Mohammed, N. Aftermath of bustamante attack on genomic beacon service. *BMC Med Genomics* **10**, 43–54 (2017).
68. Yan, J., Han, Z., Zhou, Y. & Lu, L. A Differential Privacy Approach to Preserve GWAS Data Sharing based on A Game Theoretic Perspective. *KSII Transactions on Internet and Information Systems (TIIS)* **16**, 1028–1046 (2022).
69. Simmons, S., Sahinalp, C. & Berger, B. Enabling privacy-preserving GWASs in heterogeneous human populations. *Cell Syst* **3**, 54–61 (2016).
70. Almadhoun, N., Ayday, E. & Ulusoy, Ö. Differential privacy under dependent tuples—the case of genomic privacy. *Bioinformatics* **36**, 1696–1703 (2020).
71. Makarious, M. B. *et al.* GenoML: automated machine learning for genomics. *arXiv preprint arXiv:2103.03221* (2021).
72. Rieke, N. *et al.* The future of digital health with federated learning. *NPJ Digit Med* **3**, 119 (2020).
73. Boscarino, N., Cartwright, R. A., Fox, K. & Tsosie, K. S. Federated learning and Indigenous genomic data sovereignty. *Nat Mach Intell* 1–3 (2022).
74. Mocanu, I., Smadu, R., Dragoi, M., Mocanu, A. & Cramariuc, O. Testing Federated Learning on Health and Wellbeing Data. in *2021 International Conference on e-Health and Bioengineering (EHB)* 1–4 (IEEE, 2021).
75. Xu, J. *et al.* Federated learning for healthcare informatics. *J Healthc Inform Res* **5**, 1–19 (2021).
76. Kolobkov, D. *et al.* Efficacy of federated learning on genomic data: a study on the UK Biobank and the 1000 Genomes Project. *medRxiv* 2021–2023 (2023).
77. Gosselin, R., Vieu, L., Loukil, F. & Benoit, A. Privacy and Security in Federated Learning: A Survey. *Applied Sciences* **12**, 9901 (2022).
78. Li, N. & Stephens, M. Modeling linkage disequilibrium and identifying recombination hotspots using single-nucleotide polymorphism data. *Genetics* **165**, 2213–2233 (2003).

79. Su, Z., Marchini, J. & Donnelly, P. HAPGEN2: simulation of multiple disease SNPs. *Bioinformatics* **27**, 2304–2305 (2011).
80. Baumdicker, F. *et al.* Efficient ancestry and mutation simulation with msprime 1.0. *Genetics* **220**, iyab229 (2022).
81. Yelmen, B. *et al.* Creating artificial human genomes using generative neural networks. *PLoS Genet* **17**, e1009303 (2021).
82. Atkinson, E. G. *et al.* Cross-ancestry genomic research: time to close the gap. *Neuropsychopharmacology* **47**, 1737–1738 (2022).
83. Oprisanu, B., Ganev, G. & De Cristofaro, E. On utility and privacy in synthetic genomic data. *arXiv preprint arXiv:2102.03314* (2021).
84. Stadler, T., Oprisanu, B. & Troncoso, C. Synthetic data-A privacy mirage. *arXiv preprint arXiv:2011.07018* (2020).
85. Chandra, S., Bhattacharyya, S., Paira, S. & Alam, S. S. A study and analysis on symmetric cryptography. in *2014 International Conference on Science Engineering and Management Research (ICSEMR)* 1–8 (IEEE, 2014).
86. Patel, K. Performance analysis of AES, DES and Blowfish cryptographic algorithms on small and large data files. *International Journal of Information Technology* **11**, 813–819 (2019).
87. Qadir, A. M. & Varol, N. A review paper on cryptography. in *2019 7th international symposium on digital forensics and security (ISDFS)* 1–6 (IEEE, 2019).
88. Jiao, L., Hao, Y. & Feng, D. Stream cipher designs: a review. *Science China Information Sciences* **63**, 1–25 (2020).
89. Serrano, R., Duran, C., Sarmiento, M., Pham, C.-K. & Hoang, T.-T. ChaCha20–Poly1305 Authenticated Encryption with Additional Data for Transport Layer Security 1.3. *Cryptography* **6**, 30 (2022).
90. Senf, A. *et al.* Crypt4GH: a file format standard enabling native access to encrypted data. *Bioinformatics* **37**, 2753–2754 (2021).
91. Hosseini, M., Pratas, D. & Pinho, A. J. Cryfa: a secure encryption tool for genomic data. *Bioinformatics* **35**, 146–148 (2019).
92. Lei, X., Zhu, X., Chi, H. & Jiang, S. Cloud-assisted privacy-preserving genetic paternity test. in *2015 IEEE/CIC International Conference on Communications in China (ICCC)* 1–6 (IEEE, 2015).
93. Kalra, S. & Sood, S. K. Elliptic curve cryptography: survey and its security applications. in *Proceedings of the international conference on advances in computing and artificial intelligence* 102–106 (2011).
94. Jiang, Y., Shang, T. & Liu, J. SM algorithms-based encryption scheme for large genomic data files. *Digital Communications and Networks* **7**, 543–550 (2021).
95. Keller, M. MP-SPDZ: A versatile framework for multi-party computation. in *Proceedings of the 2020 ACM SIGSAC conference on computer and communications security* 1575–1590 (2020).
96. Yao, A. C. Protocols for secure computations. in *23rd annual symposium on foundations of computer science (sfcs 1982)* 160–164 (IEEE, 1982).
97. Bogdanov, D. *et al.* Privacy-preserving statistical data analysis on federated databases. in *Privacy Technologies and Policy: Second Annual Privacy Forum, APF 2014, Athens, Greece, May 20-21, 2014. Proceedings 2* 30–55 (Springer, 2014).
98. Xie, W. *et al.* SecureMA: protecting participant privacy in genetic association meta-analysis. *Bioinformatics* **30**, 3334–3341 (2014).
99. Jagadeesh, K. A., Wu, D. J., Birgmeier, J. A., Boneh, D. & Bejerano, G. Deriving genomic diagnoses without revealing patient genomes. *Science (1979)* **357**, 692–695 (2017).

100. Lauter, K., López-Alt, A. & Naehrig, M. Private computation on encrypted genomic data. in *Progress in Cryptology-LATINCRYPT 2014: Third International Conference on Cryptology and Information Security in Latin America Florianópolis, Brazil, September 17–19, 2014 Revised Selected Papers* 3–27 (Springer, 2015).
101. Kachouh, B., Hariss, K., Sliman, L., Samhat, A. E. & Alsuliman, T. Privacy preservation of genome data analysis using homomorphic encryption. *Service Oriented Computing and Applications* **15**, 273–287 (2021).
102. Gürsoy, G., Chielle, E., Brannon, C. M., Maniatakos, M. & Gerstein, M. Privacy-preserving genotype imputation with fully homomorphic encryption. *Cell Syst* **13**, 173–182 (2022).
103. Blatt, M., Gusev, A., Polyakov, Y. & Goldwasser, S. Secure large-scale genome-wide association studies using homomorphic encryption. *Proceedings of the National Academy of Sciences* **117**, 11608–11613 (2020).
104. Thomson, I. Microsoft researchers smash homomorphic encryption speed barrier. *The Register* (2016).
105. Raisaro, J. L. *et al.* Protecting privacy and security of genomic data in i2b2 with homomorphic encryption and differential privacy. *IEEE/ACM Trans Comput Biol Bioinform* **15**, 1413–1426 (2018).
106. Wu, H. & Wang, F. A survey of noninteractive zero knowledge proof system and its applications. *The Scientific World Journal* **2014**, (2014).
107. Hwang, S., Ozturk, E. & Tsudik, G. Balancing Security and Privacy in Genomic Range Queries. *ACM Transactions on Privacy and Security* **26**, 1–28 (2023).
108. Yang, Y. *et al.* PriGenX: Privacy-preserving Query With Anonymous Access Control for Genomic Data. *IEEE Trans Dependable Secure Comput* (2023).
109. Shor, P. W. Algorithms for quantum computation: discrete logarithms and factoring. in *Proceedings 35th annual symposium on foundations of computer science* 124–134 (Ieee, 1994).
110. O'Neill, P. H. The US is worried that hackers are stealing data today so quantum computers can crack it in a decade. Preprint at (2022).
111. Avanzi, R. *et al.* CRYSTALS-Kyber algorithm specifications and supporting documentation. *NIST PQC Round* **2**, 1–43 (2019).
112. Ducas, L. *et al.* Crystals-dilithium: A lattice-based digital signature scheme. *IACR Transactions on Cryptographic Hardware and Embedded Systems* 238–268 (2018).
113. Soni, D. *et al.* FALCON. *Hardware Architectures for Post-Quantum Digital Signature Schemes* 31–41 (2021).
114. McGuire, A. L. & Beskow, L. M. Informed consent in genomics and genetic research. *Annu Rev Genomics Hum Genet* **11**, 361–381 (2010).
115. Declaration de Helsinki, A. M. M. Ethical Principles for Medical Research Involving Human Subjects. *Recuperado de: http://www. wma. net/es/30publications/10policies/b3* (2013).
116. Ten Have, H. & Jean, M. *The UNESCO universal declaration on bioethics and human rights: Background, principles and application*. (Unesco, 2009).
117. Koplin, J. J., Gyngell, C., Savulescu, J. & Vears, D. F. Moving from 'fully'to 'appropriately'informed consent in genomics: The PROMICE framework. *Bioethics* **36**, 655–665 (2022).
118. Wiertz, S. & Boldt, J. Evaluating models of consent in changing health research environments. *Med Health Care Philos* **25**, 269–280 (2022).
119. Mikkelsen, R. B., Gjerris, M., Waldemar, G. & Sandøe, P. Broad consent for biobanks is best–provided it is also deep. *BMC Med Ethics* **20**, 1–12 (2019).

120. Tindana, P. & de Vries, J. Broad consent for genomic research and biobanking: perspectives from low-and middle-income countries. *Annu Rev Genomics Hum Genet* **17**, 375–393 (2016).
121. Bunnik, E. M., Janssens, A. C. J. W. & Schermer, M. H. N. A tiered-layered-staged model for informed consent in personal genome testing. *European Journal of Human Genetics* **21**, 596–601 (2013).
122. Mascalzoni, D. *et al.* Ten years of dynamic consent in the CHRIS study: informed consent as a dynamic process. *European Journal of Human Genetics* **30**, 1391–1397 (2022).
123. Budin-Ljøsne, I. *et al.* Dynamic consent: a potential solution to some of the challenges of modern biomedical research. *BMC Med Ethics* **18**, 1–10 (2017).
124. Teare, H. J. A., Prictor, M. & Kaye, J. Reflections on dynamic consent in biomedical research: the story so far. *European journal of human genetics* **29**, 649–656 (2021).
125. Teare, H. J. A., Morrison, M., Whitley, E. A. & Kaye, J. Towards 'Engagement 2.0': Insights from a study of dynamic consent with biobank participants. *Digit Health* **1**, 2055207615605644 (2015).
126. Budin-Ljøsne, I., Teare, H., Kaye, J. & Mascalzoni, D. Meta consent: Is it new and is it fit for purpose? *BMJ* **350**, (2016).
127. Chen, C. *et al.* Replacing paper informed consent with electronic informed consent for research in academic medical centers: a scoping review. *AMIA Summits on Translational Science Proceedings* **2020**, 80 (2020).
128. Mishra, R. *et al.* Virtual reality in neurosurgery: beyond neurosurgical planning. *Int J Environ Res Public Health* **19**, 1719 (2022).
129. Xiao, Z., Li, T. W., Karahalios, K. & Sundaram, H. Inform the Uninformed: Improving Online Informed Consent Reading with an AI-Powered Chatbot. in *Proceedings of the 2023 CHI Conference on Human Factors in Computing Systems* 1–17 (2023).
130. Metke-Jimenez, A., Steel, J., Hansen, D. & Lawley, M. Ontoserver: a syndicated terminology server. *J Biomed Semantics* **9**, 1–10 (2018).
131. Haas, M. A. *et al.* 'CTRL': an online, Dynamic Consent and participant engagement platform working towards solving the complexities of consent in genomic research. *European Journal of Human Genetics* **29**, 687–698 (2021).
132. Harvey, L. A. REDCap: web-based software for all types of data storage and collection. *Spinal Cord* **56**, 625 (2018).
133. Prictor, M., Teare, H. J. A. & Kaye, J. Equitable participation in biobanks: the risks and benefits of a "dynamic consent" approach. *Front Public Health* **6**, 253 (2018).
134. Preukschat, A. & Reed, D. *Self-sovereign identity*. (Manning Publications, 2021).
135. Naik, N. & Jenkins, P. Governing principles of self-sovereign identity applied to blockchain enabled privacy preserving identity management systems. in *2020 IEEE International Symposium on Systems Engineering (ISSE)* 1–6 (IEEE, 2020).
136. Román-Martínez, I. *et al.* Blockchain-Based Service-Oriented Architecture for Consent Management, Access Control, and Auditing. *IEEE Access* **11**, 12727–12741 (2023).
137. Thiebes, S., Schlesner, M., Brors, B. & Sunyaev, A. Distributed ledger technology in genomics: a call for Europe. *European Journal of Human Genetics* **28**, 139–140 (2020).
138. Mamo, N., Martin, G. M., Desira, M., Ellul, B. & Ebejer, J.-P. Dwarna: a blockchain solution for dynamic consent in biobanking. *European Journal of Human Genetics* **28**, 609–626 (2020).

139. Albalwy, F., Brass, A. & Davies, A. A blockchain-based dynamic consent architecture to support clinical genomic data sharing (ConsentChain): Proof-of-concept study. *JMIR Med Inform* **9**, e27816 (2021).
140. Androulaki, E. *et al.* Hyperledger fabric: a distributed operating system for permissioned blockchains. in *Proceedings of the thirteenth EuroSys conference* 1–15 (2018).
141. Sunyaev, A. & Sunyaev, A. Distributed ledger technology. *Internet computing: Principles of distributed systems and emerging internet-based technologies* 265–299 (2020).
142. Alghazwi, M., Turkmen, F., Van Der Velde, J. & Karastoyanova, D. Blockchain for genomics: a systematic literature review. *Distributed Ledger Technologies: Research and Practice* **1**, 1–28 (2022).
143. Belchior, R., Vasconcelos, A., Guerreiro, S. & Correia, M. A survey on blockchain interoperability: Past, present, and future trends. *ACM Computing Surveys (CSUR)* **54**, 1–41 (2021).
144. Beyene, M. *et al.* A scoping review of distributed ledger technology in genomics: thematic analysis and directions for future research. *Journal of the American Medical Informatics Association* **29**, 1433–1444 (2022).
145. Alghazwi, M., Turkmen, F., Van Der Velde, J. & Karastoyanova, D. Blockchain for genomics: a systematic literature review. *Distributed Ledger Technologies: Research and Practice* **1**, 1–28 (2022).
146. Metke-Jimenez, A., Steel, J., Hansen, D. & Lawley, M. Ontoserver: a syndicated terminology server. *J Biomed Semantics* **9**, 1–10 (2018).
147. Verbrugge, S. *et al.* Towards a personal data vault society: an interplay between technological and business perspectives. in *2021 60th FITCE Communication Days Congress for ICT Professionals: Industrial Data–Cloud, Low Latency and Privacy (FITCE)* 1–6 (IEEE, 2021).
148. Sambra, A. V. *et al.* Solid: a platform for decentralized social applications based on linked data. *MIT CSAIL & Qatar Computing Research Institute, Tech. Rep.* (2016).
149. Werbrouck, J., Pauwels, P., Beetz, J. & van Berlo, L. Towards a decentralised common data environment using linked building data and the solid ecosystem. in *36th CIB W78 2019 Conference* 113–123 (2019).
150. Aarestrup, F. M. *et al.* Towards a European health research and innovation cloud (HRIC). *Genome Med* **12**, 1–14 (2020).
151. Bíró, K. *et al.* Investigating the knowledge of and public attitudes towards genetic testing within the Visegrad countries: a cross-sectional study. *BMC Public Health* **20**, 1–10 (2020).
152. Zhong, A. *et al.* Ethical, social, and cultural issues related to clinical genetic testing and counseling in low-and middle-income countries: a systematic review. *Genetics in Medicine* **23**, 2270–2280 (2021).
153. Jonassaint, C. R. *et al.* Regional differences in awareness and attitudes regarding genetic testing for disease risk and ancestry. *Hum Genet* **128**, 249–260 (2010).
154. Garrison, N. *et al.* Genomic research through an indigenous lens: understanding the expectations. *Annu Rev Genomics Hum Genet* **20**, 495–517 (2019).
155. Malakar, Y., Lacey, J., Twine, N. A., McCrea, R. & Bauer, D. C. Balancing the safeguarding of privacy and data sharing: perceptions of genomic professionals on patient genomic data ownership in Australia. *European Journal of Human Genetics* 1–7 (2023).
156. Giroux, C. M. & Moreau, K. A. Leveraging social media for medical education: Learning from patients in online spaces. *Med Teach* **42**, 970–972 (2020).

157. Talwar, D., Tseng, T.-S., Foster, M., Xu, L. & Chen, L.-S. Genetics/genomics education for nongenetic health professionals: a systematic literature review. *Genetics in medicine* **19**, 725–732 (2017).
158. Bennett, R. L., Waggoner, D. & Blitzer, M. G. Medical genetics and genomics education: how do we define success? Where do we focus our resources? *Genetics in Medicine* **19**, 751–753 (2017).
159. Stellefson, M., Paige, S. R., Chaney, B. H. & Chaney, J. D. Evolving role of social media in health promotion: updated responsibilities for health education specialists. *Int J Environ Res Public Health* **17**, 1153 (2020).
160. Kawasaki, H., Kawasaki, M., Iki, T. & Matsuyama, R. Genetics education program to help public health nurses improve their knowledge and enhance communities' genetic literacy: A pilot study. *BMC Nurs* **20**, 1–13 (2021).

Cover letter
Click here to access/download;Personal Cover;GigaScience Cover Letter signed.pdf

**CSIRO**
Health and Biosecurity Business Unit
11 Julius Avenue, North Ryde, 2113, Australia

**csiro.au** | ABN 41 687 119 230

14 August 2023

Dear Dr. Goodman and Dr. Edmunds,

We would like to suggest our article titled "Future-proofing genomic data and consent management: a comprehensive review of technology innovations" for publication in GigaScience.

Privacy concerns among patients and stricter data protection laws incentivises research consortia and clinical applications to re-evaluate their approach to genomic data management. For example, a recent Cell publication (Mackey *et al.* 2022), discusses blockchain technologies to empower individuals for indigenous data management, while a range of Oxford Bioinformatics papers (Von Thenen *et al.* 2019; Hosseini *et al.* 2019; Almadhoun *et al*. 2020) emphasise the importance of restricting access for data protection. With data utility being at odds with security, which in turn impedes on the rights of individuals to control their own data, it becomes increasingly more difficult to choose the right approach.

This comprehensive review discusses the benefits and risks of emerging technologies (e.g. decentralised storage, self-sovereign identity, quantum-proof encryption), as well as the overarching themes of good genomic data management (e.g. minimally necessary data sharing, interoperability standards, dynamic consent). It is intended for architecture developers as a curated reference list of available software solutions, as well as for governance and strategy committees to be informed about technology trends, capabilities, and risks. As such, we expect the document to appeal to a wide audience and contribute value to a very topical domain.

We are looking forward to your correspondence.

Sincerely,

Denis Bauer
A/Prof, PhD
Denis.Bauer@CSIRO.au
