# Supplementary material for: Future-proofing genomic data and consent management: a comprehensive review of technology innovations
Source: Gigascience. 2024 Jun 5;13:giae021. doi: 10.1093/gigascience/giae021 (PMC11152178; doi:10.1093/gigascience/giae021)
Supplement: giae021_GIGA_D_23_00243_Revision_2 [file giae021_giga_d_23_00243_revision_2.pdf]

GigaScience

Future-proofing genomic data and consent management: a comprehensive review of technology innovations

--Manuscript Draft--

Manuscript Number:	GIGA-D-23-00243R2
Full Title:	Future-proofing genomic data and consent management: a comprehensive review of technology innovations
Article Type:	Review
Funding Information:	
Abstract:	Genomic information is increasingly used to inform medical treatments and manage future disease risks. However, any personal and societal gains must be carefully balanced against the risk to individuals contributing their genomic data. Expanding our understanding of actionable genomic insights requires researchers to access large global datasets to capture the complexity of genomic contribution to diseases. Similarly, clinicians need efficient access to a patient's genome as well as population-representative historical records for evidence-based decisions. Both researchers and clinicians hence rely on participants to consent to the use of their genomic data, which in turn requires trust in the professional and ethical handling of this information.<br>Here, we review existing and emerging solutions for secure and effective genomic information management, including storage, encryption, consent, and authorisation that are needed to build participant trust. We discuss recent innovations in cloud computing, quantum-computing-proof encryption, and self-sovereign identity. These innovations can augment key developments from within the genomics community, notably GA4GH Passports and the Crypt4GH file container standard. We also explore how decentralised storage as well as digital consenting process can offer culturally acceptable processes to encourage data contributions from ethnic minorities.<br>We conclude that the individual and their right for self-determination needs to be put at the centre of any genomics framework; because only on an individual level can the received benefits be accurately balanced against the risk of exposing private information.
Corresponding Author:	Denis Bauer<br><br>AUSTRALIA
Corresponding Author Secondary Information:	
Corresponding Author's Institution:	
Corresponding Author's Secondary Institution:	
First Author:	Adrien Oliva
First Author Secondary Information:	
Order of Authors:	Adrien Oliva
	Anubhav Kaphle
	Roc Reguant
	Letitia M.F. Sng
	Natalie A Twine
	Yuwan Malakar
	Anuradha Wickramarachchi
	Marcel Keller
	Thilina Ranbaduge

Powered by Editorial Manager® and ProduXion Manager® from Aries Systems Corporation

	Eva K F Chan
	James Breen
	Sam Buckberry
	Boris Guennewig
	Matilda Haas
	Alex Brown
	Mark J Cowley
	Natalie Thorne
	Yatish Jain
	Denis Bauer
**Order of Authors Secondary Information:**	
**Response to Reviewers:**	Dear Dr. Nogoy,<br>We are grateful for the guidance provided during the second round of review of our manuscript. Below, we have responded to the specific comments of reviewer 2.<br>We also used the opportunity to add more information about how GA4GH passports link in with the OAuth 2.0 framework, a central building block of modern web exchanges. The new section reads as follows:<br>To support automation and interoperability across multiple data systems, the GA4GH has developed the GA4GH Passports and Authentication & Authorisation Infrastructure (AAI) specifications73. Passports provide the format for data and resource access permissions, built on top of the openID Connect standards74, which in turn are based on the OAuth 2.0 framework75. They serve as AAI tokens (utilising JSON Web Tokens) to carry a researcher's digital identity and access rights across organisations, tools, environments, encoding each permission/claim as "Visas", allowing access to specific registered-access datasets. Visas are issued if the intended use of the data complies with restrictions set out by the DAC. Uses and restrictions are based on defined terms in the GA4GH Data Use Ontology (DUO) 76, fully automating the whole process, potentially saving months between an access request and approval.<br><br>Reviewer #2:<br>1)My assessment is that this paper still has issues with its framing and structure. The work lacks clarity in its scope - while you have addressed some of the writing prompts in the reviewer comments, you have not engaged with any substantive suggestions.<br>RESPONSE: While we have not converted the document into a systematic review as suggested by the reviewer, we outlined our reasons for why we feel a literature review is the more suitable format.<br><br>2)The reference to Genomical without any discussion of the market it sits in or competitors in this space is problematic. There is no evidence provided to support the claims made about this company, e.g., a relevant white paper or research paper with statistics, or linking the organization's approach to scientific development or theory.<br>RESPONSE: We have removed references to the company, and instead referred to their peer-reviewed technology as developed under the public funding of the Australian and Melbourne Genomics Health Alliance, supported by the Victorian Government. The new paragraph reads as following:<br><br>For example, Melbourne Genomics Health Alliance, a collaboration of leading Victorian hospitals, research, and academic institutions working with the Victorian Government to embed genomics into the health system, developed a cloud solution that benefits from international developments in security, scalability, and health system interoperability, yet seeks to minimise third-party dependence by implementing federated data governance controls. Specifically, the genomic information management system, Genomical (previously known as GenoVic53) offers a robust clinical data governance framework and implemented capability for controlled access and reuse of data between authorised entities for the purpose of clinical care, therefore, providing foundations for data reuse within the healthcare system where it is

	adopted.<br><br>53. Thorne, N. et al. Genovic: a secure, scalable and modular system for genomic testing. Pathology 51, S120–S121 (2019).<br><br><br>3)The discussion of dynamic consent is based on theory papers that themselves suppose advantages of the model, rather than make claims based in empirical data. Claims are made here without either a) a critical analysis of the literature, or b) evidence to support or refute the claim made.<br>RESPONSE: We have expanded the theoretical papers on dynamic consent with papers outlining the evidence for dynamic consent. The new section reads as follows and refers to these citations:<br>The dynamic consent model focuses on enhancing continuous engagement of participants through personalised online consent processes (e.g. Dynamic specific consent 142) and digital communication platforms148–151. It is believed that dynamic consent positively influences both the recruitment and retention of participants as well as their trust towards research148,150,152,153, while also contributing to the proficient management of the informed consent procedure148.<br><br>148.Mascalzoni, D. et al. Ten years of dynamic consent in the CHRIS study: informed consent as a dynamic process. European Journal of Human Genetics 30, 1391–1397 (2022).<br>151.Teare, H. J. A., Prictor, M. & Kaye, J. Reflections on dynamic consent in biomedical research: the story so far. European journal of human genetics 29, 649–656 (2021).<br>152.Haas, M. A., Madelli, E. O., Brown, R., Prictor, M. & Boughtwood, T. Evaluation of CTRL: a web application for dynamic consent and engagement with individuals involved in a cardiovascular genetic disorders cohort. European Journal of Human Genetics 1–8 (2023).<br>153.Spencer, K. et al. Patient perspectives on sharing anonymized personal health data using a digital system for dynamic consent and research feedback: a qualitative study. J Med Internet Res 18, e5011 (2016).<br><br><br>4)I congratulate you on the framework you have produced, but it is unclear as to why you are centering the individual when the framework centers on 'Governance and Legislation, with various stakeholder groups around it.<br><br>RESPONSE: Our diagram (Figure 1) visualizes “Governance and Legislation” in the middle and has the individual at the top, in line with the pyramided of trust in SSI relationships. We agree that the figure of speech “putting the individual at the centre” might be confusing in this context. We hence changed the description for the diagram to now read:<br>We envision a system that, while subject to governance and law, puts the individual at the top of the decision-making process (Figure 1).
**Additional Information:**	
**Question**	**Response**
Are you submitting this manuscript to a special series or article collection?	No
**Experimental design and statistics**<br><br><br>Full details of the experimental design and statistical methods used should be given in the Methods section, as detailed in our Minimum Standards Reporting Checklist. Information essential to interpreting the	Yes

data presented should be made available in the figure legends.<br><br>Have you included all the information requested in your manuscript?	
**Resources**<br><br>A description of all resources used, including antibodies, cell lines, animals and software tools, with enough information to allow them to be uniquely identified, should be included in the Methods section. Authors are strongly encouraged to cite Research Resource Identifiers (RRIDs) for antibodies, model organisms and tools, where possible.<br><br>Have you included the information requested as detailed in our Minimum Standards Reporting Checklist?	Yes
**Availability of data and materials**<br><br>All datasets and code on which the conclusions of the paper rely must be either included in your submission or deposited in publicly available repositories (where available and ethically appropriate), referencing such data using a unique identifier in the references and in the “Availability of Data and Materials” section of your manuscript.<br><br>Have you have met the above requirement as detailed in our Minimum Standards Reporting Checklist?	Yes

Manuscript

Click here to access/download;Manuscript;Dynamic_consent_review2_clean

Future-proofing genomic data and consent management: a comprehensive review of technology innovations

Adrien Oliva[1*], Anubhav Kaphle[1*], Roc Reguant[1], Letitia M.F. Sng[1], Natalie A. Twine [1], Yuwan Malakar[2], Anuradha Wickramarachchi[1], Marcel Keller[3], Thilina Ranbaduge[4], Eva K F Chan[5], James Breen[6,7], Sam Buckberry[6,7], Boris Guennewig[8], Matilda Haas[10,11], Alex Brown[6,7], Mark J Cowley[10,11], Natalie Thorne[12,13,14,15], Yatish Jain[1,116], Denis C. Bauer[1,16,17]

[1] Australian e-Health Research Centre, Commonwealth Scientific and Industrial Research Organisation, Westmead, Australia

[2] Responsible Innovation Future Science Platform, Commonwealth Scientific and Industrial Research Organisation, Brisbane, Australia

[3] Data61, Commonwealth Scientific and Industrial Research Organisation, Black Mountain, Canberra, Australia

[4] Data61, Commonwealth Scientific and Industrial Research Organisation, Eveleigh, Australia

[5] NSW Health Pathology, Sydney, New South Wales, Australia.

[6] Telethon Kids Institute, Perth, WA 6009, Australia.

[7] National Centre for Indigenous Genomics, The John Curtin School of Medical Research, Australian National University, Canberra, ACT 2601, Australia.

[8] Sydney Medical School, Brain and Mind Centre, The University of Sydney, Sydney, NSW, Australia.

[10] Children's Cancer Institute, Lowy Cancer Research Centre, UNSW Sydney, Sydney, NSW, Australia.

[11] School of Clinical Medicine, UNSW Medicine & Health, UNSW Sydney, Sydney, NSW, Australia

[12] Murdoch Children's Research Institute, Parkville, Australia 3052

[13] University of Melbourne, Melbourne, VIC, Australia

[14] Melbourne Genomics Health Alliance, Melbourne, VIC, Australia

[15] Walter and Eliza Hall Institute, Melbourne, VIC, Australia

[16] Macquarie University, Applied BioSciences, Faculty of Science and Engineering, Macquarie Park, Australia

[17] Macquarie University, Department of Biomedical Sciences, Macquarie Park, Australia

*Co-first authors

Genomic information is increasingly used to inform medical treatments and manage future disease risks. However, any personal and societal gains must be carefully balanced against the risk to individuals contributing their genomic data. Expanding our understanding of actionable genomic insights requires researchers to access large global datasets to capture the complexity of genomic contribution to diseases. Similarly, clinicians need efficient access to a patient's genome as well as population-representative historical records for evidence-based decisions. Both researchers and clinicians hence rely on participants to consent to the use of their genomic data, which in turn requires trust in the professional and ethical handling of this information.

Here, we review existing and emerging solutions for secure and effective genomic information management, including storage, encryption, consent, and authorisation that are needed to build participant trust. We discuss recent innovations in cloud computing, quantum-computing-proof encryption, and self-sovereign identity. These innovations can augment key developments from within the genomics community, notably GA4GH Passports and the Crypt4GH file container standard. We also explore how decentralised storage as well as digital consenting process can offer culturally acceptable processes to encourage data contributions from ethnic minorities.

We conclude that the individual and their right for self-determination needs to be put at the centre of any genomics framework; because only on an individual level can the received benefits be accurately balanced against the risk of exposing private information.

Keywords Genome data privacy, Trust model, Decentralised systems, Self-sovereign identity, Dynamic Consent

Table of Contents

Future-proofing genomic data and consent management: a comprehensive review of technology innovations **1**

Introduction **3**

Genomic Data Storage Solutions **5**

On-Premises Storage *6*

Cloud Storage *7*

Hybrid Storage *8*

Decentralised Storage *8*

Genomic Data Privacy and Security **9**

Availability *9*

Integrity and Privacy-preserving Techniques *10*
Federated learning (FL) 12
Privacy-preserving synthetic genomic data 13
Confidentiality and cryptography methods *13*
Encryption 14
Multi-party computation (MPC) 15
Post-quantum cryptography (PQC) 16
Informed Consent Management **17**
Consent Models *17*
Broad Consent 17
Tiered Consent 17
Dynamic Consent 18
Digital Systems for Consent Management *18*
Decentralised approaches *19*
Decentralised identity 20
Immutable ledger technology 20
Personal data server 21
Conclusion **24**
Acknowledgement *25*
Conflict of Interest *25*
References *26*

Introduction

Over 60 million individuals are estimated to have their genomes sequenced in a healthcare context by 2025 [1]. This increase can be attributed to the decreasing cost of genome sequencing [2–5], the rise of direct-to-consumer (DTC) genetic testing companies [6], the integration of genome testing into public healthcare systems, and the launch of large-scale population genomics initiatives in numerous countries [7–11]. However, digital infrastructure, software solutions, data security measures, and legal frameworks for managing big genomic data have not kept pace with these rapid advancements. Notably lacking are advancements in ethical data management, efficient data sharing, and data sovereignty [12]. Addressing these aspects is essential to ensure the continued participation of a privacy-aware public, especially from marginalised communities, in contributing their private information to research [13,14].

The challenges become even more pronounced when applying genomics in a clinical context. Here, the generated data directly impacts patient care while also having value in broadening scientific knowledge. Clinicians, typically rely on pathology providers to generate reports summarising the genomic information to inform patient care. These

reports are generated by multidisciplinary teams [15] but in the remaining document we will refer to them as "pathology providers" for simplicity. While established clinical governance and security standards provide guidance for the management, storage, and analysis of genomic data for clinical care, it often conflicts with the need for broader access and sharing of this data for research[15] . Platforms able to serve both clinical and research applications need to resolve the tension between the protective regulations in clinical settings and the exploratory objectives of research, as well as enable interoperability across the two domains.

Key concerns in genomic data management are privacy attacks that exploit an individual's sensitive health and ancestry information, particularly when clinically generated genomic data is reused for research purposes. For instance, *identification attacks,* can link an individual's genomic data with publicly available information, such as demographic data or family history to triangulate on target individuals[16,17] . Current research practices of masking personally identifying variants, such as rare single nucleotide polymorphism (SNPs) or germline variants, are not sufficiently protective[18]. Even if the genomic sequence is not ascertained directly, in a *membership inference attack* the adversary can infer the membership status of individuals in genomic research studies, such as rare disease genome-wide association studies (GWAS), by leveraging allele frequencies from public databases [19,20]. This risk is exacerbated with the increasing number of large-scale national or regional studies that recruit all participants that meet broad eligibility criteria[21]. This also extends to an individual's physical traits, demographic information, and disease susceptibility, which can be obtained through *phenotype inference attacks* using genomic data[22,23]. In these studies, whole genomic sequencing and detailed phenotyping were used to predict biometric traits including voice, biological age, and 3D facial structure [24].

To generate scientific outcomes that are robust, clinically meaningful, ethical, and equitable, genomic data needs to have ethnically diverse representation [25]. However, to achieve this diversity, it is essential to acknowledge that the socio-cultural context of genomic data management extends beyond individual perspectives and involves collective experiences and histories that can shape attitudes towards genomic data sharing. This is especially pertinent for historical instances of discrimination, trauma, racism, stigma, and marginalisation [26]. These collective experiences and cultural connotations significantly influence an individual's or population's perception of the risks associated with genomic data management, creating an intricate landscape that navigates the potential misuse of this data against the interests of certain groups or populations.

Enabling a safe way forward, legislation will have to provide active governance and enforce ethical genomic data usage as started by HIPAA [27] and others [28,29]. This is especially difficult around participants' right-to-be-forgotten, so far only required by GDPR [30], [27] which can be at odds with commercial incentives. For example, while the Australian Financial Services Council specified in their Life Code in July 2023 that

genetic results cannot be used in underwriting certain life insurance policies [31], this is not the case in other countries or sectors, leaving individuals vulnerable to disadvantages from intended or incidental findings of genetic testing.

Given these complexities, a strong and trusted technological foundation for genomic data governance and management is crucial. We explore both proven and emerging solutions and concepts in this review and suggest a framework based on decentralised identity concepts for genomic data and consent management.

Genomic Data Storage Solutions

Secure data storage is essential, for genomic and healthcare data [32–34], specifically on cybersecurity issues such as data breaches, unauthorised access, or malicious attacks[35]. The key advantages and disadvantages of the four approaches reviewed are outlined in Table 1.

Table 1. Different storage solutions' key advantages and disadvantages for effective genomic data management.

Criteria	On-Premises	Cloud	Hybrid	Decentralised
Security	Low risk of attacks when operating offline.	Physical control over servers through contractual agreements, but state-of-the-art audited cybersecurity.	Flexibility to adapt to sensitivity levels.	Technology theoretically renders attacks on stored data impossible.
Scalability	Slow adaption to changes and sudden spikes in workload.	Excels in adapting to dynamic changes and handling sudden increases in workload efficiently.	Balanced solution to manage varying workload.	Scalability is native to the solution.
Data backups	Need to be implemented by the organisation, requiring skills, and scaling up the infrastructural capabilities.	Automated data backups immediately upon the upload of data.	Depends on the architecture deployed.	Built-in data back-up feature.

Skills required	A singular set of specific skills is requisite.	A singular set of specific skills is requisite.	Deploying an optimal system necessitates twice the skillset.	Still experimental requires non-standard skill set
Data control	Complete control and access to servers, data, and associated rights by the data custodian.	Comprehensive control and access to data, servers, and related privileges within a virtualized environment.	Depends on the architecture deployed.	No control over where data is stored but full control of access by participants.
Price	Involves an initial and ongoing capital investment, maintenance, and considerations for expenses related to operational costs, potential equipment failures, and associated bills.	With various storage options (cold / hot), the cost is economical and free for idle time.	Depends on the architecture deployed.	Can be the most economical choice, but for certain options, both pricing and reliability are contingent on the value of the associated token.

On-Premises Storage

On-premises data management refers to storing and managing data within the physical premises of an organisation, providing complete control over data infrastructure, customising storage environment and meeting its unique needs. Storing on-premises can be highly secure when there is no external network as data access can be physically limited to only authorised personnel. This hence represents an easy option for organisations to comply with data privacy and security regulations [27,28,36]. However, it also creates undesirable information silos, especially for the health care setting. Data integration and global research collaborations need carefully managed exposure to the Internet [37], which requires significant expertise, constant monitoring, and substantial time and resource investment to establish and oversee security protocols. In addition, on-premises data management requires an upfront investment, and replacement at relatively frequent 3–5-year intervals for the necessary infrastructure, disadvantaging smaller organisations. Ongoing costs such as energy expenses, broadband access, software licenses, certifications, IT services, and physical space to accommodate the hardware must also be considered. Furthermore, on-premises storage is not suited for short-term spikes in workload[38] as the infrastructure is static and expansion or update is expensive and time-consuming.

Cloud Storage

Data owners can store and manage their data with a public cloud provider [39]. As organisations do not build and manage their infrastructure, capital investments are shifted towards resource consumption and recorded as operational expenses. This allows organisations to pay and scale infrastructure to their changing needs, however, can clash with CapEx-based funding cycles. While legislation to keep medical data inside the countries' jurisdiction has limited cloud usage in the past, cloud providers have responded by opening more in-country data centres and enabling policy configurations that ensure data and back-up remain compliant [40,41]. For example, governments use such policy-optimised clouds for their operations (e.g. AWS GovCloud [42] and AZURE Government [43]). Furthermore, managing data and analysis in the cloud enables seamless global collaborations and ensures reproducible results. However, this scalability and convenience, comes with a higher security risk for the data as the uniformity of cloud account structures makes them attractive targets for hackers [44–47]. To mitigate security risks, cloud providers implement automatic counter measures and equip users with world-class security measures, including access controls through IP address restriction, continuous threat monitoring, encryption for data in transit and at rest, network and application security, data redundancy, and multi-factor authentication [48–51]. While the economy of scale stems most of the costs for security, scalability and global connectedness, cloud usage can become expensive especially for egress-heavy applications, and for users that do not implement auto-archiving retention policies to take advantage of low-cost cloud storage options (like AWS Glacier or Azure Archive). Alternatively, egress cost can be avoided altogether by using federated systems, where compute is brought to the data, for example recently employed by UK Biobank Research Access Platform[52].

Finally, building health care critical infrastructure on – for many users – foreign national cloud providers raise concerns around sovereignty and the limits to oversight, which is exacerbated when data is collected in the clinical setting and reused in research. Public cloud uses hence needs to be carefully balanced against the benefits a globally connected economic health system can provide.

For example, Melbourne Genomics Health Alliance, a collaboration of leading Victorian hospitals, research, and academic institutions working with the Victorian Government to embed genomics into the health system, developed a cloud solution that benefits from international developments in security, scalability, and health system interoperability, yet seeks to minimise third-party dependence by implementing federated data governance controls. Specifically, the genomic information management system, Genomical (previously known as GenoVic [53]) offers a robust clinical data governance framework and implemented capability for controlled access and reuse of data between authorised entities for the purpose of clinical care, therefore, providing foundations for data reuse within the healthcare system where it is adopted.

Hybrid Storage

Hybrid storage solutions can provide the best of both worlds – combining the benefits of on-premises and cloud data management solutions. To adopt such an approach, organisations require a workforce skilled in both domains. By leveraging hybrid solutions, organisations can maintain a local infrastructure for sensitive data and analytics, while easily connecting storage to additional resources in the public cloud when needed, such as processing spiky analysis workloads, or genomic data processing pipelines that require different computing types. However, it comes at the expense of egress costs, potential duplication of effort for system maintenance, and limited access to clinically generated data for healthcare or research purposes.

It allows a staged transition to the cloud where scalability and global connectivity can be realised, without the need to move all data to the cloud at once. This also ensures sovereignty by maintaining some compute and data storage capabilities.

On-premises and cloud computing infrastructure are hence complementary pieces of the puzzle that can help research organisations achieve their goals.

For instance, the Australian *Zero Childhood Cancer Program* [54] houses genomic data on a cloud-connected NetApp StorageGRID within a dedicated partition in an Equinix data centre (see www.equinix.com). This configuration enables standardised data sharing via object store protocols, allowing integration with cloud providers, genomic analysis platforms like CAVATICA (see www.cavatica.org) and national high performance computing resources. Through automated archiving, processed data is retained on-site, reducing long-term cloud storage costs, and data is shared with researchers through unique and secure s3 links. The program mitigates system's egress costs by routing network traffic through academic networks like AARNet [55] where possible and leveraging Equinix Fabric for global collaborations.

Decentralised Storage

Decentralised storage is a Web3 concept [56] where files are fragmented, encrypted, and stored over separate nodes in a decentralised or peer-to-peer (P2P) network [57]. By distributing data across different nodes, it is more secure than being stored in a single "honey pot". It also improves scalability and availability over on-premises and cloud solutions, as the P2P network can be continuously expanded with commodity hardware that is easy to onboard.

The InterPlanetary File System (IPFS) [58] was one of the first decentralised file storage systems developed and originally used as the storage layer for blockchains. IPFS uses cryptographic hashes that are based on the content of the file, thereby eliminating duplicates, and ensuring data integrity. IPFS can store and share massive amounts of data in a decentralised and economical manner which is crucial for genomic projects [59–61]. However, unlike traditional data centres, decentralised file systems are not funded through a single entity. While more traditional blockchain approaches have built-in incentive structure [62] , IPFS relies on good-will from the P2P Network notes and is, therefore, not suitable for operating critical infrastructure, such as health care.

FileCoin is a separate and independent decentralised protocol built on IPFS that is incentivised to offer their storage space by receiving digital currencies as a reward. Various protocols ensure the integrity, security, availability, and accessibility of the data stored on the network [63].

While these incentives are aimed to ensure quality and make storage sustainable, it is crucial to acknowledge the potential risks associated with this approach. Fraudulent projects and initial coin offerings (ICOs) have exploited users in the past by marketing themselves as investment options [64,65]. The risk of being used as a speculative commodity poses a significant risk to the stability of decentralised services, as the digital currencies market value can undermine the platform's incentive structure and functioning. Specifically, if the value of a coin declines, the motivation for nodes to continue storing data ceases, resulting in the loss of irreplaceable medical information. Given these observations, it is crucial to have a careful technical, economic, and ethical evaluation of those systems, especially in the health space.

Genomic Data Privacy and Security

Irrespective of where genomic data is stored, the individual needs to be protected from unauthorised access to their data (privacy) and the data needs to be kept safe from threats, breaches, unauthorised tampering (security). We explore this topic under the criteria of data availability, integrity, and confidentiality (AIC)[66], sometimes also referred to as the "CIA triad".

Availability

Availability ensures timely and uninterrupted access to the genomic data system by authorised users only. Human genomic data is generally protected, and access is only approved if relevant requirements are met. While clinical data can be managed by clinical governance principles, the viability of data usage in research requires Data Access Committees (DACs) to review access requests and ensure that the intended use of the data is permitted by the provided consent [67]. All public genomic data repositories work on this premise, including the database of genotypes and phenotypes (dbGaP), the European Genome-Phenome Archive (EGA), and the UK Biobank [68,69] .

While this protects data and participants, it is a manual process that is not easily scalable, making datasets hard to discover and limiting their use for clinical or research benefits. The Global Alliance for Genomics and Health (GA4GH [70]) has introduced the third access tier, apart from existing open access and controlled access called "registered access" to automate consent mechanisms and address some of the problems. The registered access [71] tier, is intended to allow access to low-risk data for research use, and it requires the user to be a 'bona-fide researcher', in addition to agreeing to the terms of use for the data.

To support automation and interoperability across multiple data systems, the GA4GH has developed the GA4GH Passports and Authentication & Authorisation

Infrastructure (AAI) specifications [72]. Passports provide the format for data and resource access permissions, built on top of the openID Connect standards [73], which in turn are based on the OAuth 2.0 framework [74]. They serve as AAI tokens (utilising JSON Web Tokens) to carry a researcher's digital identity and access rights across organisations, tools, environments, encoding each permission/claim as "Visas", allowing access to specific registered-access datasets. Visas are issued if the intended use of the data complies with restrictions set out by the DAC. Uses and restrictions are based on defined terms in the GA4GH Data Use Ontology (DUO) [75], fully automating the whole process, potentially saving months between an access request and approval.

Availability can be compromised by both non-malicious factors (such as hardware failures, software downtime, resource limits, unmanaged concurrency, and network congestion, natural disaster) and malicious attacks (such as denial of service, also known as DoS attacks) that aim to disrupt the system's functionality. Therefore, technical, and operational security measures such as redundancy, backup, load balancing, and encryption are also essential to protect the system from threats to availability.

Integrity and Privacy-preserving Techniques

The second pillar of AIC [66], data integrity practices, aims to store, and handle the data to prevent accidental or unauthorised modification throughout its entire lifecycle. Genomic data can be efficiently verified and compared by matching hash values using deterministic, collision-resistant, and non-invertible [76] cryptographic hash [77] functions such as Message Digest (MD) and the Secure Hash Algorithm (SHA) [78]. This allows for the identification of any unauthorised changes or alterations to genetic sequences without the need to compare the entire sequence. Furthermore, data hash values can be cryptographically signed [79] using a private key. This digital signature can be verified using the corresponding public key, ensuring the data's authenticity, integrity, and trustworthiness.

The privacy of the genomic data can be protected through four approaches summarised in Table 2. It should be noted that this section focuses on the research setting as in healthcare patient information cannot be obfuscated.

Table 2: Advantages and disadvantages of different privacy preservation approaches

**Criteria**	***k*-anonymity**	**Differential privacy**	**Federated learning**	**Synthetic data methods**
Implementation complexity	Simple and intuitive. Data is grouped in sets to obfuscate	Challenging implementation due to the intricate	Challenges in coordinating local model and with	Challenging to conservatively maintain the statistical properties of real genomic data

	individual entries.	calibration of noise.	network latency, trust, and incentive among peers.	but works as real data statistically.
Privacy Assurance	Cannot fully prevent attribute disclosure or homogeneity attack.	Robust privacy is achieved through the addition of statistical noise to the data, necessitating the quantification of risks through careful control of the trade-off between utility and privacy.	The potential for data leakage risk arises from intermediate model updates, which may expose sensitive information. Additionally, there remains a possibility of backdoor attacks.	May not guarantee perfect privacy and vulnerability to membership inference even when generating distributions close to real data.
Accuracy / Information loss	Homogeneity attacks pose a risk when individuals within a group may disclose sensitive information due to indistinguishability based on certain attributes.	Deliberate introduction of noise can reduce the quality or utility of the resultant data.	The original data remains unaltered and can only be removed by the owners or those with granted permissions. Can be subject to data poisoning attack due to involvement of dishonest peers.	Despite being synthetic and closely resembling real data, it can introduce bias and errors.

k-anonymity

The *k*-anonymity approach works by ensuring that the quasi-identifier for each person, such as their gender, birth date, postal code, race, ethnicity, or occupation, is indistinguishable from at least k-1 individuals in the same dataset [80,81]. This is done by using two approaches: (1) generalisation, which groups individuals together with

similar attributes [82] and (2) suppression**,** which removes certain information to prevent re-identification. For example, one way to suppress genomic data is to remove germline variants, which are inherited from parents and can be used to link individuals across databases. However, this may not completely eliminate the risk of re-identification, as other types of variants or genomic features may still be informative[83,84]. Despite their widespread use, these *k*-anonymity approaches are vulnerable to attackers who have background information on the dataset [85] and are limited for high dimensional genomic data [86].

Differential Privacy (DP)

Differential privacy is a mathematical framework that provides formal and provable privacy protection by introducing calibrated noise to raw data or intermediate results, making it difficult for attackers to trace data records to specific individuals [87]. The amount of noise added depends on various factors including the query type, privacy budget determining the level of privacy required, and the sensitivity of the mathematical function being computed or the query output.
In genomics, DP techniques have been proposed to counteract membership inference attacks, for example by adding noise to a genomic Beacon query response, the genome data discovery tool by GA4GH [88]. The amount of noise is carefully calibrated to balance two goals: to preserve the accuracy and hence the utility of the application and to make it harder for attackers to extract the original genomic data from the query response[89]. Additionally, DP methods for GWAS have provided maximum privacy to participants while still finding meaningful disease associations [90–92]. Although promising, the added noise in DP schemes limits its application to datasets with strong, well understood signals, e.g., disease loci with strong effect size.

Federated learning (FL)

Federated learning (FL) is a machine learning technique that enables multiple parties to jointly train an algorithm without sharing their data thereby avoiding risks to data integrity or having to negotiate data access [93] . In this approach, computations are performed locally on the data that remains within the owner's ecosystem (e.g., server nodes, jurisdictions). These locally trained parameters are then sent to a central server that aggregates the local models from all participating peers to generate a global model shared by all [94,95]. Federated learning has been used on health data [96,97] and was shown on genomics data to achieve comparable performance compared to a centralised approach for phenotype prediction on genomic data using the UK Biobank [98].
While promising, coordinating local model aggregates can be challenging, especially when training complex FL models. Issues such as, network latency, maintaining trust and incentive among participating peers, and ensuring data quality and diversity, remains unresolved. Further, FL is specifically vulnerable to data poisoning attacks,

where attackers deliberately manipulate or corrupts the data, and backdoor attacks by poisoning models to output biased results [99].

Privacy-preserving synthetic genomic data

Creating synthetic genomic data can sidestep many data privacy issues. This data has the same statistical properties as the original dataset, but without passing on the real genomes. Several methods have been developed to generate synthetic genomes leveraging various sources of knowledge including haplotype information [100,101], demographic information and recombination inferences [102]. More advanced methods like deep neural network-based methods, such as generative adversarial networks (GANs) and restricted Boltzmann machines (RBMs) have also successfully generated synthetic genomic data where population structure and variant frequency-based features were preserved [103]. Generative methods can be utilised to create datasets that act as proxies for under-represented populations, going some way to address the known Eurocentric bias in genomic studies [104]. However, the utility of synthetic genomes is limited to evaluating algorithms, rather than for discovery projects, because they do not have more information than the original data, which is further limited by the fidelity of the generative model used.

A recent study by Oprisanu *et al.* [105] compared the above synthetic data methods for utility and privacy. They showed that recombination-based methods have high utility but low privacy, while RBMs offer a trade-off. It is worth noting that generating distributions close to the real data often generates target data points that are vulnerable to membership inference[106]. Therefore, some data integrity and security practices must be enforced even for synthetic data generated by current approaches.

Confidentiality and cryptography methods

Confidentiality represents the third pillar of AIC, which prevents unauthorised access or disclosure of data. We review the five most relevant and noteworthy techniques as summarised in Table 3.

Table 3 Advantages and disadvantages of different data security approaches

**Criteria**	**Symmetric Encryption**	**Asymmetric Encryption**	**Multi Party Computation**	**Homomorphic Encryption**	**Post Quantum**
Scalable performance	Fast and efficient for large genomic datasets.	Slower for encrypting large-scale genomic data,	Computationally expensive, making it less practical for real-time	Well-suited for cloud-based genomic data processing, but computationally intensive	Difficult to scale currently for large-scale datasets.

		potentially delaying data transfer.	analysis of genomic data.	with prolonged processing times.	
Collaboration Suitability	Less suitable if multiple parties are involved due to single key reliance.	The public key can be shared with collaborators, ensuring secure data transfers between institutions without compromising integrity.	Enables collaborative work among institutions without disclosing each other's data.	Facilitates collaboration easily through cloud capabilities yet challenging to set up for all collaborations.	Potentially suitable but depends on the development of practical and efficient protocols that are easily adoptable by multiple parties.
Complexity	Straightforward cryptographic key management due to only one key involved.	Key management can be complicated for multiple parties.	Challenging setup complexity and dependence on collaborating parties.	Initial setup and implementation can be challenging.	Complex in terms of development and implementation; often requires significant expertise and resources to correctly deploy.
Robustness	Risky if key compromised in collaborative settings; secure with robust key management.	Risky if key compromised in collaborative settings; secure with robust key management.	Maximal collaboration security: genomic data consistently encrypted, demands participant trust to prevent data poisoning.	Great as it allows computations on encrypted data, without decrypting it first.	Robust defence against quantum attacks for long-term genomic data security, currently in developmental stages with limited adoption.

Encryption

Encryption is a cryptographic method that aims to secure genomic files by converting *plain text* to *cipher text* using different algebraic operations.

Symmetric encryption methods encrypt data using either stream ciphers such as Salsa20, CHACHA20, and AES-CTR [107] or block ciphers such as the Advanced

Encryption Standard (AES) [108] and are a popular method for securing genomic data as they are fast and efficient [109]. For large genomic data, CHACHA20 is the fastest and most efficient algorithm [110], and is often used in combination with POLY1305, a message authentication code, to ensure message integrity and authentication [111]. For example, CHACHA20-POLY1305 is used in Crypt4GH [112] , a file container standard proposed by GA4GH. Its user-specific envelop encryption scheme enables random byte-level access to encrypted file content without decrypting the whole file. Block ciphers have also been used in genomic data encryption [113] .

Asymmetric or public cryptography schemes, such as Rivest-Shamir-Adleman (RSA) and elliptic curve cryptography (ECC), use two keys for encryption: a public key for encryption and a private key for decryption. The RSA algorithm has been used for genomic data to mask individual's alleles and secure cloud-based genetic paternity test results [85,114], however, it can be slow and impractical for large WGS files [114] and is therefore limited to smaller genomic files and sensitive metadata. ECC is often preferred over RSA for smaller genomic files due to its smaller key sizes and lower computational cost [115,116].

Multi-party computation (MPC)

Multiparty computation improves upon the traditional route of encrypting data solely for storage and transport purposes, which subsequently requires decryption and handling in an unencrypted manner during analysis. MPC frameworks allow researchers to collaborate on data analysis while maintaining privacy using cryptographic methods. While still an emerging technology, it has made rapid progress over the last few years with open-source frameworks such as MP-SPDZ [117] , which combines several MPC variants with an accessible Python user geared toward analytics. Here, we review (1) secret sharing, (2) garbled circuit method, (3) homomorphic encryption and (4) zero-knowledge proof.

Secret sharing

A core technology to most MPC protocols is secret sharing, which denotes the distributed storage of information such that the parties together can reconstruct the information, but an individual party (or a small set) cannot. In some settings, secret-sharing techniques alone can be used for privacy-preserving analytics without the more expensive techniques below. These protocols have been found relatively efficient, enabling even the training of smaller deep learning models.

Garbled circuit method

A popular method of developing MPC is the garbled circuit method where the function is transformed into a Boolean circuit of logic gates and encrypted to produce garbled output values [118]. Collaborators can then use garbled values and their input to

generate an output, ensuring privacy and correctness. In the genomic space, the garbled circuit solution has been used for secure genomic data analysis [119,120] and diagnosis [121], but this approach can be expensive for large genomic data.

Homomorphic encryption

Homomorphic encryption (HE) is a cryptographic technique that allows computations to be performed on encrypted data without decrypting them first. This means that the data can be processed securely without revealing any sensitive information to the parties involved. This can be useful for genome queries and statistical analyses such as GWAS, which aim to find genetic variants associated with certain traits or diseases. Several methods have been developed for HE-based genome queries and statistical analyses [122–126]. However, HE requires more computational resources and time than regular encryption methods, however, new algorithms and techniques have been proposed to improve performance [127]. Combining HE with MPC is argued to further reduce overhead compared to traditional encryption methods. It has shown potential for encrypted control over genomic data to enhance trust within genomics research programs. Additionally, techniques that combine HE with differential privacy can be employed to ensure the security of genomic data and enable privacy while sharing summary results[128] .

Zero-knowledge proof

Zero-knowledge proof (ZKP) is a cryptographic method allowing one party to prove a claim's correctness to another party without revealing additional information [129]. In genomics, ZKP has been used to enable secure genomic query [130], and sequence similarity search [131]. ZKP methods can be hard to set-up, requiring subject matter expertise and they are not scalable due to high compute power on large dataset.

Post-quantum cryptography (PQC)

There is growing concern among information security experts that most of the currently available cryptographic methods, such as RSA and ECC, are vulnerable to attacks by quantum computers (e.g., Shor's algorithm[132]). Although quantum computers are expensive and still developing, it is feared that attackers are already downloading sensitive data to decrypt in the future when quantum computers become more readily available[133]. This has significant implications for genomic data encrypted by today's cryptographic methods as genomic data retains its relevancy over an individual's lifespan and even that of their direct descendants.

In response, the National Institute of Standards and Technology (NIST) in the United States have announced encryption algorithms that were designed to resist attacks from classical and quantum computers thereby enabling post-quantum migration of cryptosystems. These include lattice-based algorithms CRYSTALS-Kyber[134] for general purpose encryption, CRYSTALS-Dilithium[135], and FALCON[136], both for digital

signatures. Using such PQC methods for genomic data now is a proactive step to ensuring that genomic data is protected against possible future attacks while saving time and money rather than re-encrypting when quantum computers become more accessible.

Informed Consent Management

Another key pillar of good data management is informed consent, which in the clinical space is well-defined by following established clinical governance practices. However, when genomic data generated for clinical purposes is re-used for research, its original consent may not be enough and re-consenting is required [137–140].

Different countries and regions can also have specific legal frameworks and guidelines pertaining to informed consent. Ideally, existing healthcare systems of record (e.g. My Health Record) can obtain and hold consent information so clinically generated data can be re-used for research.

We will focus on three consent models: (1) broad, (2) tiered, and (3) dynamic consent, all of which aim to balance the participant's control over their data with efficient data sharing[141].

Consent Models

Broad Consent

The *broad* consent model is used for studies where the genomic data collected may additionally be used in other research unrelated to the original study for which the consent was given[142,143]. This model is commonly used in large longitudinal biobanks and date from a time when it was complicated to keep contact with patients after the initial meeting. However, it poses ethical and legal challenges as individuals may not fully understand what all they are consenting to [144], in part due to the broad language used to cover future studies that have not yet been defined[145].

Tiered Consent

Unlike the broad consent model, the tiered consent model at the outset provides participants with highly specific consent options[146] . For example, participants can choose to share specific genomic information or consent to participate in specific research studies only. However, the tiered consent model creates administrative and logistical challenges for researchers to comply with the different levels of consents, and for participants to inform themselves up-front about the different options[141]. Contrasting with the one-off approach of broad consent, the tiered consent model may call for renewed consent for each new study or operate under a set of predefined conditions, dependent on the initial choices made by the participants.

Dynamic Consent

The dynamic consent model focuses on enhancing continuous engagement of participants through personalised online consent processes (e.g. *Dynamic specific consent* [141] *)* and digital communication platforms[147–150]. It is believed that dynamic consent positively influences both the recruitment and retention of participants as well as their trust towards research[147,149,151,152], while also contributing to the proficient management of the informed consent procedure[147].

Building on this, *Dynamic meta consent* enables participants to define rules to approve or reject studies without needing to decide on each study manually. For example, participants can define their preferences for data use (e.g., academic vs. commercial), data type (e.g., genomic data, medical records, imaging), research institution (e.g., universities, research labs), or funding sources (e.g., public or private)[153]. This approach provides participants with a fine-grained control over how their data is utilised yet eliminates the need to manage requests for each individual study. Akin to *tiered* consent, participants are also required to make upfront decisions at high levels of abstraction without the context for future research studies [141], however they have the flexibility to revise their decision dynamically as new information becomes available or as their preferences change.

Digital Systems for Consent Management

Traditionally, consent was obtained and recorded as a paper-based documentation. However, tiered and dynamic consent drive the adoption of digital systems where electronic consent forms enable participants to enter, manage, and withdraw their consent e.g. through web portals or mobile applications[154]. It can also allow authorised researchers/pathology providers to request access to the genomic and other health data located in the storage system for various purposes such as research or clinical decisions. However, whether data is stored in research setting or under the custodianship of an accredited pathology company influences the approaches and policies of the digital system, which are managed by three components: (1) identity and access management (IAM), (2) personalised consent elements, and (3) information storage in the context of genomics research programs.

IAM component

The IAM component manages registration and authentication, allowing authorised users (participants/patients or researchers/pathology providers) access to the system and its resources. Users are assigned an 'ID' for recognition, linking their genomic data and consent along with health records. GA4GH registered access and Passport standard can be repurposed for researchers' interaction with the digital system.

Personalised consent materials

Especially useful for genomic research programs are technologies like interactive webpages and virtual or augmented reality to *personalise consent materials* for clear and engaging explanations of complex scientific concepts and of the program's research aims to participants[155]. Language aids such as chatbots[156] and translation systems are also powerful tools to supplement this component for non-English speaking participants. An ontology system can be integrated so that consent language can be transformed into machine-readable codes that tag datasets and manage data permissions[75,157]. These elements are also helpful during consent process in a healthcare setting where the data might be used for secondary analysis.

Information storage component

As genomic research programs often handle health information as well, the *information storage component* can be integrated with data capture and management systems that comply with regulatory standards prescribed by sovereign privacy legislation such as GDPR, APP's and HIPAA to ensure that participants' data and consent are securely stored. For instance, CTRL[158], an Australian Genomics dynamic consent platform, integrates with REDCap[159] data capture system, a popular free regulatory-compliant data capture system, to collect and combine consent and research data. It should be noted that the level of security for REDCap system resides with the provider and may hence vary in quality. Other alternatives such as Castor EDC, Qualtrics, and ClinCapture are available with more user-friendly interfaces and customer support.

Digital consent management is currently delivered predominantly through centralised systems, which facilitate access control, data stewardship, and policy governance. Such centralisation brings the benefits of streamlined management and efficient consent workflows, reducing complexity for organisations. However, it imposes significant burdens on IT systems in synchronising consent changes at all levels of data usage and demands intensive manual processes to demonstrate adherence to compliance standards[160,161]. This added governance layer may inadvertently create procedural bottlenecks. These bottlenecks can lead to delays and inefficiencies that might not be directly visible to participants but could diminish their overall experience. As a result, there is risk of reduction in participant engagement and participation rate [148 162].

Decentralised approaches

Decentralised dynamic consent management systems aim to overcome the limitations of centrally managed structures by delivering both the IAM and information storage components in a programmatically insured process. This allows real-time monitoring of data use, participant-executed revocation of data, and a tamper-proof record of consent changes. It also can cater for the remote or culturally appropriate collection of

consent, such as the immutable collection of consent offline, or unfettered voting through a committee. Removing the dependency on a central authority for authorisation, reduces the risk of misconduct and misuse as auditing and strong data governance policies are baked into the approach[160,161].

Decentralised identity

Self-Sovereign Identity (SSI)[163] is a conceptual model that emphasises individuals or entity control over their digital identities, advocating for sole ownership and management. SSI facilitates a decentralised *IAM* system, where users authenticate and assert their access rights using Verifiable Credentials (VCs). VCs are digital credentials that are tamper-evident and can be verified cryptographically. This enables user identity verification while only sharing relevant information for a given context, which can enhance long-term privacy[164].

Immutable ledger technology

Distributed ledger technology (DLT), such as blockchain, can be used to deliver the *information management component* of consent management system[165,166]. DLT systems grants access to the genomic data if the data request matches the consent and complies with GDPR's right-to-be-forgotten, by detaching the ledger-based consent object from the genomic data that are stored elsewhere. It should be noted that while no identifiable information is publicly accessible, the activity of granting and revoking consent is recorded and might still reveal compromising information. The need for anonymity hence needs to be carefully balanced against the benefits from provenance of the process. Two DLT solutions for dynamic consent have been proposed, DWARNA[167] and ConsentChain[168].

DWARNA stores participants' consent in a permissioned blockchain network implemented using the stand-alone instance of Hyperledger Fabric implementation [169]. However, DWARNA is limited in treating consent state as a binary variable (broad yes/no) and, therefore, does not allow granular control over data use based on ontology-based encoding of genomic data.

ConsentChain is another proof-of-concept blockchain-based solution for managing informed consent in clinical trials. It offers more granularity compared than DWARNA by converting consent preferences into machine-readable codes using ontologies. However, ConsentChain relies on the Ethereum platform, which suffers from scalability and performance issues due to high transaction costs and low throughput. In contrast, DWARNA is built on a private blockchain and does not incur any fees for adding consent data.

Current barriers for DLT

DLT offer a secure, immutable, auditable, and transparent record of activities[170] where any modifications, such as changes to consent, are applied through a pre-agreed programmatic process rather than the approval by a central authority. However, current user-friendliness and low awareness among research and practitioners [171] hamper proof of principal applications. DLT systems are often more complex and less intuitive than centralised or federated systems and require a higher level of technical expertise and communication. The lack of compatibility between different DLT systems[172] adds to the fragmentation of the space. The Hyperledger project, seeks to overcome these challenges by providing several DLT frameworks that can be customised and integrated for various organisational needs, including healthcare and genomics research programs [61,173,174].

Current proof-of-concept dynamic consent platforms lack interoperability with both the healthcare and research systems. Embedding interoperability based on data standards such as the HL7 Fast Healthcare Interoperability Resources (FHIR) and other interchangeable standards[175,176] in a dynamic consent platform helps (i) standardise data to increase connectivity with accredited laboratory, health records and research systems, (ii) makes data accessible for participants who want to access their own data or the results of research they are involved in, and (iii) ensures compliance with the rigorous regulatory standards (like those set by the US Food and Drug Administration or The European Medicines Agency's) for submitting data or results, especially for clinical trials.

Personal data server

Offering a completely autarkic data management approach, personal data servers [177], such as SOLID PODS (Social Linked Data Personal Online Data Stores) [178,179], offer a decentralised and secure way for individuals to manage their own data, including genomic data, with control over access permissions. Individuals can choose to either establish their own server or opt to use a PODS provider like Inrupt PODS[180] providing advantages such as enhanced privacy, consent management, and better interoperability across different applications and services. However, the benefits of absolute data control in personal servers can be overshadowed by potential shortcomings, including availability issues, data corruption challenges, and the lack of guaranteed provenance, which can pose risks, especially in clinical decision-making.

Suggested framework

We envision a system that, while subject to governance and law, puts the individual at the top of the decision-making process (Figure 1). We discuss three scenarios of genomic data handling: (i) healthcare, (ii) re-analysis of health care created data (iii)

analysis of data created for research purposes, e.g. biobanks. We also suggest where various technologies could be utilised in the system such FHIR, Ontoserver, future-proof data encryption algorithms and de-centralised data storage.

In the health care context, the clinician tasks the health service provider (the Issuer) to initiate a genomic test after obtaining the patient’s consent, using a FHIR-based system that ensures traceability of the order within the health system. The system generates a verifiable credential (VC) of the patient (the Holder). This *patient* VC contains crucial patient information, including the patient identity, consent signature, and usage scope. This VC is then verified by the pathology provider (the Verifier) to create the genomic data (the Asset), whose lab-signed provenance is then attached to the VC.

As data custodians, the pathology provider encrypts the data with future-proof encryption algorithms, such as post-quantum cryptographic methods that allows only the needed genomic loci be decrypted, and only when requested by the patient. The data is then stored across decentralised nodes, further increasing obfuscation, and reducing the reliance on a single data provider.

The healthcare provider subsequently verifies the information within the received VC along with the pathology report. After verification they send the VC to the patient, who stores it securely in their personal digital wallet as proof of their genome asset and pathology report, as well as facilitating sharing and verification with other parties.

In the genetic re-analysis scenario either the healthcare provider or the researcher requests patient consent, which is verified by the pathology provider to enable data access. Researchers first need to prove their *bona fide* status as a researcher through a *researcher* VC (Figure 1, e.g. with Research Organisation as Issuer). The same holds true for pathology labs requesting access to data generated by a different lab for re-analysis; they need a VC from professional bodies that govern and attest to their validity. The decentralised dynamic consent platform enables patients to track the consents they've given for re-analysis and monitor how and where their genomic data is being used.

In the context of biobanks, they hold the role of both issuer and verifier as they initiate the data creation and coordinate the data dissemination. Researcher and other data consumers gain access after their credentials are verified (e.g. through a GA4GH Passport system) and data use terms match the participants' consent. Again, the dynamic consent platform allows the participants to stay up to date with results and use.

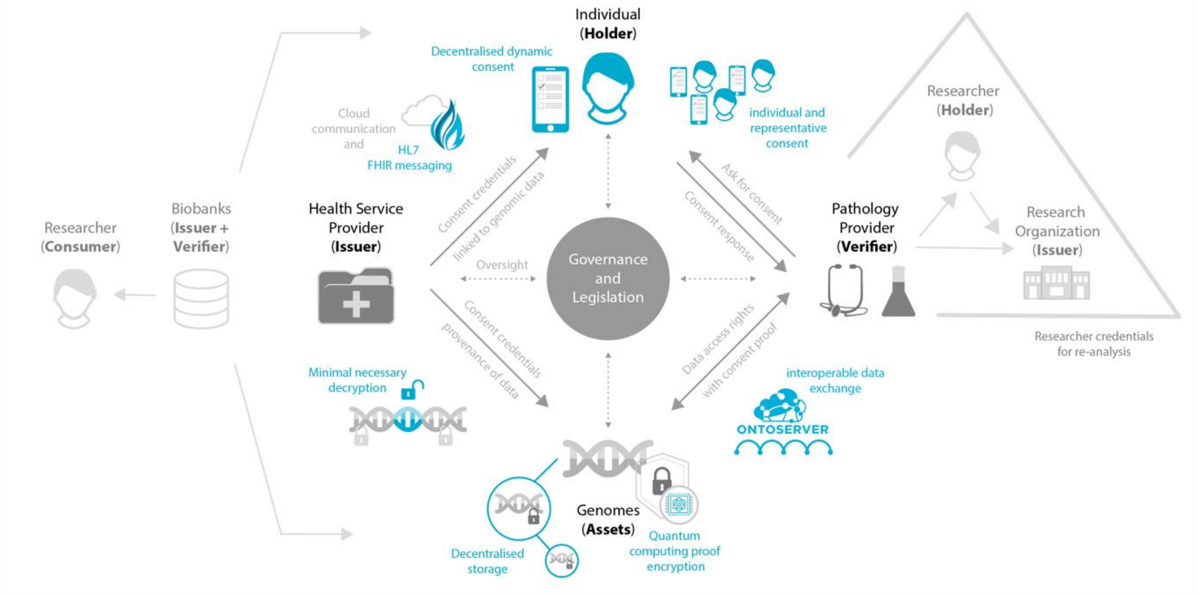

Figure 1: ***A trust model using the self-sovereign identity framework to enable participant-controlled consent management in genomics***.

Irrespective of the technology used, the sociocultural angle of genomic data management must be considered as it encompasses the public's attitudes and perspective towards genomic healthcare and research[181–183]. This social license impacts individual‘s decision to receive a genomic test and participate in research. This impacts the representation of diverse populations, including Indigenous populations, in genomic datasets, which in turn define the quality of care we can deliver to distinct populations[184].

For example, Indigenous communities often have unique cultural relationships with their genetic heritage, which can differ from the Western understanding of genetic information. As dynamic consent emphasises ongoing and flexible participation in decision-making processes, processes must account for Indigenous people's cultural values, beliefs, and protocols. Respect for Indigenous sovereignty, self-determination, and the right to control their own genetic data is crucial when developing dynamic consent frameworks. For these reasons, genetic research with Indigenous people must involve genuine consultation, inclusive participation, and informed consent processes that are culturally appropriate and respectful of Indigenous knowledge systems. Moreover, ongoing dialogue and reciprocal partnerships are essential to ensure that the benefits of genetic research are shared equitably and that potential harms, such as the unauthorised use of genetic data or exploitation, are prevented. Only by addressing these issues can dynamic consent contribute to empowering Indigenous communities to manage and protect their genetic information, foster trust, and promote ethical genomics research.

Similarly, values, beliefs, and protocols need to be respected when it comes to professional communities. For example, while clinicians support patient controlling their data, they are concerned about patients 'owning' data[15], likely due to these creating difficulties around data provenance and the ramifications of using compromised data for clinical decisions. Thus, any developed framework must keep all involved stakeholders in mind and allocate resources for appropriate communication. Specifically, the risk and benefits of genomics research, the positive impacts of data sharing, and their strong commitment and capability in protecting genomic data must be communicated to the public through educational events, online platforms, and media/social media engagement [185–189].

Finally, the legislative system needs to protect individuals against genetic discrimination and current regulatory frameworks need to evaluate decentralised and self-sovereign identity solutions. Transition towards such systems requires infrastructure remodelling, training, and education, as well as updating existing regulations. This is a resource-intensive (human and financial) process, which demands a great level of political commitment.

Conclusion

As genomic sequencing becomes cheaper and more ubiquitous, health and research organisations need to be empowered to access global data assets that are interoperable and scale easily with the application opportunities. This needs to be underpinned by the ethical and trustworthy management of genomic data[190] as the security and privacy must be balanced with the need for clinical efficiency, and ethical and safe research into population-specific care improvements.

However, this balance between protection and utility varies from circumstance to circumstance. It is hence crucial to enable individuals whose genomic data is handled to engage with the process through appropriate consent models and data governance systems. Current centralised data management strategies might get overburdened by scaling up to the level of audit trails or proof of "good processes" required to build trust with participants. Emerging decentralised data and dynamic consent management approaches have sovereignty, self-determination natively enshrined into their approaches. This enables the right to control their own data and utilise culturally-appropriate decision-making models that future participants of genomic data exchanges require.

Acknowledgement

We thank Megan Prictor, Michelle Kleynhans, John Phillips, and Jo Spencer for their critical reading of the paper. We extend our gratitude to Megan for her thought leadership that has inspired our focus areas.

Conflict of Interest

B.G. is a director of Pacific Analytics PTY LTD & SMRTR PTY LTD, Australia; his research is supported through the MRFF (Establishing epigenetic biomarkers in Indigenous Australians for precision health) and a BISA Accelerator grant.

N.T. is the Scientific Director of Genomical.

References

1. Birney, E., Vamathevan, J. & Goodhand, P. Genomics in healthcare: GA4GH looks to 2022. *BioRxiv* 203554 (2017).
2. Saunders, G. *et al.* Leveraging European infrastructures to access 1 million human genomes by 2022. *Nat Rev Genet* **20**, 693–701 (2019).
3. Goodwin, S., McPherson, J. D. & McCombie, W. R. Coming of age: ten years of next-generation sequencing technologies. *Nat Rev Genet* **17**, 333–351 (2016).
4. Schwarze, K., Buchanan, J., Taylor, J. C. & Wordsworth, S. Are whole-exome and whole-genome sequencing approaches cost-effective? A systematic review of the literature. *Genetics in Medicine* **20**, 1122–1130 (2018).
5. Li, H. *et al.* Cost-reduction strategies in massive genomics experiments. *Mar Life Sci Technol* **1**, 15–21 (2019).
6. Stoeklé, H.-C., Mamzer-Bruneel, M.-F., Vogt, G. & Hervé, C. 23andMe: a new two-sided data-banking market model. *BMC Med Ethics* **17**, 1–11 (2016).
7. Wong, E. *et al.* The Singapore National Precision Medicine Strategy. *Nat Genet* 1–9 (2023).
8. Kowal, E., Easteal, S. & Gooda, M. Indigenous genomics. *Australasian Science* **37**, 18–20 (2016).
9. Forsberg, J. S. & Soini, S. A big step for Finnish biobanking. *Nat Rev Genet* **15**, 6 (2014).
10. Leitsalu, L. *et al.* Cohort profile: Estonian biobank of the Estonian genome center, university of Tartu. *Int J Epidemiol* **44**, 1137–1147 (2015).
11. Bycroft, C. *et al.* The UK Biobank resource with deep phenotyping and genomic data. *Nature* **562**, 203–209 (2018).
12. Malakar, Y., Lacey, J., Twine, N. A. & Bauer, D. C. Applying a risk governance approach to examine how professionals perceive the benefits and risks of clinical genomics in Australian healthcare. *New Genet Soc* **42**, e2192472 (2023).
13. Cheung, R., Jolly, S., Vimal, M., Kim, H. L. & McGonigle, I. Who's afraid of genetic tests?: An assessment of Singapore's public attitudes and changes in attitudes after taking a genetic test. *BMC Med Ethics* **23**, 5 (2022).
14. Grishin, D. *et al.* Citizen-centered, auditable and privacy-preserving population genomics. *Nat Comput Sci* **1**, 192–198 (2021).
15. Malakar, Y., Lacey, J., Twine, N. A., McCrea, R. & Bauer, D. C. Balancing the safeguarding of privacy and data sharing: perceptions of genomic professionals on patient genomic data ownership in Australia. *European Journal of Human Genetics* 1–7 (2023).
16. Gymrek, M., McGuire, A. L., Golan, D., Halperin, E. & Erlich, Y. Identifying personal genomes by surname inference. *Science (1979)* **339**, 321–324 (2013).
17. Erlich, Y. *et al.* Redefining genomic privacy: trust and empowerment. *PLoS Biol* **12**, e1001983 (2014).
18. Von Thenen, N., Ayday, E. & Cicek, A. E. Re-identification of individuals in genomic data-sharing beacons via allele inference. *Bioinformatics* **35**, 365–371 (2019).
19. Raisaro, J. L. *et al.* Addressing Beacon re-identification attacks: quantification and mitigation of privacy risks. *Journal of the American Medical Informatics Association* **24**, 799–805 (2017).
20. Shringarpure, S. S. & Bustamante, C. D. Privacy risks from genomic data-sharing beacons. *The American Journal of Human Genetics* **97**, 631–646 (2015).
21. Gürsoy, G. Genome Privacy and Trust. *Annu Rev Biomed Data Sci* **5**, 163–181 (2022).

22. Venkatesaramani, R., Malin, B. A. & Vorobeychik, Y. Re-identification of individuals in genomic datasets using public face images. *Sci Adv* **7**, eabg3296 (2021).
23. Lippert, C. *et al.* Identification of individuals by trait prediction using whole-genome sequencing data. *Proceedings of the National Academy of Sciences* **114**, 10166–10171 (2017).
24. Lippert, C. *et al.* Identification of individuals by trait prediction using whole-genome sequencing data. *Proceedings of the National Academy of Sciences* **114**, 10166–10171 (2017).
25. Lee, S. S. Excavating the personal genome: The good biocitizen in the age of precision health. *Hastings Center Report* **50**, S54–S61 (2020).
26. Bullen, J. *et al.* From Deficit to Strength-Based Aboriginal Health Research—Moving toward Flourishing. *Int J Environ Res Public Health* **20**, 5395 (2023).
27. U.S. Dept. of Health and Human Services. Standards for privacy of individually identifiable health information. *Federal Registrar2002; 45 CFR, Parts 160–4.* (2002).
28. The California Consumer Privacy Act of 2018.
29. Paltiel, M., Taylor, M. & Newson, A. Protection of genomic data and the Australian Privacy Act: when are genomic data 'personal information'? *International Data Privacy Law* **13**, 47–62 (2023).
30. Regulation (EU) 2016/679 , General Data Protection Regulation (GDPR). *OJ L 119, 4.5.2016, p. 1–88* (2016).
31. Tiller, J. *et al.* Community concerns about genetic discrimination in life insurance persist in Australia: A survey of consumers offered genetic testing. *European Journal of Human Genetics* 1–9 (2023).
32. Seh, A. H. *et al.* Healthcare Data Breaches: Insights and Implications. *Healthcare* **8**, 133 (2020).
33. Pandey, A. K. *et al.* Key Issues in Healthcare Data Integrity: Analysis and Recommendations. *IEEE Access* **8**, 40612–40628 (2020).
34. Abinaya B. & Santhi S. A survey on genomic data by privacy-preserving techniques perspective. *Comput Biol Chem* **93**, 107538 (2021).
35. Pulivarti, R. *et al.* Cybersecurity of Genomic Data. *Gaithersburg, MD: National Institute of Standards and Technology* (2023).
36. Regulation (EU) 2016/679 , General Data Protection Regulation (GDPR). *OJ L 119, 4.5.2016, p. 1–88* (2016).
37. Olson, J. S. & Olson, G. *Working Together Apart: Collaboration over the Internet*. (Springer Nature, 2022).
38. Golec, D., Strugar, I. & Belak, D. The Benefits of Enterprise Data Warehouse Implementation in Cloud vs. On-premises. *ENTRENOVA - ENTerprise REsearch InNOVAtion* **7**, 67–76 (2021).
39. Wu, J., Ping, L., Ge, X., Wang, Y. & Fu, J. Cloud Storage as the Infrastructure of Cloud Computing. in *2010 International Conference on Intelligent Computing and Cognitive Informatics* 380–383 (IEEE, 2010). doi:10.1109/ICICCI.2010.119.
40. Chee, B. J. S. & Franklin Jr, C. *Cloud Computing: Technologies and Strategies of the Ubiquitous Data Center*. (CRC Press, 2010).
41. Chorafas, D. N. *Cloud Computing Strategies*. (CRC press, 2010).
42. AWS GovCloud (US) Region – Government Cloud Computing. At: http://aws.amazon.com/govcloud-us/.
43. Microsoft AZURE Government. AT https://azure.microsoft.com/en-us/explore/global-infrastructure/government.
44. B. K. Reddy, B. T. Rao, Dr.L.S.S. Reddy & P. Kiran. Issues in Cloud Computing. *Global Journal of Computer Science and Technology* **11**, (2011).

45. Ghani, A., Badshah, A., Jan, S., Alshdadi, A. A. & Daud, A. Issues and challenges in Cloud Storage Architecture: A Survey. (2020).
46. Prajapati, P. & Shah, P. A Review on Secure Data Deduplication: Cloud Storage Security Issue. *Journal of King Saud University - Computer and Information Sciences* **34**, 3996–4007 (2022).
47. Odun-Ayo, I., Ajayi, O., Akanle, B. & Ahuja, R. An Overview of Data Storage in Cloud Computing. in *2017 International Conference on Next Generation Computing and Information Systems (ICNGCIS)* 29–34 (IEEE, 2017). doi:10.1109/ICNGCIS.2017.9.
48. Khan, A. R. & Alnwihel, L. K. A Brief Review on Cloud Computing Authentication Frameworks. *Engineering, Technology & Applied Science Research* **13**, 9997–10004 (2023).
49. Rajani, S., Ghorpade, V. & Dhange, M. Multi-factor authentication as a service for cloud data security. *Int J Comput Sci Eng* **4**, 43–46 (2016).
50. AWS multi-factor authentication (MFA). AT https://aws.amazon.com/what-is/mfa/.
51. Microsoft AZURE MFA. AT https://www.microsoft.com/en-au/security/business/identity-access/microsoft-entra-mfa-multi-factor-authentication.
52. Deflaux, N. *et al.* Demonstrating paths for unlocking the value of cloud genomics through cross cohort analysis. *Nat Commun* **14**, 5419 (2023).
53. Thorne, N. *et al.* Genovic: a secure, scalable and modular system for genomic testing. *Pathology* **51**, S120–S121 (2019).
54. Wong, M. *et al.* Whole genome, transcriptome and methylome profiling enhances actionable target discovery in high-risk pediatric cancer. *Nat Med* **26**, 1742–1753 (2020).
55. Huston, G. AARNet. in *The User's Directory of Computer Networks* 199–201 (Elsevier, 1990). doi:10.1016/B978-1-55558-047-6.50020-5.
56. Murray, A., Kim, D. & Combs, J. The promise of a decentralized internet: What is Web3 and how can firms prepare? *Bus Horiz* **66**, 191–202 (2023).
57. Sharma, P., Jindal, R. & Borah, M. D. Blockchain-based decentralized architecture for cloud storage system. *Journal of Information Security and Applications* **62**, 102970 (2021).
58. Benet, J. IPFS - Content Addressed, Versioned, P2P File System. (2014).
59. Mani, V., Manickam, P., Alotaibi, Y., Alghamdi, S. & Khalaf, O. I. Hyperledger Healthchain: Patient-Centric IPFS-Based Storage of Health Records. *Electronics (Basel)* **10**, 3003 (2021).
60. Azbeg, K., Ouchetto, O. & Jai Andaloussi, S. BlockMedCare: A healthcare system based on IoT, Blockchain and IPFS for data management security. *Egyptian Informatics Journal* **23**, 329–343 (2022).
61. Kumar, R., Marchang, N. & Tripathi, R. Distributed Off-Chain Storage of Patient Diagnostic Reports in Healthcare System Using IPFS and Blockchain. in *2020 International Conference on COMmunication Systems & NETworkS (COMSNETS)* 1–5 (IEEE, 2020). doi:10.1109/COMSNETS48256.2020.9027313.
62. Mackey, T. K. *et al.* Establishing a blockchain-enabled Indigenous data sovereignty framework for genomic data. *Cell* **185**, 2626–2631 (2022).
63. Filecoin: A Decentralized Market for Storage. *[online] Available: https://filecoin.io.*
64. Liebau, D. & Schueffel, P. Crypto-Currencies and ICOs: Are They Scams? An Empirical Study. *SSRN Electronic Journal* (2019) doi:10.2139/ssrn.3320884.
65. Tiwari, M., Gepp, A. & Kumar, K. The future of raising finance - a new opportunity to commit fraud: a review of initial coin offering (ICOs) scams. *Crime Law Soc Change* **73**, 417–441 (2020).

66. Aminzade, M. Confidentiality, integrity and availability–finding a balanced IT framework. *Network Security* **2018**, 9–11 (2018).
67. Cheah, P. Y. & Piasecki, J. Data Access Committees. *BMC Med Ethics* **21**, 12 (2020).
68. Dyke, S. O. M. Genomic data access policy models. in *Responsible Genomic Data Sharing* 19–32 (Elsevier, 2020).
69. Dyke, S. O. M. *et al.* Registered access: authorizing data access. *European Journal of Human Genetics* **26**, 1721–1731 (2018).
70. Rehm, H. L. *et al.* GA4GH: International policies and standards for data sharing across genomic research and healthcare. *Cell Genomics* **1**, 100029 (2021).
71. Dyke, S. O. M. *et al.* Registered access: a 'Triple-A'approach. *European Journal of Human Genetics* **24**, 1676–1680 (2016).
72. Voisin, C. *et al.* GA4GH Passport standard for digital identity and access permissions. *Cell Genomics* **1**, 100030 (2021).
73. Sakimura, N., Bradley, J., Jones, M., De Medeiros, B. & Mortimore, C. Openid connect core 1.0. *The OpenID Foundation* S3 (2014).
74. Hardt, D. *The OAuth 2.0 Authorization Framework*. (2012).
75. Lawson, J. *et al.* The Data Use Ontology to streamline responsible access to human biomedical datasets. *Cell Genomics* **1**, 100028 (2021).
76. Debnath, S., Chattopadhyay, A. & Dutta, S. Brief review on journey of secured hash algorithms. in *2017 4th International Conference on Opto-Electronics and Applied Optics (Optronix)* 1–5 (IEEE, 2017).
77. Preneel, B., Govaerts, R. & Vandewalle, J. Cryptographic hash functions: an overview. in *Proceedings of the 6th international computer security and virus conference (ICSVC 1993)* vol. 19 (1993).
78. Stinson, D. R. Some observations on the theory of cryptographic hash functions. *Des Codes Cryptogr* **38**, 259–277 (2006).
79. Ayday, E., Tang, Q. & Yilmaz, A. Cryptographic solutions for credibility and liability issues of genomic data. *IEEE Trans Dependable Secure Comput* **16**, 33–43 (2017).
80. Sweeney, L. k-anonymity: A model for protecting privacy. *International journal of uncertainty, fuzziness and knowledge-based systems* **10**, 557–570 (2002).
81. Rajendran, K., Jayabalan, M. & Rana, M. E. A study on k-anonymity, l-diversity, and t-closeness techniques. *IJCSNS* **17**, 172 (2017).
82. Malin, B. A. Protecting genomic sequence anonymity with generalization lattices. *Methods Inf Med* **44**, 687–692 (2005).
83. Humbert, M., Ayday, E., Hubaux, J.-P. & Telenti, A. Reconciling utility with privacy in genomics. in *Proceedings of the 13th Workshop on Privacy in the Electronic Society* 11–20 (2014).
84. Bonomi, L., Huang, Y. & Ohno-Machado, L. Privacy challenges and research opportunities for genomic data sharing. *Nat Genet* **52**, 646–654 (2020).
85. Hekel, R. *et al.* Privacy-preserving storage of sequenced genomic data. *BMC Genomics* **22**, 1–13 (2021).
86. Aggarwal, C. C. On k-anonymity and the curse of dimensionality. in *VLDB* vol. 5 901–909 (2005).
87. Dwork, C. & Roth, A. The algorithmic foundations of differential privacy. *Foundations and Trends® in Theoretical Computer Science* **9**, 211–407 (2014).
88. Rambla, J. *et al.* Beacon v2 and beacon networks: a "lingua franca" for federated data discovery in biomedical genomics, and beyond. *Hum Mutat* **43**, 791–799 (2022).
89. Aziz, M. M. Al, Ghasemi, R., Waliullah, M. & Mohammed, N. Aftermath of bustamante attack on genomic beacon service. *BMC Med Genomics* **10**, 43–54 (2017).

90. Yan, J., Han, Z., Zhou, Y. & Lu, L. A Differential Privacy Approach to Preserve GWAS Data Sharing based on A Game Theoretic Perspective. *KSII Transactions on Internet and Information Systems (TIIS)* **16**, 1028–1046 (2022).
91. Simmons, S., Sahinalp, C. & Berger, B. Enabling privacy-preserving GWASs in heterogeneous human populations. *Cell Syst* **3**, 54–61 (2016).
92. Almadhoun, N., Ayday, E. & Ulusoy, Ö. Differential privacy under dependent tuples—the case of genomic privacy. *Bioinformatics* **36**, 1696–1703 (2020).
93. Makarious, M. B. *et al.* GenoML: automated machine learning for genomics. *arXiv preprint arXiv:2103.03221* (2021).
94. Rieke, N. *et al.* The future of digital health with federated learning. *NPJ Digit Med* **3**, 119 (2020).
95. Boscarino, N., Cartwright, R. A., Fox, K. & Tsosie, K. S. Federated learning and Indigenous genomic data sovereignty. *Nat Mach Intell* 1–3 (2022).
96. Mocanu, I., Smadu, R., Dragoi, M., Mocanu, A. & Cramariuc, O. Testing Federated Learning on Health and Wellbeing Data. in *2021 International Conference on e-Health and Bioengineering (EHB)* 1–4 (IEEE, 2021).
97. Xu, J. *et al.* Federated learning for healthcare informatics. *J Healthc Inform Res* **5**, 1–19 (2021).
98. Kolobkov, D. *et al.* Efficacy of federated learning on genomic data: a study on the UK Biobank and the 1000 Genomes Project. *medRxiv* 2021–2023 (2023).
99. Gosselin, R., Vieu, L., Loukil, F. & Benoit, A. Privacy and Security in Federated Learning: A Survey. *Applied Sciences* **12**, 9901 (2022).
100. Li, N. & Stephens, M. Modeling linkage disequilibrium and identifying recombination hotspots using single-nucleotide polymorphism data. *Genetics* **165**, 2213–2233 (2003).
101. Su, Z., Marchini, J. & Donnelly, P. HAPGEN2: simulation of multiple disease SNPs. *Bioinformatics* **27**, 2304–2305 (2011).
102. Baumdicker, F. *et al.* Efficient ancestry and mutation simulation with msprime 1.0. *Genetics* **220**, iyab229 (2022).
103. Yelmen, B. *et al.* Creating artificial human genomes using generative neural networks. *PLoS Genet* **17**, e1009303 (2021).
104. Atkinson, E. G. *et al.* Cross-ancestry genomic research: time to close the gap. *Neuropsychopharmacology* **47**, 1737–1738 (2022).
105. Oprisanu, B., Ganev, G. & De Cristofaro, E. On utility and privacy in synthetic genomic data. *arXiv preprint arXiv:2102.03314* (2021).
106. Stadler, T., Oprisanu, B. & Troncoso, C. Synthetic data-A privacy mirage. *arXiv preprint arXiv:2011.07018* (2020).
107. Chandra, S., Bhattacharyya, S., Paira, S. & Alam, S. S. A study and analysis on symmetric cryptography. in *2014 International Conference on Science Engineering and Management Research (ICSEMR)* 1–8 (IEEE, 2014).
108. Patel, K. Performance analysis of AES, DES and Blowfish cryptographic algorithms on small and large data files. *International Journal of Information Technology* **11**, 813–819 (2019).
109. Qadir, A. M. & Varol, N. A review paper on cryptography. in *2019 7th international symposium on digital forensics and security (ISDFS)* 1–6 (IEEE, 2019).
110. Jiao, L., Hao, Y. & Feng, D. Stream cipher designs: a review. *Science China Information Sciences* **63**, 1–25 (2020).
111. Serrano, R., Duran, C., Sarmiento, M., Pham, C.-K. & Hoang, T.-T. ChaCha20–Poly1305 Authenticated Encryption with Additional Data for Transport Layer Security 1.3. *Cryptography* **6**, 30 (2022).

112. Senf, A. *et al.* Crypt4GH: a file format standard enabling native access to encrypted data. *Bioinformatics* **37**, 2753–2754 (2021).
113. Hosseini, M., Pratas, D. & Pinho, A. J. Cryfa: a secure encryption tool for genomic data. *Bioinformatics* **35**, 146–148 (2019).
114. Lei, X., Zhu, X., Chi, H. & Jiang, S. Cloud-assisted privacy-preserving genetic paternity test. in *2015 IEEE/CIC International Conference on Communications in China (ICCC)* 1–6 (IEEE, 2015).
115. Kalra, S. & Sood, S. K. Elliptic curve cryptography: survey and its security applications. in *Proceedings of the international conference on advances in computing and artificial intelligence* 102–106 (2011).
116. Jiang, Y., Shang, T. & Liu, J. SM algorithms-based encryption scheme for large genomic data files. *Digital Communications and Networks* **7**, 543–550 (2021).
117. Keller, M. MP-SPDZ: A versatile framework for multi-party computation. in *Proceedings of the 2020 ACM SIGSAC conference on computer and communications security* 1575–1590 (2020).
118. Yao, A. C. Protocols for secure computations. in *23rd annual symposium on foundations of computer science (sfcs 1982)* 160–164 (IEEE, 1982).
119. Bogdanov, D. *et al.* Privacy-preserving statistical data analysis on federated databases. in *Privacy Technologies and Policy: Second Annual Privacy Forum, APF 2014, Athens, Greece, May 20-21, 2014. Proceedings 2* 30–55 (Springer, 2014).
120. Xie, W. *et al.* SecureMA: protecting participant privacy in genetic association meta-analysis. *Bioinformatics* **30**, 3334–3341 (2014).
121. Jagadeesh, K. A., Wu, D. J., Birgmeier, J. A., Boneh, D. & Bejerano, G. Deriving genomic diagnoses without revealing patient genomes. *Science (1979)* **357**, 692–695 (2017).
122. Lauter, K., López-Alt, A. & Naehrig, M. Private computation on encrypted genomic data. in *Progress in Cryptology-LATINCRYPT 2014: Third International Conference on Cryptology and Information Security in Latin America Florianópolis, Brazil, September 17–19, 2014 Revised Selected Papers* 3–27 (Springer, 2015).
123. Kachouh, B., Hariss, K., Sliman, L., Samhat, A. E. & Alsuliman, T. Privacy preservation of genome data analysis using homomorphic encryption. *Service Oriented Computing and Applications* **15**, 273–287 (2021).
124. Gürsoy, G., Chielle, E., Brannon, C. M., Maniatakos, M. & Gerstein, M. Privacy-preserving genotype imputation with fully homomorphic encryption. *Cell Syst* **13**, 173–182 (2022).
125. Blatt, M., Gusev, A., Polyakov, Y. & Goldwasser, S. Secure large-scale genome-wide association studies using homomorphic encryption. *Proceedings of the National Academy of Sciences* **117**, 11608–11613 (2020).
126. Titus, A. J. *et al.* SIG-DB: Leveraging homomorphic encryption to securely interrogate privately held genomic databases. *PLoS Comput Biol* **14**, e1006454 (2018).
127. Thomson, I. Microsoft researchers smash homomorphic encryption speed barrier. *The Register* (2016).
128. Raisaro, J. L. *et al.* Protecting privacy and security of genomic data in i2b2 with homomorphic encryption and differential privacy. *IEEE/ACM Trans Comput Biol Bioinform* **15**, 1413–1426 (2018).
129. Wu, H. & Wang, F. A survey of noninteractive zero knowledge proof system and its applications. *The Scientific World Journal* **2014**, (2014).
130. Hwang, S., Ozturk, E. & Tsudik, G. Balancing Security and Privacy in Genomic Range Queries. *ACM Transactions on Privacy and Security* **26**, 1–28 (2023).

131. Yang, Y. *et al.* PriGenX: Privacy-preserving Query With Anonymous Access Control for Genomic Data. *IEEE Trans Dependable Secure Comput* (2023).
132. Shor, P. W. Algorithms for quantum computation: discrete logarithms and factoring. in *Proceedings 35th annual symposium on foundations of computer science* 124–134 (Ieee, 1994).
133. O'Neill, P. H. The US is worried that hackers are stealing data today so quantum computers can crack it in a decade. Preprint at (2022).
134. Avanzi, R. *et al.* CRYSTALS-Kyber algorithm specifications and supporting documentation. *NIST PQC Round* **2**, 1–43 (2019).
135. Ducas, L. *et al.* Crystals-dilithium: A lattice-based digital signature scheme. *IACR Transactions on Cryptographic Hardware and Embedded Systems* 238–268 (2018).
136. Soni, D. *et al.* FALCON. *Hardware Architectures for Post-Quantum Digital Signature Schemes* 31–41 (2021).
137. McGuire, A. L. & Beskow, L. M. Informed consent in genomics and genetic research. *Annu Rev Genomics Hum Genet* **11**, 361–381 (2010).
138. Declaration de Helsinki, A. M. M. Ethical Principles for Medical Research Involving Human Subjects. *Recuperado de: http://www. wma. net/es/30publications/10policies/b3* (2013).
139. Ten Have, H. & Jean, M. *The UNESCO Universal Declaration on Bioethics and Human Rights: Background, Principles and Application*. (Unesco, 2009).
140. Koplin, J. J., Gyngell, C., Savulescu, J. & Vears, D. F. Moving from 'fully'to 'appropriately'informed consent in genomics: The PROMICE framework. *Bioethics* **36**, 655–665 (2022).
141. Wiertz, S. & Boldt, J. Evaluating models of consent in changing health research environments. *Med Health Care Philos* **25**, 269–280 (2022).
142. Mikkelsen, R. B., Gjerris, M., Waldemar, G. & Sandøe, P. Broad consent for biobanks is best–provided it is also deep. *BMC Med Ethics* **20**, 1–12 (2019).
143. Tindana, P. & de Vries, J. Broad consent for genomic research and biobanking: perspectives from low-and middle-income countries. *Annu Rev Genomics Hum Genet* **17**, 375–393 (2016).
144. Barazzetti, G., Bosisio, F., Koutaissoff, D. & Spencer, B. Broad consent in practice: lessons learned from a hospital-based biobank for prospective research on genomic and medical data. *European Journal of Human Genetics* **28**, 915–924 (2020).
145. Mikkelsen, R. B., Gjerris, M., Waldemar, G. & Sandøe, P. Broad consent for biobanks is best–provided it is also deep. *BMC Med Ethics* **20**, 1–12 (2019).
146. Bunnik, E. M., Janssens, A. C. J. W. & Schermer, M. H. N. A tiered-layered-staged model for informed consent in personal genome testing. *European Journal of Human Genetics* **21**, 596–601 (2013).
147. Mascalzoni, D. *et al.* Ten years of dynamic consent in the CHRIS study: informed consent as a dynamic process. *European Journal of Human Genetics* **30**, 1391–1397 (2022).
148. Budin-Ljøsne, I. *et al.* Dynamic consent: a potential solution to some of the challenges of modern biomedical research. *BMC Med Ethics* **18**, 1–10 (2017).
149. Teare, H. J. A., Prictor, M. & Kaye, J. Reflections on dynamic consent in biomedical research: the story so far. *European journal of human genetics* **29**, 649–656 (2021).
150. Teare, H. J. A., Morrison, M., Whitley, E. A. & Kaye, J. Towards 'Engagement 2.0': Insights from a study of dynamic consent with biobank participants. *Digit Health* **1**, 2055207615605644 (2015).
151. Haas, M. A., Madelli, E. O., Brown, R., Prictor, M. & Boughtwood, T. Evaluation of CTRL: a web application for dynamic consent and engagement with individuals

involved in a cardiovascular genetic disorders cohort. *European Journal of Human Genetics* 1–8 (2023).

152. Spencer, K. *et al.* Patient perspectives on sharing anonymized personal health data using a digital system for dynamic consent and research feedback: a qualitative study. *J Med Internet Res* **18**, e5011 (2016).
153. Budin-Ljøsne, I., Teare, H., Kaye, J. & Mascalzoni, D. Meta consent: Is it new and is it fit for purpose? *BMJ* **350**, (2016).
154. Chen, C. *et al.* Replacing paper informed consent with electronic informed consent for research in academic medical centers: a scoping review. *AMIA Summits on Translational Science Proceedings* **2020**, 80 (2020).
155. Mishra, R. *et al.* Virtual reality in neurosurgery: beyond neurosurgical planning. *Int J Environ Res Public Health* **19**, 1719 (2022).
156. Xiao, Z., Li, T. W., Karahalios, K. & Sundaram, H. Inform the Uninformed: Improving Online Informed Consent Reading with an AI-Powered Chatbot. in *Proceedings of the 2023 CHI Conference on Human Factors in Computing Systems* 1–17 (2023).
157. Metke-Jimenez, A., Steel, J., Hansen, D. & Lawley, M. Ontoserver: a syndicated terminology server. *J Biomed Semantics* **9**, 1–10 (2018).
158. Haas, M. A. *et al.* ‘CTRL’: an online, Dynamic Consent and participant engagement platform working towards solving the complexities of consent in genomic research. *European Journal of Human Genetics* **29**, 687–698 (2021).
159. Harvey, L. A. REDCap: web-based software for all types of data storage and collection. *Spinal Cord* **56**, 625 (2018).
160. Mackey, T. K. *et al.* Establishing a blockchain-enabled Indigenous data sovereignty framework for genomic data. *Cell* **185**, 2626–2631 (2022).
161. Zichichi, M., Ferretti, S., D’Angelo, G. & Rodríguez-Doncel, V. Data governance through a multi-dlt architecture in view of the gdpr. *Cluster Comput* **25**, 4515–4542 (2022).
162. Prictor, M., Teare, H. J. A. & Kaye, J. Equitable participation in biobanks: the risks and benefits of a “dynamic consent” approach. *Front Public Health* **6**, 253 (2018).
163. Preukschat, A. & Reed, D. *Self-Sovereign Identity*. (Manning Publications, 2021).
164. Naik, N. & Jenkins, P. Governing principles of self-sovereign identity applied to blockchain enabled privacy preserving identity management systems. in *2020 IEEE International Symposium on Systems Engineering (ISSE)* 1–6 (IEEE, 2020).
165. Román-Martínez, I. *et al.* Blockchain-Based Service-Oriented Architecture for Consent Management, Access Control, and Auditing. *IEEE Access* **11**, 12727–12741 (2023).
166. Thiebes, S., Schlesner, M., Brors, B. & Sunyaev, A. Distributed ledger technology in genomics: a call for Europe. *European Journal of Human Genetics* **28**, 139–140 (2020).
167. Mamo, N., Martin, G. M., Desira, M., Ellul, B. & Ebejer, J.-P. Dwarna: a blockchain solution for dynamic consent in biobanking. *European Journal of Human Genetics* **28**, 609–626 (2020).
168. Albalwy, F., Brass, A. & Davies, A. A blockchain-based dynamic consent architecture to support clinical genomic data sharing (ConsentChain): Proof-of-concept study. *JMIR Med Inform* **9**, e27816 (2021).
169. Androulaki, E. *et al.* Hyperledger fabric: a distributed operating system for permissioned blockchains. in *Proceedings of the thirteenth EuroSys conference* 1–15 (2018).

170. Sunyaev, A. & Sunyaev, A. Distributed ledger technology. *Internet computing: Principles of distributed systems and emerging internet-based technologies* 265–299 (2020).
171. Alghazwi, M., Turkmen, F., Van Der Velde, J. & Karastoyanova, D. Blockchain for genomics: a systematic literature review. *Distributed Ledger Technologies: Research and Practice* **1**, 1–28 (2022).
172. Belchior, R., Vasconcelos, A., Guerreiro, S. & Correia, M. A survey on blockchain interoperability: Past, present, and future trends. *ACM Computing Surveys (CSUR)* **54**, 1–41 (2021).
173. Beyene, M. *et al.* A scoping review of distributed ledger technology in genomics: thematic analysis and directions for future research. *Journal of the American Medical Informatics Association* **29**, 1433–1444 (2022).
174. Alghazwi, M., Turkmen, F., Van Der Velde, J. & Karastoyanova, D. Blockchain for genomics: a systematic literature review. *Distributed Ledger Technologies: Research and Practice* **1**, 1–28 (2022).
175. Vorisek, C. N. *et al.* Fast Healthcare Interoperability Resources (FHIR) for Clinical, Epidemiological and Public Health Research: A Systematic Review. *Epidemiological and Public Health Research: A Systematic Review*.
176. Bönisch, C., Kesztyüs, D. & Kesztyüs, T. Harvesting metadata in clinical care: a crosswalk between FHIR, OMOP, CDISC and openEHR metadata. *Sci Data* **9**, 659 (2022).
177. Verbrugge, S. *et al.* Towards a personal data vault society: an interplay between technological and business perspectives. in *2021 60th FITCE Communication Days Congress for ICT Professionals: Industrial Data–Cloud, Low Latency and Privacy (FITCE)* 1–6 (IEEE, 2021).
178. Sambra, A. V. *et al.* Solid: a platform for decentralized social applications based on linked data. *MIT CSAIL & Qatar Computing Research Institute, Tech. Rep.* (2016).
179. Werbrouck, J., Pauwels, P., Beetz, J. & van Berlo, L. Towards a decentralised common data environment using linked building data and the solid ecosystem. in *36th CIB W78 2019 Conference* 113–123 (2019).
180. Inrupt Solid pods. (2023).
181. Bíró, K. *et al.* Investigating the knowledge of and public attitudes towards genetic testing within the Visegrad countries: a cross-sectional study. *BMC Public Health* **20**, 1–10 (2020).
182. Zhong, A. *et al.* Ethical, social, and cultural issues related to clinical genetic testing and counseling in low-and middle-income countries: a systematic review. *Genetics in Medicine* **23**, 2270–2280 (2021).
183. Jonassaint, C. R. *et al.* Regional differences in awareness and attitudes regarding genetic testing for disease risk and ancestry. *Hum Genet* **128**, 249–260 (2010).
184. Garrison, N. *et al.* Genomic research through an indigenous lens: understanding the expectations. *Annu Rev Genomics Hum Genet* **20**, 495–517 (2019).
185. Giroux, C. M. & Moreau, K. A. Leveraging social media for medical education: Learning from patients in online spaces. *Med Teach* **42**, 970–972 (2020).
186. Talwar, D., Tseng, T.-S., Foster, M., Xu, L. & Chen, L.-S. Genetics/genomics education for nongenetic health professionals: a systematic literature review. *Genetics in medicine* **19**, 725–732 (2017).
187. Bennett, R. L., Waggoner, D. & Blitzer, M. G. Medical genetics and genomics education: how do we define success? Where do we focus our resources? *Genetics in Medicine* **19**, 751–753 (2017).

188. Stellefson, M., Paige, S. R., Chaney, B. H. & Chaney, J. D. Evolving role of social media in health promotion: updated responsibilities for health education specialists. *Int J Environ Res Public Health* **17**, 1153 (2020).
189. Kawasaki, H., Kawasaki, M., Iki, T. & Matsuyama, R. Genetics education program to help public health nurses improve their knowledge and enhance communities' genetic literacy: A pilot study. *BMC Nurs* **20**, 1–13 (2021).
190. Aarestrup, F. M. *et al.* Towards a European health research and innovation cloud (HRIC). *Genome Med* **12**, 1–14 (2020).

Difference to previous version

Click here to access/download
Supplementary Material
Dynamic_consent__review1_review2_diff.docx

Personal Cover Click here to access/download;Personal Cover;Response to reviewer R2_Feb_07_2024.docx

Dear Dr. Nogoy,

We are grateful for the guidance provided during the second round of review of our manuscript. Below, we have responded to the specific comments of reviewer 2.

We also used the opportunity to add more information about how GA4GH passports link in with the OAuth 2.0 framework, a central building block of modern web exchanges. The new section reads as follows:

To support automation and interoperability across multiple data systems, the GA4GH has developed the GA4GH Passports and Authentication & Authorisation Infrastructure (AAI) specifications[73]. Passports provide the format for data and resource access permissions, built on top of the openID Connect standards[74], which in turn are based on the OAuth 2.0 framework[75]. They serve as AAI tokens (utilising JSON Web Tokens) to carry a researcher's digital identity and access rights across organisations, tools, environments, encoding each permission/claim as "Visas", allowing access to specific registered-access datasets. Visas are issued if the intended use of the data complies with restrictions set out by the DAC. Uses and restrictions are based on defined terms in the GA4GH Data Use Ontology (DUO) [76], fully automating the whole process, potentially saving months between an access request and approval.

Reviewer #2:

1) *My assessment is that this paper still has issues with its framing and structure. The work lacks clarity in its scope - while you have addressed some of the writing prompts in the reviewer comments, you have not engaged with any substantive suggestions.*

RESPONSE: While we have not converted the document into a systematic review as suggested by the reviewer, we outlined our reasons for why we feel a literature review is the more suitable format.

2) *The reference to Genomical without any discussion of the market it sits in or competitors in this space is problematic. There is no evidence provided to support the claims made about this company, e.g., a relevant white paper or research paper with statistics, or linking the organization's approach to scientific development or theory.*

RESPONSE: We have removed references to the company, and instead referred to their peer-reviewed technology as developed under the public funding of the Australian and Melbourne Genomics Health Alliance, supported by the Victorian Government. The new paragraph reads as following:

For example, Melbourne Genomics Health Alliance, a collaboration of leading Victorian hospitals, research, and academic institutions working with the Victorian Government to embed genomics into the health system, developed a cloud solution

that benefits from international developments in security, scalability, and health system interoperability, yet seeks to minimise third-party dependence by implementing federated data governance controls. Specifically, the genomic information management system, Genomical (previously known as GenoVic[53]) offers a robust clinical data governance framework and implemented capability for controlled access and reuse of data between authorised entities for the purpose of clinical care, therefore, providing foundations for data reuse within the healthcare system where it is adopted.

53. Thorne, N. et al. Genovic: a secure, scalable and modular system for genomic testing. Pathology 51, S120–S121 (2019).

3) *The discussion of dynamic consent is based on theory papers that themselves suppose advantages of the model, rather than make claims based in empirical data. Claims are made here without either a) a critical analysis of the literature, or b) evidence to support or refute the claim made.*

RESPONSE: We have expanded the theoretical papers on dynamic consent with papers outlining the evidence for dynamic consent. The new section reads as follows and refers to these citations:

The dynamic consent model focuses on enhancing continuous engagement of participants through personalised online consent processes (e.g. *Dynamic specific consent* [142] *)* and digital communication platforms[148–151]. It is believed that dynamic consent positively influences both the recruitment and retention of participants as well as their trust towards research[148,150,152,153], while also contributing to the proficient management of the informed consent procedure[148].

148. Mascalzoni, D. et al. Ten years of dynamic consent in the CHRIS study: informed consent as a dynamic process. European Journal of Human Genetics ***30****, 1391–1397 (2022).*
151. Teare, H. J. A., Prictor, M. & Kaye, J. Reflections on dynamic consent in biomedical research: the story so far. European journal of human genetics ***29****, 649–656 (2021).*
152. Haas, M. A., Madelli, E. O., Brown, R., Prictor, M. & Boughtwood, T. Evaluation of CTRL: a web application for dynamic consent and engagement with individuals involved in a cardiovascular genetic disorders cohort. European Journal of Human Genetics 1–8 (2023).
153. Spencer, K. et al. Patient perspectives on sharing anonymized personal health data using a digital system for dynamic consent and research feedback: a qualitative study. J Med Internet Res ***18****, e5011 (2016).*

4) *I congratulate you on the framework you have produced, but it is unclear as to why you are centering the individual when the framework centers on 'Governance and Legislation, with various stakeholder groups around it.*

RESPONSE: Our diagram (Figure 1) visualizes “Governance and Legislation” in the middle and has the individual at the top, in line with the pyramided of trust in SSI relationships. We agree that the figure of speech “putting the individual at the centre” might be confusing in this context. We hence changed the description for the diagram to now read:

We envision a system that, while subject to governance and law, puts the individual at the top of the decision-making process (Figure 1).
