# Supplementary material for: Future-proofing genomic data and consent management: a comprehensive review of technology innovations
Source: Gigascience. 2024 Jun 5;13:giae021. doi: 10.1093/gigascience/giae021 (PMC11152178; doi:10.1093/gigascience/giae021)
Supplement: giae021_Response_to_Reviewer_Comments_Original_Submission [file giae021_response_to_reviewer_comments_original_submission.pdf]

Author's Response To Reviewer Comments

Dear Dr. Nogoy,
Thank you for the support during this process.
We would like to thank the reviewers for their valuable inputs that have substantially improved our manuscript. We have addressed their comments below.
In addition to the requested changes, we also updated the trust triangle (now Figure 1) to differentiate between the typical use cases for genomic data usage (clinical, re-analysis, biobanks) and this was also reflected in the “Proposed Framework” section.

Reviewer #1:
1. The introduction could potentially benefit by being expanded to cover the often conflicting goals & regulatory requirements between research and clinic. This tension complicates the exploration of technical issues from a joint standpoint between these two settings.
RESPONSE: We have elaborated on this in the second paragraph of the introduction, now reading:

The challenges become even more pronounced when applying genomics in a clinical context. Here, the generated data directly impacts patient care while also having value in broadening scientific knowledge. Clinicians, typically rely on pathology providers to generate reports summarising the genomic information to inform patient care. These reports are generated by multidisciplinary teams 15 but in the remaining document we will refer to them as “pathology providers” for simplicity. While established clinical governance and security standards provide guidance for the management, storage, and analysis of genomic data for clinical care, it often conflicts with the need for broader access and sharing of this data for research15 . Platforms able to serve both clinical and research applications need to resolve the tension between the protective regulations in clinical settings and the exploratory objectives of research, as well as enable interoperability across the two domains.

2. Table 1 in the Genomic Data Storage Solutions lists some of the advantages/disadvantages of the different storage options but given that the criteria aren't consistently applied between the solutions makes it very hard to actually compare and it is unclear how these comparisons were made. For example, the "on-premises" solution lists high cost & expertise requirements as a disadvantage, but not only are the costs & expertise requirements never really mentioned for the cloud solutions (which can also be quite high), the costs for the hybrid approach (which probably would be even more expensive due to combining two approaches and requiring more expertise, paying for on premises and cloud etc) aren't listed. Similarly, some of the listed items are not explained in enough detail to be useful (e.g. "trust" as a disadvantage for cloud solutions).
RESPONSE: We updated all tables to compare the different storage, privacy preservation approaches and encryption options against the same criteria. We also provided more context for the individual assessments.

3. Relatedly to the lack of comparability, the main text in this section is very light on citations to support any of the claims regarding the (dis)advantages of any of the solutions.
RESPONSE: We thank the reviewer for pointing this out and have added 13 more citations throughout that part of the text to support our statements. We list these citations below:

• Chee, B. J. S. & Franklin Jr, C. Cloud computing: technologies and strategies of the ubiquitous data center. (CRC Press, 2010).
• Chorafas, D. N. Cloud computing strategies. (CRC press, 2010).
• AWS GovCloud (US) Region – Government Cloud Computing. At: http://aws.amazon.com/govcloud-us/.
• Microsoft AZURE Government. AT https://azure.microsoft.com/en-us/explore/global-infrastructure/government.
• Khan, A. R. & Alnwihel, L. K. A Brief Review on Cloud Computing Authentication Frameworks. Engineering, Technology & Applied Science Research 13, 9997–10004 (2023).
• Rajani, S., Ghorpade, V. & Dhange, M. Multi-factor authentication as a service for cloud data security. Int J Comput Sci Eng 4, 43–46 (2016).

• AWS multi-factor authentication (MFA). AT https://aws.amazon.com/what-is/mfa/.
• Microsoft AZURE MFA. AT https://www.microsoft.com/en-au/security/business/identity-access/microsoft-entra-mfa-multi-factor-authentication.
• Huston, G. AARNet. in The User's Directory of Computer Networks 199–201 (Elsevier, 1990). doi:10.1016/B978-1-55558-047-6.50020-5.
• Liebau, D. & Schueffel, P. Crypto-Currencies and ICOs: Are They Scams? An Empirical Study. SSRN Electronic Journal (2019) doi:10.2139/ssrn.3320884.
• Tiwari, M., Gepp, A. & Kumar, K. The future of raising finance - a new opportunity to commit fraud: a review of initial coin offering (ICOs) scams. Crime Law Soc Change 73, 417–441 (2020).
• Cheah, P. Y. & Piasecki, J. Data Access Committees. BMC Med Ethics 21, 12 (2020).
• Rehm, H. L. et al. GA4GH: International policies and standards for data sharing across genomic research and healthcare. Cell Genomics 1, 100029 (2021).

4. The section includes also a paragraph on the "Genomical" cloud solution, which does not give any reference beyond a link to the companies website and mostly reads like taken from an company advertisement. I notice that one of the co-authors has a conflict of interest as the scientific director of Genomical, which given the framing as a "proven solution" while lacking any reference would be worth to point out in the main text in my opinion.
RESPONSE: We agree with the reviewer. Our intention was to share our lived experience, with Genomical being one example. We already disclosed our conflict of interest and now also changed the paragraph to read:
Finally, building health care critical infrastructure on – for many users – foreign national cloud providers, raises concerns around sovereignty and the limits to oversight, and needs to be carefully balanced against the benefits a globally connected economic health system can provide.
For example, Genomical 49 , one of the first Genomic Information Management Systems (GIMS) designed for clinical genomic data management, is a cloud solution. It benefits from international developments including security, scalability, and health system interoperability, yet seeks to minimise dependence by implementing federated data governance controls. Genomical has thus developed a robust clinical data governance framework and implemented capability for controlled access and reuse of data between authorised entities for the purpose of clinical care, therefore, providing foundations for data reuse within the healthcare system where it is adopted.

5. SImilar to the data storage section, Table 2 in this section is similarly not providing enough structured information to allow the comparison of the different privacy preservation methods. For example, all methods seem to suffer from "data leakage" or "preventing disclosure" etc. but no context is given to aid the reader if there are substantial differences in this seemly shared problem?
RESPONSE: We reworked the table to make the difference clearer. As per response to R1.2 we now compare each method against the same criterion within each table.

6. # Section on Consent models As for the sections above, this subsection lacks citations to support many of its claims. For example, the authors state that broad consent models are problematic for legal/ethical reasons as participants might not understand what they consent to but do not provide evidence for this. Based on the authors' descriptions, it also remains unclear to me if/how tiered consent & "meta consent" differ meaningfully from each other. For the dynamic consent the authors additionally outline that centralised organisations are problematic due to the organisational burden but also because it risks misconduct & misuse, neither which are supported by references.

RESPONSE: We added 4 references supporting our statements about broader consent and centralised organizations being problematic.
We also clarified the difference between tiered and meta consent. The new section now reads as follows:
The dynamic consent model focuses on enhancing continuous engagement of participants through personalised online consent processes (e.g. Dynamic specific consent 138) and digital communication

platforms144–147. It is believed that dynamic consent positively influences both the recruitment and retention of participants as well as their trust towards research148, while also contributing to the proficient management of the informed consent procedure144148.

Building on this, Dynamic meta consent enables participants to define rules to approve or reject studies without needing to decide on each study manually. For example, participants can define their preferences for data use (e.g., academic vs. commercial), data type (e.g., genomic data, medical records, imaging), research institution (e.g., universities, research labs), or funding sources (e.g., public or private)149. This approach provides participants with a fine-grained control over how their data is utilised, yet eliminates the need to manage requests for each individual study. Akin to tiered consent, participants are also required to make upfront decisions at high levels of abstraction without the context for future research studies 138, however they have the flexibility to revise their decision dynamically as new information becomes available or as their preferences change.

The following references have been cited in this updated section:

• Barazzetti, G., Bosisio, F., Koutaissoff, D. & Spencer, B. Broad consent in practice: lessons learned from a hospital-based biobank for prospective research on genomic and medical data. European Journal of Human Genetics 28, 915–924 (2020).
• Mikkelsen, R. B., Gjerris, M., Waldemar, G. & Sandøe, P. Broad consent for biobanks is best–provided it is also deep. BMC Med Ethics 20, 1–12 (2019).
• Mackey, T. K. et al. Establishing a blockchain-enabled Indigenous data sovereignty framework for genomic data. Cell 185, 2626–2631 (2022).
• Zichichi, M., Ferretti, S., D'Angelo, G. & Rodríguez-Doncel, V. Data governance through a multi-dlt architecture in view of the gdpr. Cluster Comput 25, 4515–4542 (2022).

7. Instead, the authors claim that decentralised solutions could overcome these problems through "programatically insured processes" or smart contracts. Benefits of such an approach are stipulated but neither are there supporting references for this, nor do the authors propose any argument for this beyond the claim (as one could envision a centralised consent system which also provides real-time monitoring, revoking consent/data etc).

RESPONSE: We added a paragraph outlining the limitations for a centralised consent system and added supporting references.

Digital consent management is currently delivered predominantly through centralised systems, which facilitate access control, data stewardship, and policy governance. Such centralisation brings the benefits of streamlined management and efficient consent workflows, reducing complexity for organisations. However, it imposes significant burdens on IT systems in synchronising consent changes at all levels of data usage and demands intensive manual processes to demonstrate adherence to compliance standards156,157. This added governance layer may inadvertently create procedural bottlenecks. These bottlenecks can lead to delays and inefficiencies that might not be directly visible to participants but could diminish their overall experience. As a result, there is risk of reduction in participant engagement and participation rate 145 158.

Below we list the supporting references:
• Mackey, T. K. et al. Establishing a blockchain-enabled Indigenous data sovereignty framework for genomic data. Cell 185, 2626–2631 (2022).
• Zichichi, M., Ferretti, S., D'Angelo, G. & Rodríguez-Doncel, V. Data governance through a multi-dlt architecture in view of the gdpr. Cluster Comput 25, 4515–4542 (2022).

8. In the conclusion the authors state that their vision is a decentralised system that does not rely on a central administrative body that centres on the individual "while subject to governance and law". Unfortunately, the authors don't provide either evidence nor a strong argument why such a decentralised system would be needed (or be preferential to the centralised systems).

Overall, the conclusion could lead a reader to the impression that the authors started from this particular

conclusion and built the rest of the review around this, the unfortunately only mention of any technology company in this section being "Genomical" (which I flagged for the conflict of interest above) also contributes to that impression.
RESPONSE: We agree with the reviewer. We have provided a stronger rational for decentralised approaches, removed the mentioning of Genomical in the conclusion and restructured it as a “suggested framework” section. Together with new section addressing the limitations of centralised system (R1.7), we belief this addresses the concern.

9. I think this manuscript would require some substantial work in order to ensure that readers are able to compare the different technologies and requirements for future work more clearly, without getting the impression that the manuscript aims to push a predefined agenda. Providing more context through citations & more structured comparisons would go a great way in achieving this.

RESPONSE: We substantially reworked the tables (same criteria, more information) and several sections (suggested framework, argument why centralised systems have limitations, focused the socio-cultural discussion) to clarify the arguments. This is an important emerging topic, and we believe that this review provides novel arguments and opinions are worth exploring.

Reviewer #2:

1. The abstract lacks clarity. The authors describe how "Genomic information is increasingly used to inform medical treatments and manage future disease risks... personal and societal gains must be carefully balanced against the risk". Which makes a convincing case and offers a framing for the technical content that will follow. However, the authors then describe how "To improve the standard of care and reduce current health disparities, both researchers and clinicians depend on increased participation to genomic studies, especially from underrepresented populations. This requires genomic information management approaches to increase trust and ensuring ethical and culturally appropriate use of an individual's data." The purpose of the paper is unclear - are we looking at a review of risk and mitigations specific to genomics data use, or is this an overview of vulnerable populations that dives into ethics and culture? These are not both equally represented in this draft, perhaps the authors could pick a focus topic and then add to it with the other topic.

RESPONSE: We thank the reviewer for pointing this out and have revised the abstract to be focused on the technical comparison and now reads:
Genomic information is increasingly used to inform medical treatments and manage future disease risks. However, any personal and societal gains must be carefully balanced against the risk to individuals contributing their genomic data. Expanding our understanding of actionable genomic insights requires researchers to access large global datasets to capture the complexity of genomic contribution to diseases. Similarly, clinicians need efficient access to a patient’s genome as well as population-representative historical records for evidence-based decisions. Both researchers and clinicians hence rely on participants to consent to the use of their genomic data, which in turn requires trust in the professional and ethical handling of this information.
Here, we review existing and emerging solutions for secure and effective genomic information management, including storage, encryption, consent, and authorisation that are needed to build participant trust. We discuss recent innovations in cloud computing, quantum-computing-proof encryption, and self-sovereign identity to augment developments from within the genomics community, such as GA4GH Passports and the Crypt4GH file container standard. We also explore how decentralised storage as well as digital consenting process can offer culturally acceptable processes to encourage data contributions from ethnic minorities.
We conclude that the individual and their right for self-determination needs to be put at the centre of any genomics framework; because only on an individual level can the received benefits be accurately balanced against the risk of exposing private information.

2. Technical claims: I would like to compliment the authors on their comprehensive overview of solutions relating to data security (e.g., Table 3 which compares encryption solutions). In reading this draft, there is one use of 'information security' early on, a reference to the 'information security community' and the authors opt for 'AIC' rather than existing terminology of the 'CIA triad'. The authors also mention 'data

security'. It is not clear whether the authors use these terms interchangeably. Such fundamental oversights subtracted from my perception of the authors' authority in the topic of information security; there is little discussion or analysis which is a shame. The topic may provide suitable motivation for their exploration of data-related topics, a useful framework to critique, and basis on which to propose solutions.

RESPONSE: We agree that the interchangeability of the terms needed to be stated and the introductory paragraph now reads:

Irrespective of where genomic data is stored, the individual needs to be protected from unauthorised access to their data (privacy) and the data needs to be kept safe from threats, breaches, unauthorised tampering (security). We explore this topic under the criteria of data availability, integrity, and confidentiality (AIC) 62, sometimes also referred to as the “CIA triad”.

3. For a literature review, I would expect to see a description of how a systematic review had been carried out, and data analysed (e.g., if statistics were gathered from papers in the review). This information has not been offered - as such, the purpose of this draft is unclear, as is any methodology.

RESPONSE: This paper is not a systematic review with pre-determined inclusion/exclusion criteria for structured topics. Instead, it is a literature review aimed at providing a curated overview of an emerging topic of interest. Given the literature is so cross-disciplinary, from a wide range of sources and on a wide range of topics, a systematic review would not have been very practical in our case.

4. The conclusions of this draft are unclear - I apologise to the authors for what may seem to be an unfair statement. There is certainly a 'Conclusion' section. However there are two key issues: first, new material and synthesis is being offered in this section. Perhaps they could simply add a 'Suggested framework' section. Second, there is a lack of cohesion or evidence of a systematic approach to the work. The draft's research questions, purpose and goals are ill-defined, which might normally mean the link to methods is tenuous. As I have already discussed, there does not seem to be any methodology offered at all for how this draft has been generated. I cannot conclude that the conclusions drawn have an empirical basis.
RESPONSE: We agree with the reviewer that adding a "Suggested Framework" section improves the conclusion and have added this accordingly. As per Comment R2.4 we did not aim to present a systematic review.

5. A note of caution: this draft repeatedly cites social and ethical concerns in the pursuit of genomic information management approaches to increase trust and ensuring ethical and culturally appropriate use of an individual's data. I notice that the "unique cultural relationships" of Indigenous communities are mentioned, and that most of the authors are based in Australia. At no point in this draft are recent legislative demonstrations of systematic inequity acknowledged (the recent vote on 'The Voice'). To truly engage with the nature of systemic change, which the authors seem to want to do, is to grapple with the fundamental biases and intentionally hostile policies embodied in health technologies. This relates to my initial comment on the purpose of this paper being unclear. If the authors simply want to review technologies, their social and ethical claims only offer a distraction.

RESPONSE: The focus of the review is indeed on the technologies. However, we recognise that the development and acceptance of technology depend not only on its efficiency and readiness but also on the perceived social and ethical risks associated with it.

In response to the reviewer’s comment, we have removed Figure 1 from the document to avoid the early detour. However, we believe that the remaining discussions on the socio-ethical aspects provide essential context for understanding the rationale behind discussing the more experimental approaches. To further clarify this, we have revised the conclusion:

As genomic sequencing becomes cheaper and more ubiquitous, health and research organisations need to be empowered to access global data assets that are interoperable and scale easily with the application opportunities. This needs to be underpinned by the ethical and trustworthy management of genomic data179 as the security and privacy must be balanced with the need for clinical efficiency and unobstructed research into population-specific care improvements.

However, this balance between protection and utility varies from circumstance to circumstance. It is hence crucial to enable individuals whose genomic data is handled to engage with the process through appropriate consent models and data governance systems. Current centralised data management strategies might get overburdened by scaling up to the level of audit trails or proof of “good processes” required to build trust with participants. Emerging decentralised data and dynamic consent management approaches have sovereignty, self-determination natively enshrined into their approaches. This enables the right to control their own data and utilise culturally-appropriate decision-making models that future participants of genomic data exchanges require.
