# Supplementary material for: Future-proofing genomic data and consent management: a comprehensive review of technology innovations
Source: Gigascience. 2024 Jun 5;13:giae021. doi: 10.1093/gigascience/giae021 (PMC11152178; doi:10.1093/gigascience/giae021)
Supplement: giae021_Response_to_Reviewer_Comments_Revision_1 [file giae021_response_to_reviewer_comments_revision_1.pdf]

Author's Response To Reviewer Comments

Dear Dr. Nogoy,
We are grateful for the guidance provided during the second round of review of our manuscript. Below, we have responded to the specific comments of reviewer 2.
We also used the opportunity to add more information about how GA4GH passports link in with the OAuth 2.0 framework, a central building block of modern web exchanges. The new section reads as follows:
To support automation and interoperability across multiple data systems, the GA4GH has developed the GA4GH Passports and Authentication & Authorisation Infrastructure (AAI) specifications73. Passports provide the format for data and resource access permissions, built on top of the openID Connect standards74, which in turn are based on the OAuth 2.0 framework75. They serve as AAI tokens (utilising JSON Web Tokens) to carry a researcher's digital identity and access rights across organisations, tools, environments, encoding each permission/claim as "Visas", allowing access to specific registered-access datasets. Visas are issued if the intended use of the data complies with restrictions set out by the DAC. Uses and restrictions are based on defined terms in the GA4GH Data Use Ontology (DUO) 76, fully automating the whole process, potentially saving months between an access request and approval.

Reviewer #2:
1) My assessment is that this paper still has issues with its framing and structure. The work lacks clarity in its scope - while you have addressed some of the writing prompts in the reviewer comments, you have not engaged with any substantive suggestions.
RESPONSE: While we have not converted the document into a systematic review as suggested by the reviewer, we outlined our reasons for why we feel a literature review is the more suitable format.

2) The reference to Genomical without any discussion of the market it sits in or competitors in this space is problematic. There is no evidence provided to support the claims made about this company, e.g., a relevant white paper or research paper with statistics, or linking the organization's approach to scientific development or theory.
RESPONSE: We have removed references to the company, and instead referred to their peer-reviewed technology as developed under the public funding of the Australian and Melbourne Genomics Health Alliance, supported by the Victorian Government. The new paragraph reads as following:

For example, Melbourne Genomics Health Alliance, a collaboration of leading Victorian hospitals, research, and academic institutions working with the Victorian Government to embed genomics into the health system, developed a cloud solution that benefits from international developments in security, scalability, and health system interoperability, yet seeks to minimise third-party dependence by implementing federated data governance controls. Specifically, the genomic information management system, Genomical (previously known as GenoVic53) offers a robust clinical data governance framework and implemented capability for controlled access and reuse of data between authorised entities for the purpose of clinical care, therefore, providing foundations for data reuse within the healthcare system where it is adopted.

53. Thorne, N. et al. Genovic: a secure, scalable and modular system for genomic testing. Pathology 51, S120–S121 (2019).

3) The discussion of dynamic consent is based on theory papers that themselves suppose advantages of the model, rather than make claims based in empirical data. Claims are made here without either a) a critical analysis of the literature, or b) evidence to support or refute the claim made.
RESPONSE: We have expanded the theoretical papers on dynamic consent with papers outlining the evidence for dynamic consent. The new section reads as follows and refers to these citations:
The dynamic consent model focuses on enhancing continuous engagement of participants through personalised online consent processes (e.g. Dynamic specific consent 142) and digital communication platforms148–151. It is believed that dynamic consent positively influences both the recruitment and retention of participants as well as their trust towards research148,150,152,153, while also contributing to

the proficient management of the informed consent procedure148.

148. Mascalzoni, D. et al. Ten years of dynamic consent in the CHRIS study: informed consent as a dynamic process. European Journal of Human Genetics 30, 1391–1397 (2022).
151. Teare, H. J. A., Prictor, M. & Kaye, J. Reflections on dynamic consent in biomedical research: the story so far. European journal of human genetics 29, 649–656 (2021).
152. Haas, M. A., Madelli, E. O., Brown, R., Prictor, M. & Boughtwood, T. Evaluation of CTRL: a web application for dynamic consent and engagement with individuals involved in a cardiovascular genetic disorders cohort. European Journal of Human Genetics 1–8 (2023).
153. Spencer, K. et al. Patient perspectives on sharing anonymized personal health data using a digital system for dynamic consent and research feedback: a qualitative study. J Med Internet Res 18, e5011 (2016).

4) I congratulate you on the framework you have produced, but it is unclear as to why you are centering the individual when the framework centers on 'Governance and Legislation, with various stakeholder groups around it.

RESPONSE: Our diagram (Figure 1) visualizes “Governance and Legislation” in the middle and has the individual at the top, in line with the pyramided of trust in SSI relationships. We agree that the figure of speech “putting the individual at the centre” might be confusing in this context. We hence changed the description for the diagram to now read:
We envision a system that, while subject to governance and law, puts the individual at the top of the decision-making process (Figure 1).
