# Supplementary material for: Future-proofing genomic data and consent management: a comprehensive review of technology innovations
Source: Gigascience. 2024 Jun 5;13:giae021. doi: 10.1093/gigascience/giae021 (PMC11152178; doi:10.1093/gigascience/giae021)
Supplement: giae021_Reviewer_1_Report_Original_Submission — Bastian Greshake Tzovaras, Ph.D -- 9/28/2023 [file giae021_reviewer_1_report_original_submission.pdf]

Reviewer Report

Title: Future-proofing genomic data and consent management: a comprehensive review of technology innovations

Version: Original Submission **Date:** 9/28/2023

Reviewer name: Bastian Greshake Tzovaras, Ph.D

Reviewer Comments to Author:

REVIEW: future-proofing genomic data & consent management
The authors present a review article that is aimed at exploring different existing and emerging technical solutions for managing consent and genomic data storage. As the authors convincingly outline in their introduction, this topic will only keep growing in relevance due to the increasing amounts of data and how it could potentially be used in clinical and research settings.
While brief, the introduction of the review does a solid job at providing an overview of how the technical dimensions can interrelate to regulatory and ethical issues. The introduction could potentially benefit by being expanded to cover the often conflicting goals & regulatory requirements between research and clinic. This tension complicates the exploration of techincal issues from a joint standpoint between these two settings.
I fear that in my opinion the main sections (on Data Storage; Data Privacy/Security; and Consent Management) would require some substantial work to be more helpful: Currently, the comparisions between the different approaches remain at a too superficial level to inform others and often give the impression of being based on the authors opinions or personal experiences more than on an in-depth review. I will give some examples below:
Section on Data Storage Solutions
Table 1 in the Genomic Data Storage Solutions lists some of the advantages/disadvantages of the different storage options, but given that the criteria aren't consistently applied between the solutions makes it very hard to actually compare and it is unclear how these comparisons were made. For example, the "on-premises" solution lists high cost & expertise requirements as a disadvantage, but not only are the costs & expertise requirements never really mentioned for the cloud solutions (which can also be quite high), the costs for the hybrid approach (which probably would be even more expensive due to combining two approaches and requiring more expertise, paying for on premises and cloud etc) aren't listed. Similarly, some of the listed items are not explained in enough detail to be useful (e.g. "trust" as a disadvantage for cloud solutions).
Relatedly to the lack of comparability, the main text in this section is very light on citations to support any of the claims regarding the (dis)advantages of any of the solutions. The section includes also a paragraph on the "Genomical" cloud solution, which does not give any reference beyond a link to the companies website and mostly reads like taken from an company advertisement. I notice that one of the co-authors has a conflict of interest as the scientific director of Genomical, which given the framing as a "proven solution" while lacking any reference would be worth to point out in the main text in my opinion.

Data Privacy/Security
SImilar to the data storage section, Table 2 in this section is similarly not providing enough structured information to allow the comparison of the different privacy preservation methods. For example, all methods seem to suffer from "data leakage" or "preventing disclosure" etc. but no context is given to aid the reader if there are substantial differences in this seemly shared problem?
Section on Consent models
As for the sections above, this subsection lacks citations to support many of its claims. For example, the authors state that broad consent models are problematic for legal/ethical reasons as participants might not understand what they consent to but do not provide evidence for this. Based on the authors' descriptions, it also remains unclear to me if/how tiered consent & "meta consent" differ meaningfully from each other. For the dynamic consent the authors additionally outline that centralised organisations are problematic due to the organisational burden but also because it risks misconduct & misuse, neither which are supported by references.
Instead, the authors claim that decentralised solutions could overcome these problems through "programatically insured processes" or smart contracts. Benefits of such an approach are stipulated but neither are there supporting references for this, nor do the authors propose any argument for this beyond the claim (as one could envision a centralised consent system which also provides real-time monitoring, revoking consent/data etc).
Conclusion section
In the conclusion the authors state that their vision is a decentralised system that does not rely on a central administrative body that centres on the individual "while subject to governance and law". Unfortunately, the authors don't provide either evidence nor a strong argument why such a decentralised system would be needed (or be preferential to the centralised systems).
Overall, the conclusion could lead a reader to the impression that the authors started from this particular conclusion and built the rest of the review around this, the unfortunately only mention of any technology company in this section being "Genomical" (which I flagged for the conflict of interest above) also contributes to that impression.
Summary/recommendation
I think this manuscript would require some substantial work in order to ensure that readers are able to compare the different technologies and requirements for future work more clearly, without getting the impression that the manuscript aims to push a predefined agenda. Providing more context through citations & more structured comparisons would go a great way in achieving this.

Methods

Are the methods appropriate to the aims of the study, are they well described, and are necessary controls included? Choose an item.

Conclusions

Are the conclusions adequately supported by the data shown? Choose an item.

Reporting Standards

Does the manuscript adhere to the journal's guidelines on minimum standards of reporting? Choose an item.

Choose an item.

Statistics

Are you able to assess all statistics in the manuscript, including the appropriateness of statistical tests used? Choose an item.

Quality of Written English

Please indicate the quality of language in the manuscript: Choose an item.

Declaration of Competing Interests

Please complete a declaration of competing interests, considering the following questions:

- Have you in the past five years received reimbursements, fees, funding, or salary from an organisation that may in any way gain or lose financially from the publication of this manuscript, either now or in the future?
- Do you hold any stocks or shares in an organisation that may in any way gain or lose financially from the publication of this manuscript, either now or in the future?
- Do you hold or are you currently applying for any patents relating to the content of the manuscript?
- Have you received reimbursements, fees, funding, or salary from an organization that holds or has applied for patents relating to the content of the manuscript?
- Do you have any other financial competing interests?
- Do you have any non-financial competing interests in relation to this paper?

If you can answer no to all of the above, write 'I declare that I have no competing interests' below. If your reply is yes to any, please give details below.

I declare that I have no competing interests

I agree to the open peer review policy of the journal. I understand that my name will be included on my report to the authors and, if the manuscript is accepted for publication, my named report including any attachments I upload will be posted on the website along with the authors' responses. I agree for my report to be made available under an Open Access Creative Commons CC-BY license (http://creativecommons.org/licenses/by/4.0/). I understand that any comments which I do not wish to be included in my named report can be included as confidential comments to the editors, which will not be published.

Choose an item.

To further support our reviewers, we have joined with Publons, where you can gain additional credit to further highlight your hard work (see: https://publons.com/journal/530/gigascience). On publication of this paper, your review will be automatically added to Publons, you can then choose whether or not to claim your Publons credit. I understand this statement.

Yes Choose an item.
