# Supplementary material for: Future-proofing genomic data and consent management: a comprehensive review of technology innovations
Source: Gigascience. 2024 Jun 5;13:giae021. doi: 10.1093/gigascience/giae021 (PMC11152178; doi:10.1093/gigascience/giae021)
Supplement: giae021_Reviewer_1_Report_Revision_1 — Bastian Greshake Tzovaras, Ph.D -- 1/17/2024 [file giae021_reviewer_1_report_revision_1.pdf]

Reviewer Report

Title: Future-proofing genomic data and consent management: a comprehensive review of technology innovations

Version: Revision 1 **Date:** 1/17/2024

Reviewer name: Bastian Greshake Tzovaras, Ph.D

Reviewer Comments to Author:

The authors present an updated and revised version of their review article on future-proofing genomic data & consent management technologies. I want to thank the authors for all the substantial work they put in to revising and refining the article following the feedback they received in the reviews and even going beyond the recommendations. In particular the reframing of the conclusion into a proposed/suggested framework, alongside the revised tables to aid comparisons between approaches and the inclusion of additonal literature as further evidence all drastically are great improvements. Collectively, these changes help readers in understanding both the state of the art as well as the potential that novel combinations of technology can bring in future.
Overall, it seems to me that the difference between the empirical grounding (i.e. the state of the art sections) and the speculative/aspirational future work is now more clear than in the first draft. As such, I think that the authors have adequately addressed the points raised in the first round of reviews.

Methods

Are the methods appropriate to the aims of the study, are they well described, and are necessary controls included? Choose an item.

Conclusions

Are the conclusions adequately supported by the data shown? Choose an item.

Reporting Standards

Does the manuscript adhere to the journal’s guidelines on [minimum standards of reporting?] Choose an item.

Choose an item.

Statistics

Are you able to assess all statistics in the manuscript, including the appropriateness of statistical tests used? Choose an item.

Quality of Written English

Please indicate the quality of language in the manuscript: Choose an item.

Declaration of Competing Interests

Please complete a declaration of competing interests, considering the following questions:

- Have you in the past five years received reimbursements, fees, funding, or salary from an organisation that may in any way gain or lose financially from the publication of this manuscript, either now or in the future?
- Do you hold any stocks or shares in an organisation that may in any way gain or lose financially from the publication of this manuscript, either now or in the future?
- Do you hold or are you currently applying for any patents relating to the content of the manuscript?
- Have you received reimbursements, fees, funding, or salary from an organization that holds or has applied for patents relating to the content of the manuscript?
- Do you have any other financial competing interests?
- Do you have any non-financial competing interests in relation to this paper?

If you can answer no to all of the above, write 'I declare that I have no competing interests' below. If your reply is yes to any, please give details below.

I declare that I have no competing interests

I agree to the open peer review policy of the journal. I understand that my name will be included on my report to the authors and, if the manuscript is accepted for publication, my named report including any attachments I upload will be posted on the website along with the authors' responses. I agree for my report to be made available under an Open Access Creative Commons CC-BY license (http://creativecommons.org/licenses/by/4.0/). I understand that any comments which I do not wish to be included in my named report can be included as confidential comments to the editors, which will not be published.

Choose an item.

To further support our reviewers, we have joined with Publons, where you can gain additional credit to further highlight your hard work (see: https://publons.com/journal/530/gigascience). On publication of this paper, your review will be automatically added to Publons, you can then choose whether or not to claim your Publons credit. I understand this statement.

Yes Choose an item.
