# Supplementary material for: Future-proofing genomic data and consent management: a comprehensive review of technology innovations
Source: Gigascience. 2024 Jun 5;13:giae021. doi: 10.1093/gigascience/giae021 (PMC11152178; doi:10.1093/gigascience/giae021)
Supplement: giae021_Reviewer_2_Report_Original_Submission — Arianna Schuler Scott --10/20/2023 [file giae021_reviewer_2_report_original_submission.pdf]

Reviewer Report

Title: Future-proofing genomic data and consent management: a comprehensive review of technology innovations

Version: Original Submission **Date:** 10/20/2023

Reviewer name: Arianna Schuler Scott

Reviewer Comments to Author:

As a reviewer I would like to thank GigaScience for the opportunity to read about cutting edge ideas in genomics research. I would also like to thank the authors for the ideas, time and effort that has been poured into this manuscript draft. I was asked to review on the basis of specific expertise relating to Dynamic Consent. I offer this as a scope for the following review: I do not have genomics expertise; rather a more technological focus regarding principles of information security and responsible innovation.

I was asked to offer feedback on a Review article, a collection of literature on the topic of: future-proofing genomic data and consent management. As such, the usual GigaScience criteria relating to data collection, sources and analysis do not apply. I was asked to carry out light peer review, offering topics that might add further value.

To summarise, the authors offer an overview of research that shows how information is used in genomics reserach. They highlight an increasing need for data in the provision of care, describing how technical implementations can increase efficient data access. The draft submitted offers a comprehensive overview of a wide range of research. I applaud the authors for gathering this information and putting it together. Gathering information from different disciplines and synthesising conclusions from them is a difficult task.

Research aims: The abstract lacks clarity. The authors describe how "Genomic information is increasingly used to inform medical treatments and manage future disease risks... personal and societal gains must be carefully balanced against the risk". Which makes a convincing case and offers a framing for the technical content that will follow. However, the authors then describe how "To improve the standard of care and reduce current health disparities, both researchers and clinicians depend on increased participation to genomic studies, especially from underrepresented populations. This requires genomic information management approaches to increase trust and ensuring ethical and culturally appropriate use of an individual's data." The purpose of the paper is unclear - are we looking at a review of risk and mitigations specific to genomics data use, or is this an overview of vulnerable populations that dives into ethics and culture? These are not both equally represented in this draft, perhaps the authors could pick a focus topic and then add to it with the other topic.

Technical claims: I would like to compliment the authors on their comprehensive overview of solutions relating to data security (e.g., Table 3 which compares encryption solutions). In reading this draft, there is one use of 'information security' early on, a reference to the 'information security community' and the authors opt for 'AIC' rather than existing terminology of the 'CIA triad'. The authors also mention 'data security'. It is not clear whether the authors use these terms interchangeably. Such fundamental

oversights subtracted from my perception of the authors' authority in the topic of information security; there is little discussion or analysis which is a shame. The topic may provide suitable motivation for their exploration of data-related topics, a useful framework to critique, and basis on which to propose solutions.

Methods: For a literature review, I would expect to see a description of how a systematic review had been carried out, and data analysed (e.g., if statistics were gathered from papers in the review). This information has not been offered - as such, the purpose of this draft is unclear, as is any methodology.

Dynamic consent: I cannot offer much of a critique in this regard. Plenty of relevant literature and projects are cited, which is wonderful to see! One claim, that "Dynamic consent solutions for consumers are needed" is made without any description of why they are needed, or metrics to measure this. These suggestions may be usefully paired with the authors recommendations. Another claim that should be elucidated further is that "Dynamic consent in its current form has gone some way in building community trust". One citation offered, but this is an opportunity to get to the heart of why DC is important. It was not created as a singly technological solution.

Conclusions: The conclusions of this draft are unclear - I apologise to the authors for what may seem to be an unfair statement. There is certainly a 'Conclusion' section. However there are two key issues: first, new material and synthesis is being offered in this section. Perhaps they could simply add a 'Suggested framework' section. Second, there is a lack of cohesion or evidence of a systematic approach to the work. The draft's research questions, purpose and goals are ill-defined, which might normally mean the link to methods is tenuous. As I have already discussed, there does not seem to be any methodology offered at all for how this draft has been generated. I cannot conclude that the conclusions drawn have an empirical basis.

A note of caution: this draft repeatedly cites social and ethical concerns in the pursuit of genomic information management approaches to increase trust and ensuring ethical and culturally appropriate use of an individual's data. I notice that the "unique cultural relationships" of Indigenous communities are mentioned, and that most of the authors are based in Australia. At no point in this draft are recent legislative demonstrations of systematic inequity acknowledged (the recent vote on 'The Voice'). To truly engage with the nature of systemic change, which the authors seem to want to do, is to grapple with the fundamental biases and intentionally hostile policies embodied in health technologies. This relates to my initial comment on the purpose of this paper being unclear. If the authors simply want to review technologies, their social and ethical claims only offer a distraction.

I acknowledge that this review is unlikely to be what the authors want to hear. However, if underlying issues are improved: scope elucidated, research questions better defined, synthesis methodology offered, findings clarified, and conclusions drafted with more clarity, I believe that this work would become a highly cited paper as it brings a great deal of information together.

Methods

Are the methods appropriate to the aims of the study, are they well described, and are necessary controls included? Choose an item.

Conclusions

Are the conclusions adequately supported by the data shown? Choose an item.

Reporting Standards

Does the manuscript adhere to the journal's guidelines on [minimum standards of reporting?] Choose an item.

Choose an item.

Statistics

Are you able to assess all statistics in the manuscript, including the appropriateness of statistical tests used? Choose an item.

Quality of Written English

Please indicate the quality of language in the manuscript: Choose an item.

Declaration of Competing Interests

Please complete a declaration of competing interests, considering the following questions:

- Have you in the past five years received reimbursements, fees, funding, or salary from an organisation that may in any way gain or lose financially from the publication of this manuscript, either now or in the future?
- Do you hold any stocks or shares in an organisation that may in any way gain or lose financially from the publication of this manuscript, either now or in the future?
- Do you hold or are you currently applying for any patents relating to the content of the manuscript?
- Have you received reimbursements, fees, funding, or salary from an organization that holds or has applied for patents relating to the content of the manuscript?
- Do you have any other financial competing interests?
- Do you have any non-financial competing interests in relation to this paper?

If you can answer no to all of the above, write 'I declare that I have no competing interests' below. If your reply is yes to any, please give details below.

I declare that I have no competing interests.

I agree to the open peer review policy of the journal. I understand that my name will be included on my report to the authors and, if the manuscript is accepted for publication, my named report including any attachments I upload will be posted on the website along with the authors' responses. I agree for my report to be made available under an Open Access Creative Commons CC-BY license (http://creativecommons.org/licenses/by/4.0/). I understand that any comments which I do not wish to

be included in my named report can be included as confidential comments to the editors, which will not be published.

Choose an item.

To further support our reviewers, we have joined with Publons, where you can gain additional credit to further highlight your hard work (see: https://publons.com/journal/530/gigascience). On publication of this paper, your review will be automatically added to Publons, you can then choose whether or not to claim your Publons credit. I understand this statement.

Yes Choose an item.
